# Sustainable Synthesis of Bio‐Based Furanic and Aromatic Amines Using an Optimized Whole‐Cell Transaminase–Decarboxylase Cascade in *E. coli* RARE

**DOI:** 10.1002/cbic.70456

**Published:** 2026-07-06

**Authors:** Laura Edit Barabás, Róbert Tőtős, Tímea Éva Csete, Raluca Bianca Tomoiagă, Monica Ioana Toşa, Csaba Paizs

**Affiliations:** ^1^ Department of Enzymology and Applied Biocatalysis Research Center Faculty of Chemistry and Chemical Engineering Babeş‐Bolyai University of Cluj‐Napoca Cluj‐Napoca Romania

**Keywords:** ω‐transaminase, amination, biocatalysis, enzymatic cascade, pyruvate decarboxylase

## Abstract

The development of efficient biocatalytic routes for synthesizing primary amines is often hindered by the unfavorable thermodynamic equilibrium of enzymatic transamination. In this study, we present a highly effective bienzymatic cascade coupling the (*S*)‐selective ω‐transaminase from *Pseudomonas psychrotolerans* (*PpS*‐TA) with pyruvate decarboxylase from *Zymomonas mobilis* (*Zm*PDC). This integrated system uses *Zm*PDC to irreversibly remove the pyruvate coproduct, thereby shifting the equilibrium toward the desired amine. Optimization of the purified enzyme system resulted in a twofold increase in conversion compared to the transaminase alone. The cascade exhibited broad substrate scope across various substituted benzaldehydes and simultaneously enabled the formation of valuable (*R*)‐acyloin byproducts through the carboligation activity of *Zm*PDC. To enhance industrial applicability, the cascade was transferred to a whole‐cell system using an engineered *Escherichia coli* strain (RARE), which minimizes undesired endogenous aldehyde reduction. The coexpressed whole‐cell biocatalyst exhibited superior productivities at substrate loadings up to 50 mM without the need for organic cosolvents. Finally, the utility of this approach was demonstrated through the chemo‐enzymatic synthesis of the bio‐based platform molecule 2,5‐bis(aminomethyl)furan from d‐fructose, achieving an exceptional 90% conversion on a 500‐mL preparative scale. This integrated strategy provides a sustainable and scalable framework for producing high‐value aromatic and furanic amines.

## Graphical abstract

A whole‐cell biocatalytic cascade in engineered *Escherichia* *coli* RARE couples the ω‐transaminase from *Pseudomonas psychrotolerans* with pyruvate decarboxylase from *Zymomonas mobilis* to shift transamination toward amine formation via pyruvate removal. Integration of the cascade into a chemo‐enzymatic synthesis pathway enables the scalable production of 2,5‐bis(aminomethyl)furan from D‐fructose.

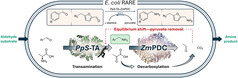

## 1 Introduction

In nature, enzymes function cooperatively within metabolic pathways to convert carbon sources into energy and essential building blocks [1, 2]. Inspired by these biological systems, industrial biocatalysis has increasingly adopted nature‐inspired strategies to enhance process efficiency and selectivity [3, 4, 5, 6]. Within this framework, multienzyme cascades have emerged as powerful tools for modern chemical manufacturing [7], enabling streamlined synthetic routes [8, 9, 10].

Among these biocatalytic tools, transaminases (TAs) have attracted significant interest over the last decade, driven by the global demand for enantiopure chiral amines [11, 12, 13, 14, 15, 16]. TAs are pyridoxal 5′‐phosphate (PLP)‐dependent enzymes that facilitate the transfer of an amino group from a donor to an acceptor molecule. Given their synthetic utility, the discovery and biochemical characterization of novel TAs remain a priority for expanding the enzymatic toolbox [17, 18, 19, 20, 21]. Furthermore, pyruvate decarboxylase (PDC) from *Zymomonas mobilis* represents another industrially relevant biocatalyst. Beyond its established role in bioethanol production [22, 23, 24, 25, 26, 27], PDC is widely employed in enzymatic cascades due to its catalytic versatility [28, 29, 30, 31, 32].

Because enzymatic transamination is an equilibrium‐controlled process, removing coproducts is often essential to achieve high conversion. In TA‐catalyzed reductive aminations using l‐alanine as the amino donor, pyruvate is generated as a coproduct. In this context, PDCs can be employed as secondary enzymes to irreversibly remove pyruvate, thereby shifting the equilibrium toward the desired amine product [33, 34]. While TAs have been extensively evaluated for chiral amine synthesis, prior studies on the reductive amination of aldehydes have largely been restricted to analytical‐scale investigations. These processes typically rely on low substrate concentrations and a large excess of cosubstrates, often lacking preparative‐scale demonstration and isolation of pure products.

We previously identified a novel (*S*)‐selective ω‐transaminase from *Pseudomonas psychrotolerans* (*PpS*‐TA), which exhibited superior catalytic efficiency in the kinetic resolution of racemic 1‐phenylethylamines compared to well‐characterized (*S*)‐TAs [17]. While this enzyme was successfully implemented in a gram‐scale kinetic resolution cascade [35], that route is thermodynamically favorable and does not require an external equilibrium shift. In contrast, the asymmetric synthesis of (hetero)arylalkyl amines from (hetero)aromatic aldehydes remains challenging due to an inherently unfavorable equilibrium.

Recent studies have convincingly demonstrated the power of TA‐based enzymatic and chemoenzymatic cascades for synthesizing chiral amines and related compounds [36, 37, 38, 39]. In contrast to TA‐based processes focused on kinetic resolution or asymmetric synthesis, the present work targets the direct synthesis of nonchiral amines from readily available aldehyde feedstocks. However, these systems are mainly demonstrated on an analytical scale, with limited consideration of process‐relevant constraints such as equilibrium control and product isolation. In contrast, our system employs PDC to actively shift the reaction equilibrium in the transamination of benzaldehydes toward benzylamine formation. Importantly, the applicability of this concept is demonstrated at preparative scale, including isolation of the target products. Furthermore, the developed protocol suppresses alcohol byproduct formation and mitigates Schiff base formation during work‐up, thereby improving overall process robustness.

**SCHEME 1:**
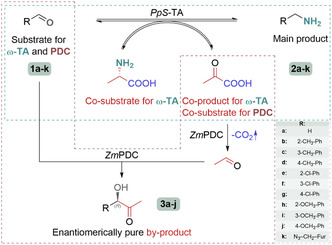
Bienzymatic cascade coupling *PpS*‐TA and *Zm*PDC for the concurrent synthesis of (hetero)arylalkyl amines and enantiopure α‐hydroxy ketones.

In this study, we report the development of an optimized bienzymatic cascade to overcome this thermodynamic limitation. By coupling *PpS*‐TA with *Zm*PDC, we demonstrate a strategy in which the secondary enzyme not only drives the transamination equilibrium by removing pyruvate but also utilizes the unreacted aldehyde in a parallel acyloin (α‐hydroxy ketone) condensation. This approach facilitates the simultaneous conversion of starting materials into two value‐added products: (hetero)arylalkyl amines and enantiopure α‐hydroxy ketones (Scheme 1). Finally, the implementation of this cascade in a whole‐cell system using the engineered *Escherichia coli* RARE strain [40] provides a robust and scalable platform for the sustainable synthesis of bio‐based furanic and aromatic amines.

## 2 Results and Discussion

To optimize the biocatalytic cascade comprising *PpS*‐TA and *Zm*PDC, the individual performance of each biocatalyst was initially evaluated independently under standardized conditions. Benzaldehyde **(1a)** was selected as the model substrate to assess both transamination efficiency and *Zm*PDC‐mediated side reactions. For the transamination step, l‐alanine was employed as the amino donor at a near‐stoichiometric loading (2 equivalents), while sodium pyruvate served as the benchmark substrate for the decarboxylase.

The progress of these preliminary reactions was monitored by quantifying the formation of benzylamine **(2a)** and the corresponding acyloin, respectively (see Section 3.2 for Liquid chromatography–mass spectrometry LC–MS/MS conditions). Following these initial evaluations, the scope of the enzymatic cascade was expanded to include a series of substituted benzaldehydes. The resulting products were isolated (details are provided in Section 3.5.2) and structurally characterized (see Sections S11.1, S11.2, and S12.1), with a particular focus on determining the enantiomeric excess (*ee*) of the secondary products (Section S13 and Table S1). HPLC‐based analytical methods were used throughout to monitor reaction progress and guide the optimization of cascade parameters, ensuring maximum conversion and enantioselectivity (calibration for compounds **1b–k** and **2b–j** is provided in Section 3.3).

### 2.1 Optimization of Reaction Conditions Using Purified Enzymes

#### 2.1.1 Effect of pH on Enzyme Activities

To maximize the yield of the primary product, benzylamine **(2a),** it was essential to establish reaction conditions that harmonized the catalytic requirements of both enzymes. Therefore, the pH‐activity profiles for *PpS*‐TA and *Zm*PDC were evaluated independently.

**FIGURE 1:**
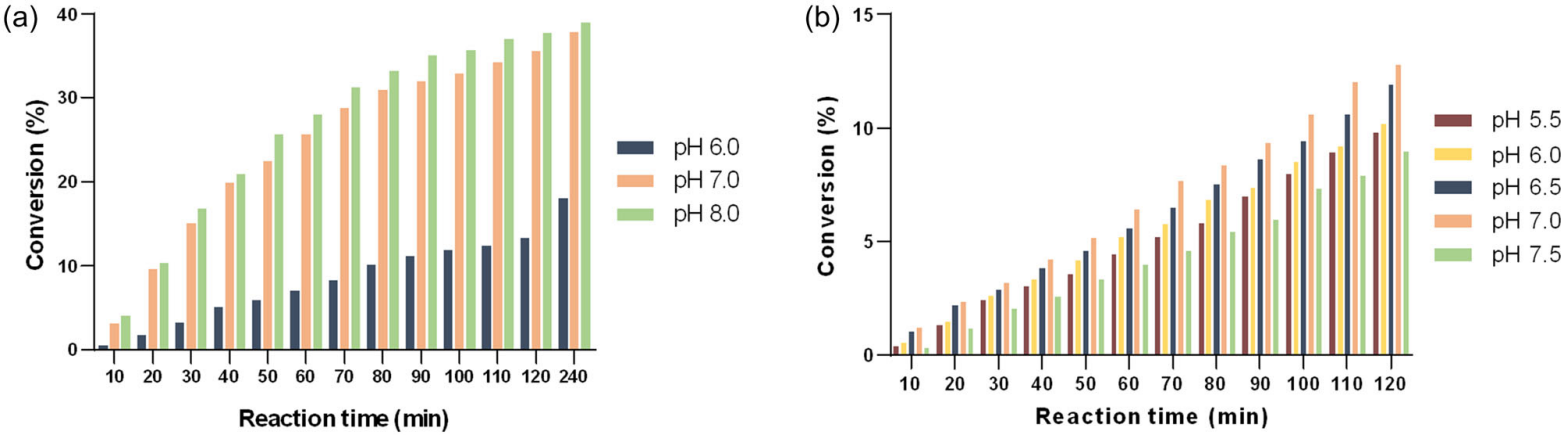
Influence of pH on the catalytic performance of the individual enzymes: (a) conversion of benzaldehyde (20 mM) via *PpS*‐TA‐catalyzed transamination using l‐alanine (40 mM) as the amino donor; (b) conversion of benzaldehyde (20 mM) in the *Zm*PDC‐catalyzed carboligation with sodium pyruvate (40 mM) as the cosubstrate.

For the TA, conversions remained relatively constant between pH 7.0 and 8.0, indicating a broad alkaline tolerance. However, a significant reduction in activity was observed at pH 6.0, where conversion levels dropped to less than 50% of those recorded at higher pH values across all time points (Figure 1a). In contrast, *Zm*PDC exhibited a sharp decline in decarboxylation and condensation activity as the pH increased above 7.0 (Figure 1b).

Considering these divergent profiles, pH 7.0 was selected as the optimal operating point for the cascade. This value represents a critical compromise that maintains robust *PpS*‐TA performance for amine formation while preserving the requisite *Zm*PDC activity to drive the reaction equilibrium through pyruvate removal.

Based on the established pH‐activity profiles, subsequent optimization reactions were conducted in 4‐(2‐hydroxyethyl)‐1‐piperazineethanesulfonic acid (HEPES) buffer (pH 7.0). This buffer was chosen for its suitable *pK*_a_ (7.5 at 20°C) and its minimal interference with PLP‐dependent enzymes compared to primary amine‐based buffers. Under these conditions, the compatibility of *PpS*‐TA and *Zm*PDC was further validated, ensuring that the buffering capacity remained stable throughout the transamination and subsequent decarboxylation steps.

In light of these findings, further test reactions were performed in HEPES buffer (50 mM, pH 7.0).

#### 2.1.2 Effect of Enzyme Amount on Reaction Conversion

To elucidate the reaction kinetics and determine the optimal biocatalyst loading for the conversion of 50 mM benzaldehyde **(1a),** the impact of enzyme concentration on conversion was evaluated as a function of time. Both *PpS*‐TA and *Zm*PDC concentrations were varied from 10 to 100 µg mL^−1^ in 10 µg mL^−1^ increments.

**FIGURE 2:**
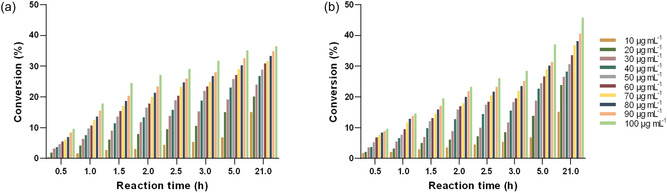
Influence of enzyme loading on conversion over time: (a) transamination of 50 mM benzaldehyde catalyzed by *PpS*‐TA using 100 mM l‐alanine; (b) *Zm*PDC‐catalyzed carboligation of 50 mM benzaldehyde in the presence of 100 mM sodium pyruvate. Concentrations of purified enzymes ranged from 10 to 100 µg mL^−1^.

At the lowest loading (10 µg mL^−1^), transamination proceeded slowly, with conversion increasing steadily but not reaching equilibrium within 5 h; extending the reaction time to 21 h resulted in an increase from ∼7% to ∼15%. In contrast, higher enzyme concentrations facilitated the attainment of chemical equilibrium within 5 h, characterized by a negligible increase in benzylamine **(2a)** formation upon further incubation to 21 h. These results indicate that lower biocatalyst loadings are insufficient to achieve maximum theoretical yields within a standard operational timeframe (Figure 2a).

In the *Zm*PDC‐catalyzed reactions, the formation of the carboligation product (*R*)‐**3a** correlated positively with enzyme concentration. Unlike the TA, *Zm*PDC activity remained significant beyond the 5 h mark even at higher loadings (Figure 2b). At a concentration of 100 µg mL^−1^, the system reached conversions of ∼37% and ∼46% after 5 and 21 h, respectively.

#### 2.1.3 Effect of Substrate Concentration on *PpS*‐TA Activity

**FIGURE 3:**
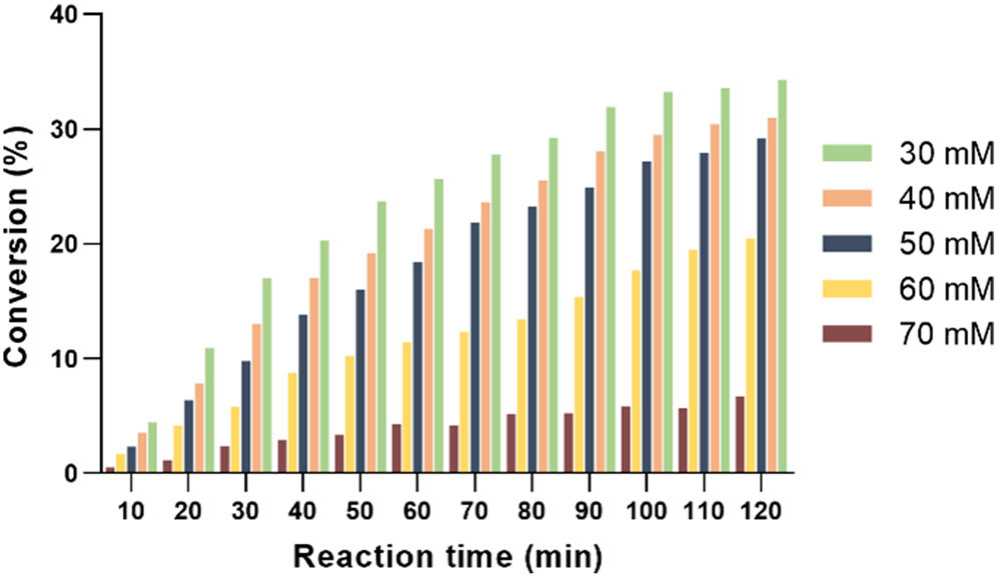
Effect of substrate concentration on the conversion of the *PpS*‐TA‐catalyzed transamination of benzaldehyde at a substrate‐to‐cosubstrate ratio of 1:2.

Evaluation of substrate concentration is critical to identify the threshold at which maximum benzylamine **(2a)** production is achieved without compromising conversion through substrate inhibition. In this study, the transamination of **1a** was investigated at concentrations ranging from 30 to 70 mM (Figure 3).

To facilitate quantitative comparison of the biocatalytic performance at a fixed enzyme loading (100 µg mL^−1^), productivity values were determined based on the formation of **2a** (Table S9).

The data reveal that while percent yield slightly decreases as substrate concentration increases, the absolute titer of **2a** peaks at 50 mM (1.6 mg mL^−1^). Beyond this point, a significant drop in both yield and productivity was observed, likely indicating inhibitory effects. Consequently, 50 mM was identified as the optimal substrate concentration for subsequent cascade evaluations.

#### 2.1.4 Influence of Secondary Enzyme Addition and Loading on Cascade Performance

To evaluate the long‐term stability and synergistic effects of the multienzyme system, the mode of addition for the secondary enzyme, *Zm*PDC, was investigated under two distinct regimes: simultaneous addition (both enzymes at *t* = 0) and sequential addition (*Zm*PDC added after 3 h of transamination). The latter approach ensures a higher initial pyruvate concentration upon introduction of the decarboxylase. Furthermore, the relative loadings of *PpS*‐TA and *Zm*PDC were varied (50 or 100 µg mL^−1^) to optimize the cascade.

**FIGURE 4:**
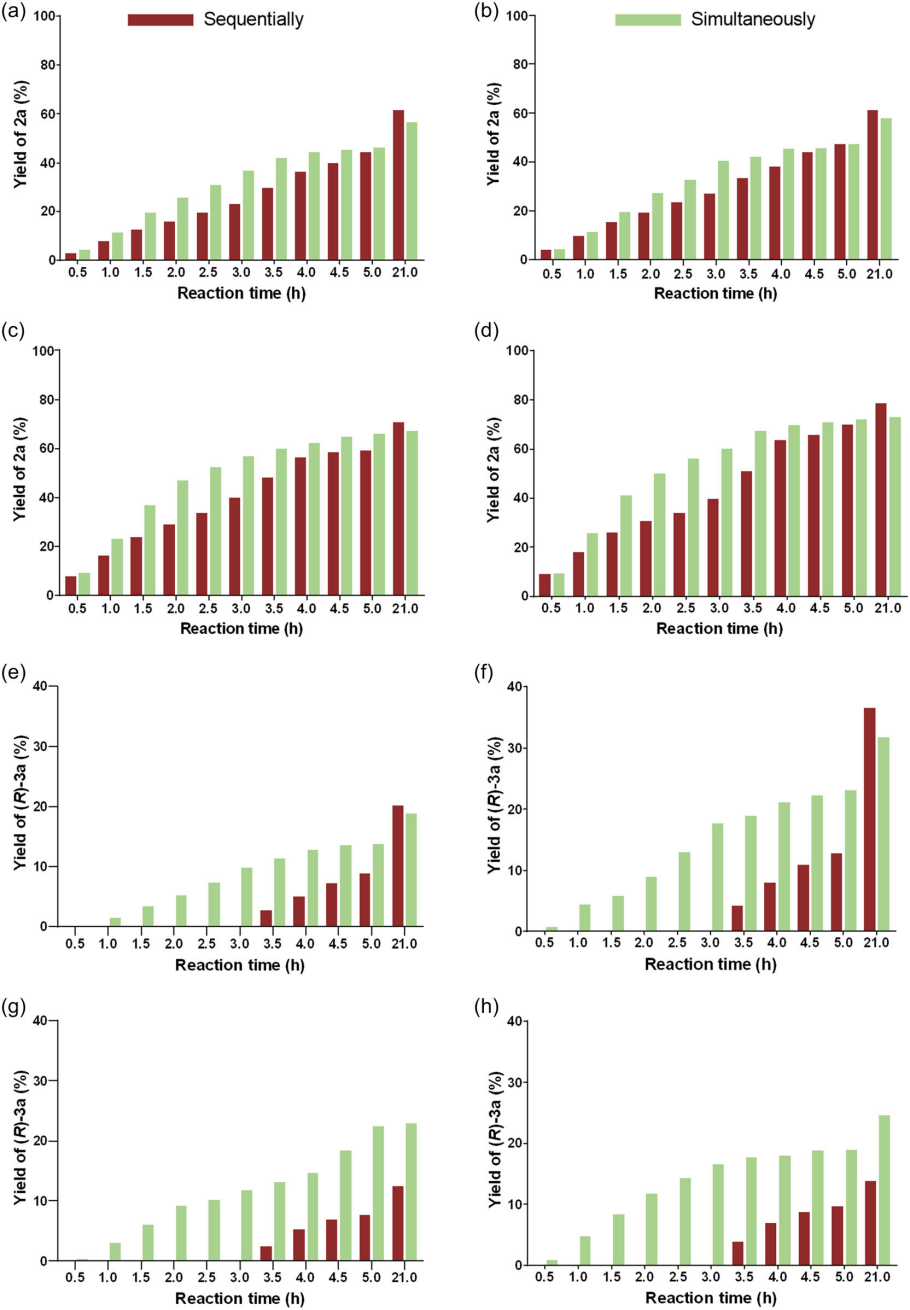
Influence of *Zm*PDC addition mode on benzylamine (2a) formation at varying enzyme loadings: (a) 50/50 µg mL^−1^; (b) 50/100 µg mL^−1^; (c) 100/50 µg mL^−1^; and (d) 100/100 µg mL^−1^ (*PpS*‐TA/*Zm*PDC). Influence of *Zm*PDC addition mode on (*R*)‐phenylacetylcarbinol [(*R*)‐3a] formation at varying enzyme loadings: (e) 50/50 µg mL^−1^; (f) 50/100 µg mL^−1^; (g) 100/50 µg mL^−1^; and (h) 100/100 µg mL^−1^ (*PpS*‐TA/*Zm*PDC).

The formation of **2a** and (*R*)‐**3a** was quantified by LC–MS/MS, as described in Section 3.2. For benzylamine **(2a)** formation, the mode of enzyme addition had negligible impact on final yields after 21 h; however, simultaneous addition improved the short‐term reaction profile (Figure 4a–d). While sequential addition initially resulted in higher yields within the first 5 h, the simultaneous approach proved superior for long‐term productivity. This trend suggests that although delayed addition may preserve *Zm*PDC activity by reducing its total residence time in the buffer, the immediate removal of pyruvate in the simultaneous mode is more effective at driving the transamination equilibrium from the start.

For the formation of the carboligation product (*R*)‐**3a,** simultaneous addition was clearly advantageous (Figure 4e–h). Except for the lowest *PpS*‐TA loading (50 µg mL^−1^), where sequential addition showed a marginal, statistically nonsignificant increase at 21 h, all other conditions yielded significantly higher titers of (*R*)‐**3a** when the enzymes were introduced together.

In conclusion, to maximize the equilibrium shift toward the desired amine, a concurrent loading of 100 µg mL^−1^ for both *PpS*‐TA and *Zm*PDC was established as the optimal configuration.

### 2.2 Performance of the Optimized Cascade System Using Purified Enzymes

**FIGURE 5:**
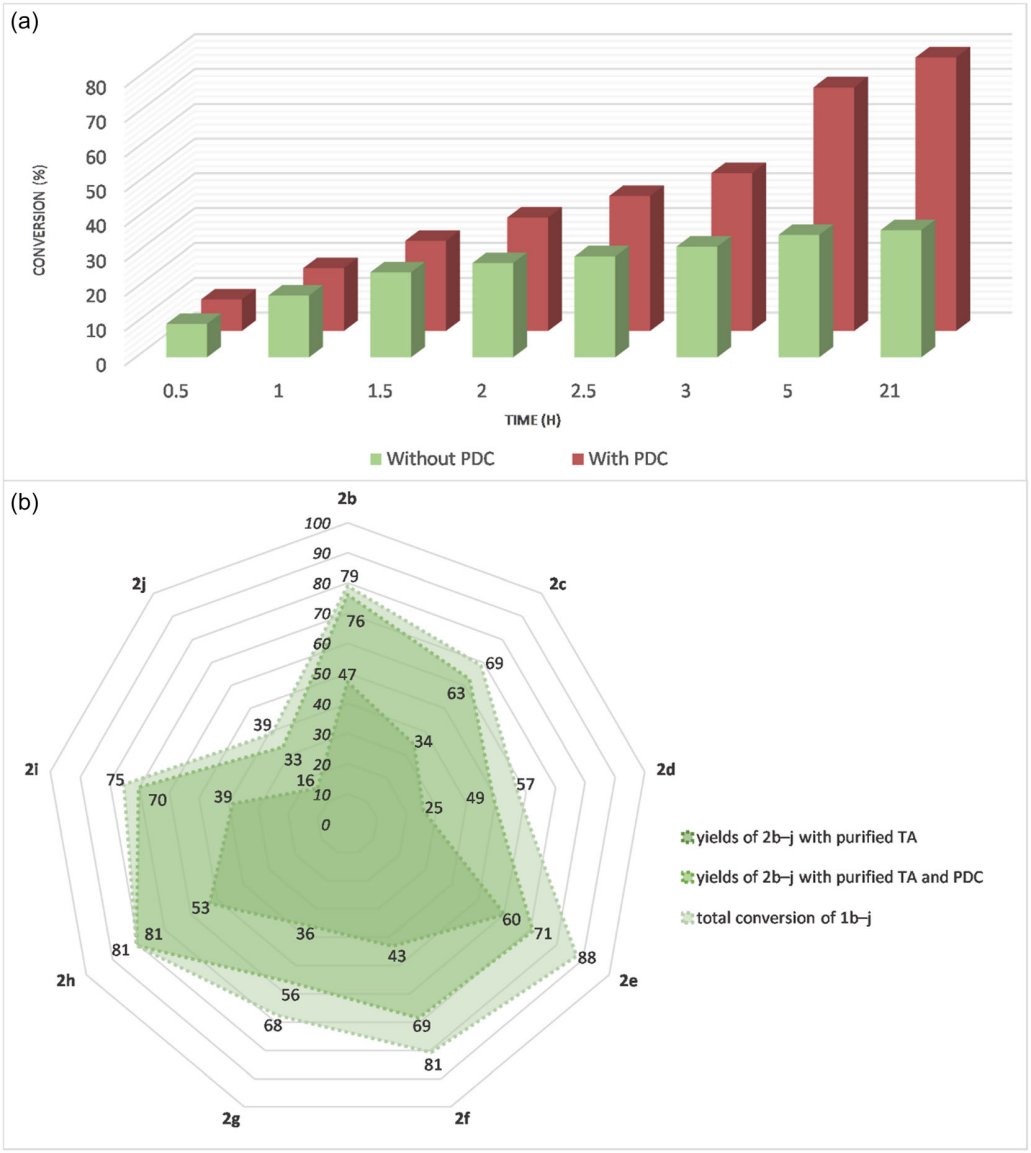
(a) Comparison of benzylamine (2a) formation in single‐enzyme transamination catalyzed by *PpS*‐TA and in the bienzymatic cascade involving *PpS*‐TA and *Zm*PDC; (b) conversion of substituted benzaldehydes (1b–j, 10 mM) after 3 h of reaction time.

The efficiency of the dual‐enzyme system was evaluated by comparing the transamination of **1a** in the presence and absence of *Zm*PDC under optimized conditions (Figure 5a). The results confirm that the integration of the secondary enzyme effectively shifts the reaction equilibrium toward the formation of benzylamine **(2a).**

After 5 h, the cascade system afforded a ∼70% yield of benzylamine, whereas the reaction catalyzed by *PpS*‐TA alone reached only ∼35%, corresponding to a twofold increase in product formation. This disparity was further amplified at the 21 h mark, with the yield of **2a** reaching ∼79% in the cascade system, compared to only ∼37% in the single‐enzyme control. These findings highlight the critical role of *Zm*PDC in driving the equilibrium through the irreversible removal of the pyruvate coproduct.

### 2.3 Biocatalytic Transformation of Substituted Benzaldehydes With Soluble Enzymes

After successful optimization with the model substrate **1a,** the scope of the bienzymatic cascade was expanded to various substituted benzaldehydes **(1b–j).** To ensure complete substrate solubility while minimizing the detrimental effects of organic solvents, a substrate concentration of 10 mM in 10% (v/v) dimethyl sulfoxide (DMSO) was employed for all experiments.

Conversions were quantified by HPLC–UV using benzophenone as the internal standard (high‐performance liquid chromatography (HPLC) methods are provided in the Supporting Information, Tables S2 and S3; the calibration method is described in Section 3.3; two chromatographic methods with different separation lengths were developed to allow selection of the most suitable approach depending on the number of analytes). A clear structure–activity relationship was observed regarding the substitution pattern: *ortho*‐substituted benzaldehydes exhibited the highest conversions, followed by *meta*‐ and *para*‐substituted derivatives (Figure 5b).

The radar chart shows: (inner) transamination conversion without *Zm*PDC; (middle) benzylamine **(2b–j)** formation in the cascade; and (outer) total substrate conversion (sum of amine and acyloin products).

The radar chart analysis indicates that the equilibrium was successfully shifted toward the primary amine product for all substrates tested. However, the extent of the side reaction—acyloin **(3b–j)** formation—varied significantly with the substrate structure. Carboligation was most prominent for chlorobenzaldehydes, specifically yielding 16.7% [(*R*)‐**3e**], 12.2% [(*R*)‐**3f**], and 11.7% [(*R*)‐**3g**]. Conversely, methoxybenzaldehydes yielded the lowest amounts of acyloin byproducts. Notably, the enhancement of benzylamine **(2b–j)** formation was significant across all substrates, confirming that *Zm*PDC‐mediated decarboxylation remains efficient regardless of the substitution pattern.

Since a uniform concentration of 10% DMSO was maintained, these differences in reactivity can be attributed to the inherent substrate specificity and electronic/steric preferences of the enzymes rather than solvent effects. To verify product identities and optical purity of the α‐hydroxy ketones, the primary and secondary products were isolated (Section 3.5.2). Structural integrity was confirmed by ^1^H and ^13^C NMR ( Sections S11.1 and S11.2) and MS (Section S12.1), while the enantiomeric excess (*ee*) of the α‐hydroxy ketones **(3a–j)** was determined by chiral HPLC analysis (Section S13, Table S1).

### 2.4 Whole‐Cell Catalysis Using Cotransformed E. coli RARE Cells

Whole‐cell biocatalysis offers distinct advantages, including a self‐regenerating intracellular environment that maintains enzyme stability, supplies essential cofactors, and eliminates the need for rigorous protein purification. Building on the robust performance of the purified enzymes, a whole‐cell system was developed for the efficient transformation of benzaldehydes **(1a–j)** into their corresponding benzylamines **(2a–j).**

While *PpS*‐TA has previously demonstrated high efficacy in whole‐cell cascades involving the kinetic resolution of chiral amines [35], the current study targets substrates susceptible to endogenous *E. coli* reductases. Aldehydes, in particular, are rapidly metabolized into alcohols or carboxylic acids by wild‐type strains as a detoxification mechanism. For aldehyde substrates, the extent of alcohol byproduct formation was further evaluated as a function of cell loading and reaction time (results are provided in Table S13; calibration curves are shown in Figures S90–S94). To circumvent these competing pathways, *E. coli* RARE (Reduced Aromatic Aldehyde Reduction) was employed [40]. This engineered host lacks multiple endogenous aldehyde reductases and dehydrogenases, providing a “clean” cellular environment for the desired transamination and carboligation reactions.

**FIGURE 6:**
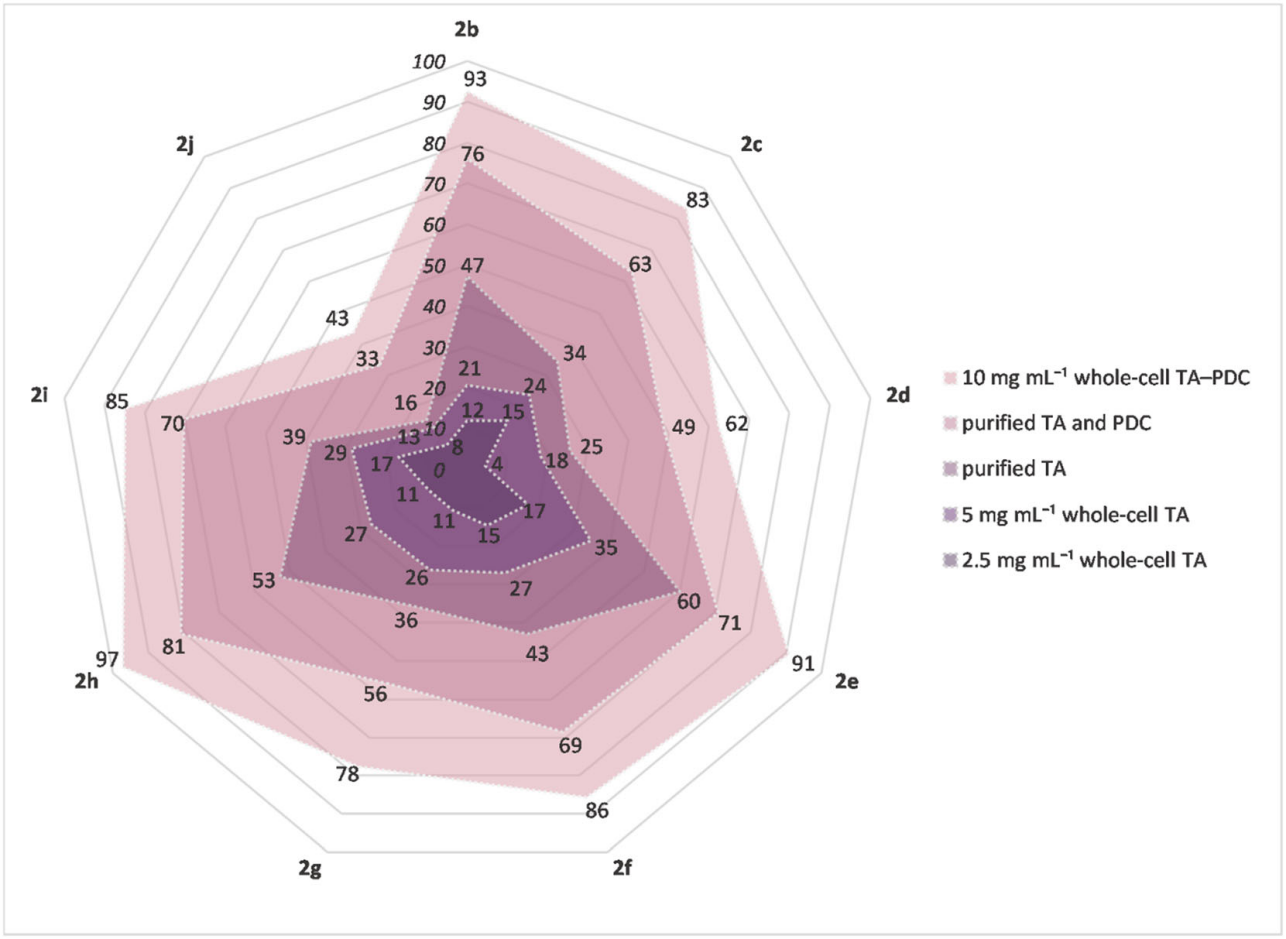
Comparative yield profiles of substituted benzylamines (2b–j) after 3 h under various conditions. Data series represent increasing cell loadings (2.5–10 mg mL^−1^), purified enzyme controls (100 µg mL^−1^), and the optimized coexpressed whole‐cell system at 10 mM substrate concentration.

Initial evaluations were conducted using 10 mM substrate and lyophilized RARE cells overexpressing only *PpS*‐TA at loadings of 2.5 and 5 mg mL^−1^ (Figure 6, inner datasets) in phosphate buffer (PB, 50 mM, pH 7.0). Compared to the purified enzyme controls, significantly lower yields and amine productivities were observed after 3 h, indicating that the reaction had not reached equilibrium. To overcome these limitations, *PpS*‐TA and *Zm*PDC were cotransformed and expressed in the same RARE strain, establishing an integrated intracellular cascade.

Using the coexpressed whole‐cell biocatalyst at a loading of 10 mg mL^−1^, the results exceeded those obtained with purified enzymes (Figure 6, outermost dataset). The substrate scope was selected to include a range of electronic and steric effects. A notable outlier was observed with **1j;** the lower conversion is attributed to the strong electron‐donating mesomeric effect of the *para*‐methoxy group, which reduces the electrophilicity of the carbonyl group. This effect is comparatively diminished in the *ortho* position **(1h)** due to steric and intramolecular interactions.

Encouraged by the robust performance at 10 mM substrate loadings **(1b–j),** the scalability of the whole‐cell system was evaluated by systematically increasing substrate concentrations up to 40 mM in 10 mM increments. For the model substrate **1a,** the concentration range was further expanded from 30 to 70 mM. The objective was to determine the maximum productive capacity of the *PpS*‐TA–*Zm*PDC cotransformed cells under nondilute conditions. Detailed conversion data and corresponding amine titers across all tested concentrations are provided in Table S10.

In this whole‐cell system, the cellular envelope protects the enzymes, allowing DMSO to be omitted as a cosolvent. Despite the reduced solubility of aldehydes in purely aqueous media, no residual substrate was detected at the end of the reactions at higher loadings. This is attributed to the high aqueous solubility of the primary amine products, which drives the dissolution of the remaining aldehyde.

Finally, 200 mL‐scale preparative reactions were performed using the optimized whole‐cell parameters. Under these conditions, acyloin formation was negligible (≤5%), facilitating the streamlined isolation of the target benzylamines. Regarding cofactor requirements, although *E. coli* synthesizes PLP and ThPP endogenously, external supplementation of PLP was necessary to maintain high transamination rates, whereas ThPP supplementation did not significantly influence conversion, suggesting sufficient intracellular levels of ThPP.

While acyloin formation is more pronounced with the purified enzyme, its accumulation in whole‐cell systems remains minimal compared to the amine product. This disparity likely results from differences in effective enzyme activity and the limited instantaneous availability of pyruvate. Nevertheless, pyruvate effectively serves its primary role as a decarboxylation partner. Given the high amine yield, isolating hydroxy ketone byproducts (formed at levels below 5%) is not economically justified for whole‐cell catalysis.

Owing to the distinct chemical characteristics of the products, purification was efficiently achieved using ion‐exchange chromatography followed by liquid–liquid extraction, affording components of high purity with recoveries exceeding 90% (the detailed isolation and purification method for **2a–j** is described in Section 3.5.2), thereby minimizing Schiff base formation between the unreacted aldehyde and the resulting amines.

### 2.5 Extension of the Bienzymatic Cascade to Bio‐Based 2,5‐Bis(aminomethyl)furan

**SCHEME 2:**
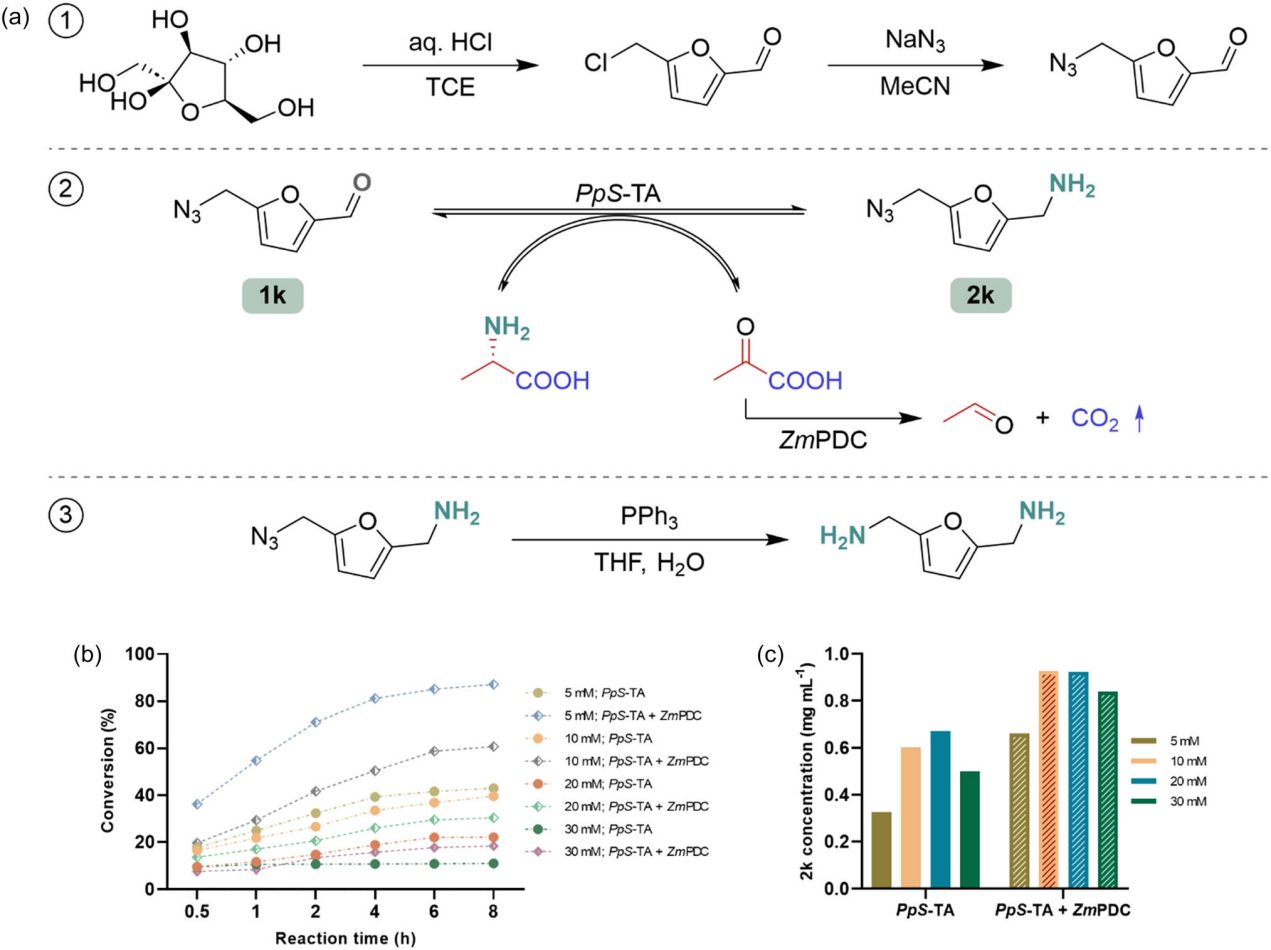
(a) Chemo‐enzymatic synthesis of 2,5‐bis(aminomethyl)furan from d‐fructose, featuring a bienzymatic transamination/decarboxylation step catalyzed by *PpS*‐TA and *Zm*PDC; (b) impact of substrate concentration on conversion for the *PpS*‐TA‐catalyzed transamination of 1k in the absence and presence of the *Zm*PDC cascade; and (c) absolute concentrations of amine 2k achieved after 8 h using purified enzymes (100 µg mL^−1^ each).

Building on the robust performance observed with aromatic substrates, the versatility of the *PpS*‐TA–*Zm*PDC cascade was further evaluated for the synthesis of furan‐based platform molecules. Given their renewable origin from biomass‐derived sugars and their widespread utility in the production of polymers, pharmaceuticals, and fine chemicals, furanic building blocks represent a sustainable alternative to petroleum‐derived aromatics. In light of the growing interest in the transformation of bio‐based furanic and aromatic platform molecules through chemoenzymatic and biocatalytic approaches [41, 42, 43, 44, 45], further investigation of such systems remains highly relevant and warranted. Specifically, the synthesis of (5‐(azidomethyl)furan‐2‐yl)methanamine **(2k)** from 5‐(azidomethyl)furfural **(1k)** was investigated by integrating the established equilibrium‐shifting strategy into a multistep synthetic route starting from d‐fructose (Scheme 2a).

Consistent with the methodology applied to the benzaldehyde series **(1a–j),** initial analytical‐scale reactions were conducted using purified enzymes. As illustrated in Scheme 2b, the implementation of the secondary *Zm*PDC‐catalyzed step significantly enhanced conversion across the entire substrate range (5–30 mM). Notably, while *Zm*PDC effectively removed pyruvate to drive the transamination, no corresponding α‐hydroxy ketone (acyloin) byproduct formation was detected by LC–MS, suggesting that **1k** is not a suitable substrate for the carboligation activity of this particular PDC.

To determine the optimal loading for subsequent whole‐cell catalysis, the titer of **2k** was monitored as a function of initial substrate concentration (Scheme 2c). Maximum productivity was achieved at 10–20 mM. At lower concentration (5 mM), the system was limited by substrate availability, while at 30 mM, a slight decrease in productivity suggested the onset of substrate‐induced inhibition. Consequently, 20 mM was selected as the optimal concentration for preparative‐scale synthesis.

Following the chemical synthesis of substrate **1k** from d‐fructose [46] (see Section S4), a 500 mL‐scale preparative reaction was performed using cotransformed, lyophilized RARE *E. coli* cells (10 mg mL^−1^). Under these optimized whole‐cell conditions, an exceptionally high conversion of 90% was achieved within 4 h (structural characterization of the furan derivatives is provided in Sections S11.3 and S12.2). The isolation and purification of the furanic amine product from the preparative‐scale reaction, affording a product recovery of 95%, are described in Section 3.6.2. This result highlights the industrial potential of the integrated cascade, enabling efficient production of high‐value furanic amines under mild, aqueous conditions with minimal byproduct formation.

**FIGURE 7:**
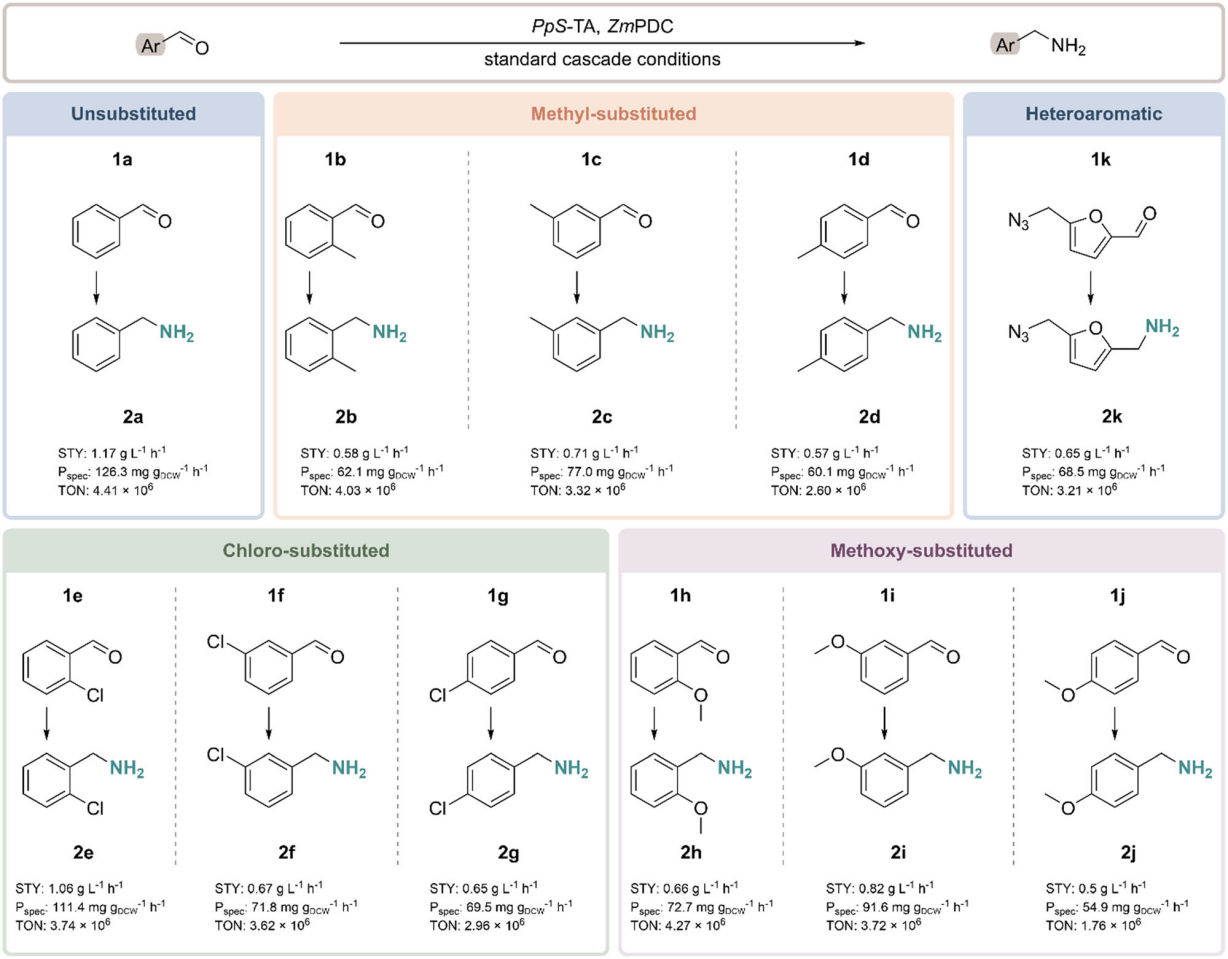
Substrate scope and performance of the *PpS*‐TA–*Zm*PDC cascade.

The cascade consistently exhibits high performance across all investigated aromatic aldehyde substrates (Figure 7), as indicated by favorable STY, specific productivity, and TON values. The underlying experimental data and the corresponding calculated metrics are provided in Tables S14–S18 for both the optimized enzymatic cascade and for conditions where TA activity is limited by an unfavorable reaction equilibrium. Because few systems for aromatic aldehyde amination have been reported, direct benchmarking is challenging. However, compared to literature examples that operate at low substrate concentrations (8–10 mM), use large excesses of cosubstrate, and are limited to analytical‐scale studies [34, 47], the present system demonstrates a robust and practically relevant performance profile. Importantly, the catalyst is a native enzyme system with strong potential for further genetic engineering to enable targeted optimization of aromatic aldehyde transamination within the developed whole‐cell platform and the associated downstream isolation strategy.

## 3 Experimental Section

All experiments were performed in biological triplicate (*n* = 3 independent reactions), each analyzed in technical duplicate. Data are presented as mean values; standard deviations were below 2% of the mean. Error bars are omitted for clarity. The following sections are provided in the Supporting Information: Section 1, Materials and instrumentation; Section 2, Cloning and amplification of *PpS*‐TA and *Zm*PDC genes and transformation of plasmid DNA into *E. coli* cells; Section 3, Recombinant protein expression and purification; and Section 5, Synthesis of racemic α‐hydroxy ketones (*rac*‐**3a–j**).

### 3.1 Optimization of the Bienzymatic Cascade

All enzymatic transformations were conducted at 30°C in 1 mL sealed HPLC glass vials under constant agitation (600 rpm). Reaction progress was monitored by LC–MS/MS using dicyclomine hydrochloride as an internal standard to ensure analytical precision.

#### 3.1.1 Influence of pH on *PpS*‐TA and *Zm*PDC Activity

The pH dependence of *PpS*‐TA was evaluated using **1a** (20 mM), l‐alanine (40 mM), and PLP (0.1 mM) with 50 µg mL^−1^ of enzyme. Similarly, *Zm*PDC activity was assessed using **1a** (20 mM), sodium pyruvate (40 mM), ThPP (0.1 mM), and MgCl_2_ (5 mM) with 50 µg mL^−1^ of enzyme. The buffering systems employed included sodium acetate buffer (50 mM, pH 5.0 and 5.5), phosphate buffer (50 mM, pH 6.0 and 6.5), and HEPES buffer (50 mM, pH 7.0–8.0).

#### 3.1.2 Substrate Loading and Enzyme Dosage

The effect of substrate concentration on *PpS*‐TA (100 µg mL^−1^) was studied in HEPES buffer (50 mM, pH 7.0) by varying **1a** (30–70 mM) and l‐alanine (60–140 mM) concentrations at a fixed 1:2 molar ratio. Enzyme loading effects were independently investigated for both *PpS*‐TA (10–100 µg mL^−1^) and *Zm*PDC (10–100 µg mL^−1^) using 50 mM substrate **1a** and 100 mM of the respective cosubstrate (l‐alanine or sodium pyruvate). PLP and ThPP were each present at 0.1 mM, with MgCl_2_ (5 mM) included in reactions containing ThPP.

#### 3.1.3 Cascade Integration and Secondary Enzyme Loading

To optimize the bienzymatic system, *PpS*‐TA and *Zm*PDC were introduced either sequentially or simultaneously. Various enzyme concentration ratios (50:50, 50:100, 100:50, and 100:100 µg mL^−1^) were screened in HEPES buffer (50 mM, pH 7.0) containing **1a** (50 mM) and l‐alanine (100 mM). PLP and ThPP were each present at 0.1 mM, and reactions containing ThPP additionally included MgCl_2_ (5 mM).

### 3.2 LC–MS/MS Configuration and Parameters

LC–MS/MS analyses were performed on an Agilent 1200 Series HPLC system coupled to an Agilent 6410B triple quadrupole mass spectrometer equipped with an electrospray ionization source operated in positive mode. Quantification was performed using multiple‐reaction monitoring. Calibration curves were established for benzylamine **(2a)** and (*R*)‐phenylacetylcarbinol [(*R*)‐**3a**] over the ranges of 5.36–642.6 ng mL^−1^ **(2a)** and 5.38–645.0 ng mL^−1^ [(*R*)‐**3a**], respectively, corresponding to conversions of 1%–120%. Dicyclomine hydrochloride was used as the internal standard (IS). MS parameters are summarized in Table S11.

The MS source parameters were as follows: capillary voltage, 4 kV; nitrogen gas flow rate, 12 L min^−1^ at 40 psi; nebulizer temperature, 350°C. Chromatographic separation was performed on a Phenomenex Kinetex C18 column (50 × 2.1 mm, 2.6 μm) using isocratic elution with acetonitrile/water (50:50, v/v) containing 0.1% formic acid at a flow rate of 0.3 mL min^−1^. The injection volume was 10 µL, and the column temperature was maintained at 35°C. Under these conditions, the retention times of **2a,** (*R*)‐**3a,** and IS were 0.5, 0.7, and 0.9 min, respectively. The total run time was 4 min (calibration curves are provided in Figures S3 and S4).

### 3.3 HPLC Calibration for Compounds 1b–k and 2b–j

HPLC calibration was performed using benzophenone as the internal standard (IS) and compounds **1b–k** and **2b–j** as analytes. Stock solutions A and B of compounds **1b–k, 2b–j,** and IS were prepared at the concentrations listed in Tables S4–S8. Calibration samples were prepared by adding 40 µL of stock solution B (C_1_–C_7_) of **1b–j** and 40 µL of stock solution B (C_1_–C_7_) of **2b–j** to 895 µL of mobile phase [H_2_O (HClO_4_, pH 1.0)/acetonitrile], followed by the addition of 25 µL of stock solution B of the internal standard. For compound **1k,** an additional dilution step was performed. Specifically, 12 µL of stock solution B (C_1_–C_8_) was taken and diluted to a final volume of 1000 µL with acetonitrile. The calibration samples were then prepared by adding 500 µL of the diluted stock solution B and 450 µL of mobile phase [H_2_O (HClO_4_, pH 1.0)/acetonitrile], followed by the addition of 50 µL of stock solution B of the internal standard. The resulting calibration curves are shown in Figures S5–S14 (Section S9), while representative HPLC chromatograms are provided in Figures S15–S24 (Section S10).

### 3.4 Determination of the Optical Purity of Secondary Products 3a–j

To determine the optical purity of acyloins **(3a–j),** a normal‐phase HPLC method was developed using an Agilent 1200 system. Acyloin‐containing fractions obtained during purification by ion‐exchange chromatography were extracted with an ethyl acetate/*n*‐hexane mixture (3:1, v/v). The combined organic phases were dried over anhydrous Na_2_SO_4_, and the solvent was removed under reduced pressure using a rotary evaporator. The crude products were redissolved in a mixture of *n*‐hexane and isopropanol and analyzed by HPLC. Additional chromatographic separation parameters and the calculated enantiomeric excesses are provided in Table S1. Acyloins can be purified from the crude product by silica gel column chromatography (CH_2_Cl_2_/MeOH, 98:2, v/v) to afford the desired products.

### 3.5 Preparative Whole‐Cell Catalysis with 1a–j

#### 3.5.1 Catalytic Procedures

In 200 mL of 50 mM phosphate buffer (PB) at pH 7.0, the cofactors pyridoxal 5′‐phosphate (PLP, 0.1 mM) and thiamine pyrophosphate (ThPP, 0.1 mM), MgCl_2_ (5 mM), the substrate (**1a** at 50 mM; **1b, 1f–h** at 20 mM; **1c–e, 1i, 1j** at 30 mM), and l‐alanine (at a substrate‐to‐cosubstrate molar ratio of 1:2) were dissolved.

Lyophilized RARE *E. coli* cells coexpressing *PpS*‐TA and *Zm*PDC were then added at a cell loading of 10 mg mL^−1^. The reaction mixtures were incubated at 30 °C with shaking at 250 rpm until completion, as monitored by HPLC. The suspensions were centrifuged at 5000 rpm for 25 min to remove the cell pellets. The pellets were washed with 20 mL PB and centrifuged again under the same conditions. The combined supernatants were filtered through a thin Celite pad to remove residual solids.

#### 3.5.2 Isolation and Purification of 2a–j

The combined reaction supernatants were acidified (pH ∼2.0) with aqueous HCl. The acidic mixtures were subjected to ion‐exchange chromatography using a HiLoad 16/20 column packed with 15 mL of wetted AmberChrom 50WX2 resin, corresponding to an approximate bed height of 8 cm and a wet resin capacity of 0.6 mEq mL^−1^.

The column was equilibrated at pH ∼2.0, and the reaction mixtures were loaded at a flow rate of 2.5 mL min^−1^. The column was washed with bidistilled water, and the amine products were eluted using an aqueous ammonia gradient from 0 to 2 M, as detailed in Table S12, which includes the washing, elution, and re‐equilibration steps.

The collected aqueous fractions containing the amines **2a–j,** with a total volume of ∼60 mL, were extracted three times with 25 mL portions of an ethyl acetate/*n*‐hexane mixture (3:1, v/v). The combined organic phases were dried over anhydrous Na_2_SO_4_, and the solvent was removed under reduced pressure using a rotary evaporator. The isolated amines were obtained in high purity and did not require further purification, with recoveries of 90%–95%. For long‐term storage, the amines were converted to their hydrochloride salts.

### 3.6 Preparative Whole‐Cell Catalysis with 1k

#### 3.6.1 Catalytic Procedure

The substrate (**1k,** 20 mM) was dissolved in 500 mL of 50 mM PB (pH 7.0) containing PLP (0.1 mM), ThPP (0.1 mM), MgCl_2_ (5 mM), and l‐alanine (40 mM). Lyophilized RARE *E. coli* cells coexpressing *PpS*‐TA and *Zm*PDC were added at 10 mg mL^−1^, and the mixture was incubated at 30 °C with shaking at 250 rpm until the reaction was complete, as confirmed by HPLC.

Cells were removed by centrifugation (5000 rpm, 25 min), washed with 40 mL PB, and centrifuged again. The combined supernatants were clarified by filtration through a thin Celite pad.

#### 3.6.2 Isolation and Purification of 2k

For **2k,** isolation was performed using the same ion‐exchange chromatography method described in Section 3.5.2. The aqueous fractions containing **2k** were combined (total volume ∼100 mL), adjusted to pH ∼7.0, and concentrated under reduced pressure by coevaporation with ethanol. The resulting crude product was used directly, without further purification, in the Staudinger reaction for the synthesis of 2,5‐bis(aminomethyl)furan (for the synthesis, see Section S4.3).

## 4 Conclusion

In this study, an efficient bienzymatic cascade was developed by coupling the (*S*)‐selective ω‐transaminase *PpS*‐TA with pyruvate decarboxylase from *Zymomonas mobilis* (*Zm*PDC). This system addresses the thermodynamic limitations inherent to transamination reactions by using *Zm*PDC to irreversibly remove the pyruvate coproduct, effectively driving the equilibrium toward amine formation.

Detailed optimization with purified enzymes identified pH 7.0 and a substrate loading of 50 mM as the optimal parameters for the cascade, resulting in a twofold increase in benzylamine production compared to the single‐enzyme system. The versatility of the cascade was demonstrated across a range of substituted benzaldehydes, where structure–activity relationships revealed that *ortho*‐substituted derivatives achieved the highest conversions. Furthermore, while the cascade primarily shifted the equilibrium toward the target amines, the inherent carboligation activity of *Zm*PDC enabled the simultaneous production of valuable (*R*)‐acyloins, particularly from chlorinated substrates.

The transition from purified enzymes to a whole‐cell biocatalyst in an engineered *E. coli* RARE strain provided a robust and scalable platform. By coexpressing both enzymes, the intracellular environment effectively protected the catalysts and eliminated the need for external cofactor regeneration, achieving conversions up to 90% on a preparative scale. The utility of this approach was further validated through the synthesis of the bio‐based platform molecule 2,5‐bis(aminomethyl)furan from d‐fructose, demonstrating the potential for sustainable, large‐scale chemical manufacturing.

Overall, this integrated bienzymatic strategy offers a green and efficient route for the synthesis of high‐value chiral amines and furanic building blocks, highlighting the power of bioinspired cascades in modern industrial biocatalysis.

## Author Contributions

**Laura Edit Barabás:** writing – original draft, writing – review & editing, methodology, investigation, conceptualization. **Róbert Tőtős:** validation, investigation. **Tímea Éva Csete:** methodology, investigation. **Raluca Bianca Tomoiagă:** methodology. **Monica Ioana Toşa:** formal analysis, data curation, writing – review & editing, resources. **Csaba Paizs:** writing – original draft, writing – review & editing, project administration, data curation, supervision, validation, conceptualization, resources.

## Funding

This study was supported by European Union – NextGenerationEU and the Romanian Government under the National Recovery and Resilience Plan for Romania (760042/23.05.2023).

## Conflicts of Interest

The authors declare no conflicts of interest.

## Supporting information

Supplementary Material

## Data Availability

Research data are not shared.
