# Supplementary material for: Sustainable Synthesis of Bio‐Based Furanic and Aromatic Amines Using an Optimized Whole‐Cell Transaminase–Decarboxylase Cascade in E. coli RARE
Source: Chembiochem. 2026 Jul 6;27(13):e70456. doi: 10.1002/cbic.70456 (PMC13334514; doi:10.1002/cbic.70456)
Supplement: Supplementary file 1 — Supplementary Material [file CBIC-27-e70456-s001.pdf]

Sustainable Synthesis of Bio-Based Furanic and Aromatic Amines Using an Optimized Whole-Cell Transaminase–Decarboxylase Cascade in *E. coli* RARE

Laura Edit Barabás,[+] Róbert Tőtős,[+] Tímea Éva Csete, Raluca Bianca Tomoiagă, Monica Ioana Toşa, Csaba Paizs*

Department of Enzymology and Applied Biocatalysis Research Center
Faculty of Chemistry and Chemical Engineering
Babeș-Bolyai University of Cluj-Napoca
Arany János str. 11
400028 Cluj-Napoca
Romania
E-mail: csaba.paizs@ubbcluj.ro

Table of Contents

1. Materials and instrumentation ... 3

2. Cloning and amplification of *PpS*-TA and *Zm*PDC genes. Transformation of plasmid DNA into *E. coli* cells ... 3

3. Recombinant protein expression and purification ... 4

3.1. *Zm*PDC production ... 4

3.2. *PpS*-TA production ... 5

3.3. SDS-PAGE analysis ... 5

4. Synthesis of furan derivatives ... 5

4.1. Synthesis of 5-(chloromethyl)furan-2-carbaldehyde ... 5

4.2. Synthesis of 5-(azidomethyl)furan-2-carbaldehyde (1k) ... 6

4.3. Synthesis of 2,5-bis(aminomethyl)furan ... 6

5. Synthesis of racemic α-hydroxy ketones (*rac*-3a–j) ... 6

6. HPLC methods ... 7

7. Preparation of HPLC calibrators ... 8

8. LC–MS/MS calibration curves of 2a and 3a ... 9

9. HPLC calibration curves of 1b–k and 2b–j ... 10

10. HPLC chromatograms ... 14

11. ^{1}H and ^{13}C NMR analyses ... 19

11.1. ^{1}H and ^{13}C NMR analyses of 2a–j ... 19

11.2. ^{1}H and ^{13}C NMR analyses of 3a–j ... 31

11.3. ^{1}H and ^{13}C NMR analyses of furan derivatives ... 43

12. Qualitative mass spectrometric analysis ... 47

12.1. Qualitative analysis of compounds 2b–j by mass spectrometry ... 47

12.2. Qualitative analysis of furan derivatives by mass spectrometry ... 49

13. Chiral separation of *rac*-3a–j enantiomers and determination of the optical purity of secondary products in the enzymatic cascade system ... 50

14. Supplementary results ... 55

14.1. Supporting experimental data and analytical conditions ... 55

14.2. Quantification of alcohol byproducts ... 57

14.3. Process performance indicators for biocatalytic aminations ... 59

1. Materials and instrumentation

Commercial chemicals and solvents were obtained from Sigma-Aldrich or Alfa Aesar. LB medium was purchased from Liofilchem, and the protease inhibitor cocktail was obtained from Hoffmann–La Roche. Affinity chromatography was performed using Ni-NTA Superflow resin from Qiagen.

High-performance liquid chromatography (HPLC) analyses were carried out on Agilent 1200 or 1100 Series systems.

^{1}H and ^{13}C NMR spectra were recorded on Bruker 400 MHz and 600 MHz spectrometers, operating at 400 or 600 MHz for ^{1}H NMR and 101 or 151 MHz for ^{13}C NMR, respectively. The following abbreviations indicate signal multiplicities: s for singlet, d for doublet, m for multiplet, and br s for broad singlet.

Mass spectrometric analyses were performed using an Agilent 1200 Series HPLC system coupled to an Agilent 6410B triple quadrupole mass spectrometer equipped with an electrospray ionization (ESI) source operated in positive ion mode.

2. Cloning and amplification of *PpS*-TA and *Zm*PDC genes. Transformation of plasmid DNA into *E. coli* cells

Electrocompetent *Escherichia coli* K-12 MG1655 RARE cells were used for individual and co-transformation with the constructs *PpS-TA*_pET-19b and *ZmPDC*_pCDFDuet-1. The *E. coli* K-12 MG1655 RARE strain was kindly provided by Prof. Kristala Prather (Addgene plasmid #61440).[36] Both genes were cloned using identical restriction sites (*Nde*I and *Xho*I), with *ZmPDC* inserted into the MCS2 region of the pCDFDuet-1 vector.
The gene encoding *PpS*-TA had been previously cloned into the pET-19b vector (Figure S1a), while *Zm*PDC was subcloned from the pET-28a(+) construct into pCDFDuet-1. During the subcloning procedure, both the empty pCDFDuet-1 plasmid and the *ZmPDC*_pET-28a(+) synthetic construct were digested with the corresponding restriction enzymes. The resulting linearized vector and insert fragments were separated on a 1% agarose gel, excised, and purified using the Zymo DNA Clean & Concentrator-5 kit. Ligation was performed using T4 DNA ligase by incubation at 16 °C for 1 h. Both digestion and ligation reactions were performed under conditions recommended by the manufacturer. The ligation mixture was subsequently used to transform *E. coli* XL-1 Blue competent cells via heat shock and plated on LB agar containing 0.05 mg mL^{-1} streptomycin. Randomly selected colonies were screened by colony PCR to confirm the presence of the *PDC* insert using vector-specific primers.

Primer Duet UP1: 5’ – GGATCTCGACGCTCTCCCT – 3’
Primer Duet DOWN1: 5’ – GATTATGCGGCCGTGTACAA – 3’

Electroporation into *E. coli* K-12 MG1655 RARE cells was performed using a 1 mm electroporation cuvette at 1800 V, with 50 µL of electrocompetent cells and 50–80 ng of construct. The mixture was resuspended in 900 µL SOC medium and incubated for 1 h at 37 °C and 200 rpm, followed by plating on LB agar plates containing 0.03 mg mL^{-1} streptomycin and 0.05 mg mL^{-1} carbenicillin.

The co-transformation of the two constructs was performed sequentially. First, 50 µL of electrocompetent *E. coli* K-12 MG1655 RARE cells were transformed with 50–80 ng *PpS-TA*_pET-19b and plated onto an LB agar plate supplemented with 0.05 mg mL^{-1} carbenicillin. Electrocompetent cells prepared from *E. coli* K-12 MG1655 RARE cells containing *PpS-TA*_pET-19b were then transformed with 50–80 ng *ZmPDC*_pCDFDuet-1 (Figure 1b). The presence of both plasmids was confirmed by colony PCR.

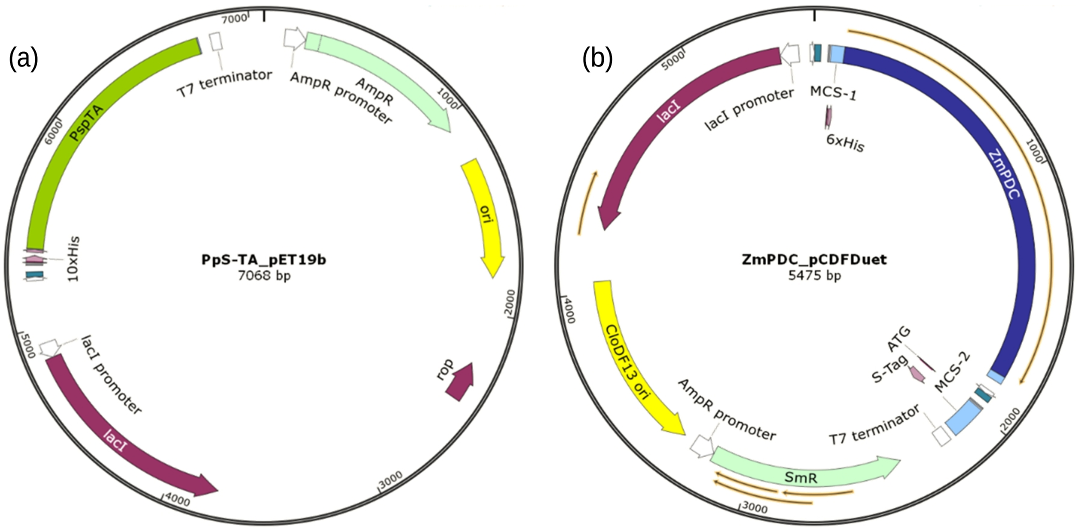

Figure S1. Plasmid map for **(a)** the *PpS-TA*_pET-19b construct and **(b)** the *ZmPDC*_pCDFDuet-1 construct.

3. Recombinant protein expression and purification

3.1. *Zm*PDC production

0.5 L of LB medium was inoculated with 2% (v/v) overnight culture and grown at 37 °C until an optical density (OD_{600}) of 0.6–0.8 was reached. Protein expression was induced by lowering the temperature to 20 °C and adding IPTG to a final concentration of 0.5 mM. Cultures were incubated at 20 °C with shaking at 200 rpm for 17 h. Cells were harvested by centrifugation (5000 rpm, 25 min, 4 °C), resuspended in 50 mM phosphate buffer (pH 7.0), centrifuged again, and lyophilized.

For purification, cells were suspended in 60 mL lysis buffer (50 mM HEPES, 300 mM NaCl, 5 mM imidazole, pH 7.0) supplemented with RNase, lysozyme, and an EDTA-free protease inhibitor tablet. Cells were lysed by sonication (1 500 000 J, 40% amplitude, max. 18 °C), and cell debris was removed by centrifugation (13 000 rpm, 25 min, 4 °C). His-tagged proteins were purified using Ni-NTA affinity chromatography. The supernatant was loaded onto a ~2 cm^3 Ni-NTA column, washed sequentially with low-salt buffer (LS buffer, 50 mM HEPES, 30 mM NaCl, 10 mM imidazole, pH 7.0), high-salt buffer (HS buffer, 50 mM TRIS, 300 mM KCl, pH 8.0), and 25 mM imidazole in LS buffer. The target protein was eluted with 300 mM imidazole in 50 mM HEPES, 30 mM NaCl, pH 7.0. All purification steps were performed on ice. The eluate was dialyzed against 50 mM Tris buffer (pH 7.0) overnight at 4 °C. Protein purity was verified by 12% SDS-PAGE (Figure S2a).

3.2. *PpS*-TA production

Recombinant *PpS*-TA was purified as previously described.[17,35]

3.3. SDS-PAGE analysis

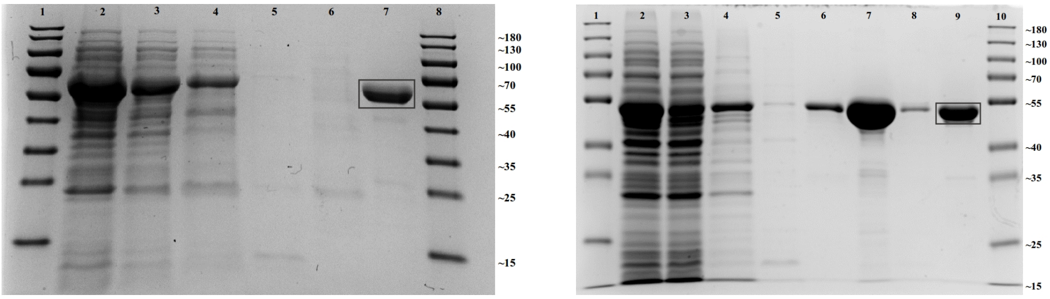

(a) **(b)**

Figure S2. (a) SDS-PAGE analysis on a 12% gel showing the purification of recombinant *Zm*PDC using a Ni-NTA column. **Lane 1:** protein mass marker (Thermo Scientific™ PageRuler™ Prestained Protein Ladder); **lane 2:** supernatant from cell lysate; **lane 3:** flow-through; **lane 4:** wash with low-salt buffer (LS solution: 50 mM HEPES, 30 mM NaCl, 10 mM imidazole, pH 7.0); **lane 5:** wash with high-salt buffer (HS solution: 50 mM Tris, 300 mM KCl, pH 8.0); **lane 6:** wash with 20 mM imidazole (20 mM imidazole in LS); **lane 7:** *Zm*PDC after dialysis; **lane 8:** protein mass marker (Thermo Scientific™ PageRuler™ Prestained Protein Ladder). **(b)** SDS-PAGE analysis on a 12% gel showing the purification of recombinant *PpS*-TA using a Ni-NTA column. **Lane 1:** protein mass marker (Thermo Scientific™ PageRuler™ Prestained Protein Ladder); **lane 2:** supernatant from cell lysate; **lane 3:** flow-through; **lane 4:** wash with low-salt buffer (LS solution: 50 mM HEPES, 30 mM NaCl, 10 mM imidazole, pH 7.0); **lane 5:** wash with high-salt buffer (HS solution: 50 mM Tris, 300 mM KCl, pH 8.0); **lane 6:** wash with 25 mM imidazole (25 mM imidazole in LS); **lane 7:** *PpS*-TA eluted with 300 mM imidazole; **lane 8:** wash with 1 M imidazole; **lane 9:** *PpS*-TA after dialysis; **lane 10:** protein mass marker (Thermo Scientific™ PageRuler™ Prestained Protein Ladder).

4. Synthesis of furan derivatives

4.1. Synthesis of 5-(chloromethyl)furan-2-carbaldehyde

According to a modified version of the reported general procedure,[37] a paste was prepared from D-fructose (9 g, 50 mmol) and water (1.8 mL). Trichloroethylene (250 mL) was added to this suspension, under an inert atmosphere. The reaction mixture was heated to 75 °C with vigorous stirring. After reaching the target temperature, concentrated hydrochloric acid (12.5 mL, 0.15 mol) was added in one portion. The reaction was maintained at 75 °C for 1 hour. After completion, the mixture was filtered, and the organic phase containing CMF was separated. The aqueous phase was extracted with trichloroethylene (3 × 50 mL). The combined organic layers were washed with saturated aqueous sodium bicarbonate, dried, and filtered. Trichloroethylene was removed under reduced pressure using a rotary evaporator. The crude product was redissolved in dichloromethane and filtered through a thin silica gel pad. After removal of dichloromethane under reduced pressure, the residue was purified by vacuum distillation (83 °C at 2 mbar) to afford 5-(chloromethyl)furfural as a colorless liquid in 85% yield.

4.2. Synthesis of 5-(azidomethyl)furan-2-carbaldehyde (1k)

Freshly distilled CMF (5 g, 34.6 mmol) was dissolved in acetonitrile (100 mL). Sodium azide (4.5 g, 69.2 mmol), a few drops of water, and catalytic amounts of potassium iodide and 18-crown-6 (10–20 mol%) were added. The reaction mixture was stirred at 60 °C overnight. After completion, acetonitrile was removed under reduced pressure, and the residue was redissolved in chloroform and filtered. The filtrate was passed through a short silica gel pad, and chloroform was removed under reduced pressure to afford pure 5-(azidomethyl)furan-2-carbaldehyde **(1k)** in 97% yield**.**

4.3. Synthesis of 2,5-bis(aminomethyl)furan

(5-(Azidomethyl)furan-2-yl)methanamine (**2k,** 913 mg, 6 mmol) and triphenylphosphine (1.9 g, 7.2 mmol) were dissolved in THF (80 mL) under an inert atmosphere. The reaction mixture was stirred at room temperature for 2 hours, after which water (3 mL) was added, and stirring was continued for an additional 3 hours. After completion, THF was removed under reduced pressure, and water (15 mL) was added to the residue. The resulting mixture was filtered, and the filtrate was extracted with toluene (3 × 5 mL). The aqueous phase was purified by ion exchange chromatography on AmberChrom^{TM} 50WX2 resin using the same procedure described for compound **2k,** (see Section 3.7.2 in the main text) affording a product recovery of 93%.

5. Synthesis of racemic α-hydroxy ketones (*rac*-3a–j)

To a mixture of the corresponding aldehyde (**1a–j,** 3.0 mmol), trimethylsilyl cyanide (3.9 mmol, 1.3 equiv.), and THF (75 µL), lithium chloride (0.23 mmol, 10 mg) was added in catalytic amount. The reaction mixture was stirred at room temperature until complete conversion of the aldehyde to the corresponding TMS-protected cyanohydrin (~0.5 h, monitored by TLC). The reaction was then quenched with *n*-hexane (10 mL) and extracted with aqueous sodium carbonate solution (1 M, 3 × 10 mL). The organic phase was dried over anhydrous Na_2SO_4, filtered, and the solvent was removed under reduced pressure. The resulting crude TMS-protected cyanohydrin was used directly in the next step without further purification.

To a stirred solution of methylmagnesium iodide (6.0 mmol, 2.0 equiv.) in diethyl ether (30 mL), a solution of the previously obtained TMS-protected cyanohydrin in diethyl ether (10 mL) was added dropwise at room temperature. The resulting mixture was refluxed at 40 °C for 4 hours. After cooling to room temperature, the reaction mixture was poured, under vigorous stirring, into ice-cold aqueous hydrochloric acid (5%, 50 mL). The mixture was stirred for an additional 1–4 hours, after which the layers were separated. The aqueous phase was extracted with diethyl ether (3 × 30 mL). The combined organic layers were dried over anhydrous Na_2SO_4, filtered, and the solvent was removed under reduced pressure. The crude products were purified by silica gel column chromatography (CH_2Cl_2/MeOH, 98:2, v/v) to afford the corresponding acyloins *rac*-**3a–j** (η ~80–89%).

6. HPLC methods

Table S1. HPLC method parameters for the chiral separation of racemic α-hydroxy ketones (*rac*-**3a–j**).

Compound	Mobile phase composition		Temperature (°C)	Type of HPLC column*	$t_r(R)$ (min)	$t_r(S)$ (min)	*ee* (%)
	% (v/v) *n*-hexane	% (v/v) IPA					
**3a**	90	10	0	Phenomenex Lux Cellulose-3	8.8	9.8	91
**3b**	92	8	0	Phenomenex Lux i-Cellulose-5	14.9	15.9	86
**3c**	97	3	0		20.3	23.2	94
**3d**	92	8	0		12.7	14.9	99
**3e**	95	5	0		12.1	12.9	89
**3f**	95	5	0		8.9	9.9	93
**3g**	95	5	0		9.9	10.9	97
**3h**	90	10	25	Phenomenex Lux Cellulose-3	11.9	15.5	n.d.
**3i**	80	20	0	Phenomenex Lux i-Cellulose-5	7.4	10.5	74
**3j**	80	20	20		6.8	7.3	>99

*Phenomenex Lux® 5 µm Cellulose-3, 250 x 4.6 mm and Phenomenex Lux® 3 µm i-Cellulose-5, 150 x 4.6 mm

Table S2. HPLC method parameters for the separation of compounds **1b–k, 2b–k,** and benzophenone (internal standard).

Compound	Mobile phase composition		Temperature (°C)	Flow (mL min^{-1})	Type of HPLC column	Retention time (min)		
	% (v/v) H_2O ($HClO_4$, pH 1.0)	% (v/v) Acetonitrile				aldehyde	IS	amine
**1,2b**	40	60	25	0.4	Daicel CROWNPAK® CR-I(+), 3 × 150 mm, 5 µm	3.1	4.1	7.5
**1,2c**	40	60	25	0.4		3.1	4.1	8.2
**1,2d**	40	60	25	0.4		3.1	4.1	7.1
**1,2e**	40	60	25	0.4		3.3	4.2	8.9
**1,2f**	40	60	25	0.4		3.4	4.2	8.1
**1,2g**	40	60	25	0.4		3.3	4.2	6.9
**1,2h**	45	55	25	0.4		3.3	4.9	12.6
**1,2i**	45	55	25	0.4		3.3	4.9	7.1
**1,2j**	45	55	25	0.4		3.2	4.9	7.7
**1,2k**	40	60	30	0.4		3.1	4.6	8.9

Table S3. HPLC method parameters for the separation of compounds **1b–j, 2b–j, 3b–j,** and benzophenone (internal standard).

Compound	Mobile phase composition		Temperature (°C)	Flow (mL min^{-1})	Type of HPLC column	Retention time (min)			
	%(v/v) H_2O ($HClO_4$, pH 1.0)	%(v/v) Acetonitrile				acyloin	aldehyde	IS	amine
**1–3b**	67	33	35	0.4	Daicel CROWNPAK® CR-I(+), 3 × 150 mm, 5 µm	3.8	4.8	7.9	8.9
**1–3c**	78	22	35	0.4		4.9	7.1	18.6	15.3
**1–3d**	76	24	35	0.4		4.7	6.3	15.3	12.3
**1–3e**	65	35	35	0.4		4.3	4.9	6.9	9.7
**1–3f**	65	35	35	0.4		4.2	4.9	6.9	9.4
**1–3g**	65	35	35	0.4		4.2	4.9	6.9	7.9
**1–3h**	72	28	35	0.4		3.9	5.1	11.2	14.5
**1–3i**	72	28	35	0.4		3.9	5.1	11.2	8.6
**1–3j**	78	22	35	0.4		4.3	5.9	11.3	19.6

7. Preparation of HPLC calibrators

Table S4. Preparation of stock solutions **A** for compounds **1b–k** and **2b–j.**

Compound	Mass of analyte (mg)	Volume Stock A (mL)	Concentration (mg mL^{-1})	Used Solvent
**1b–d**	12.015	10	1.2	HEPES (50 mM, pH 7.0)
**1e–g**	14.057		1.41	
**1h–j**	13.615		1.36	
**1k**	105.791		10.57	
**2b–d**	12.118		1.21	
**2e–g**	14.16		1.42	
**2h–j**	13.718		1.37	

Table S5. Preparation of stock solutions **B** for compounds **1b–j** and **2b–j.**

Stock C_n	Volume Stock A (µL)	Volume Stock B (mL)	Concentration of 2b–j (µg mL^{-1})			Concentration of 1b–j (µg mL^{-1})		
			2b–d	2e–g	2h–j	1b–d	1e–g	1h–j
**Stock C_1**	10	2	6.059	7.08	6.859	6.0075	7.0285	6.8075
**Stock C_2**	30		18.177	21.24	20.577	18.0225	21.0855	20.4225
**Stock C_3**	80		48.472	56.64	54.872	48.06	56.228	54.46
**Stock C_4**	200		121.18	141.6	137.18	120.15	140.57	136.15
**Stock C_5**	400		242.36	283.2	274.36	240.3	281.14	272.3
**Stock C_6**	800		484.72	566.4	548.72	480.6	562.28	544.6
**Stock C_7**	1200		727.08	849.6	823.08	720.9	843.42	816.9

Table S6. Preparation of stock solutions **B** for compound **1k.**

Stock C_n	Volume Stock A (µL)	Volume Stock B (mL)	Concentration of 1k (µg mL^{-1})
**Stock C_1**	14.28	2	75.565
**Stock C_2**	42.86		226.695
**Stock C_3**	85.71		453.39
**Stock C_4**	171.43		906.78
**Stock C_5**	342.85		1813.56
**Stock C_6**	714.28		3778.25
**Stock C_7**	1000		5289.55
**Stock C_8**	1571.43		8312.15

Table S7. Preparation of stock solution **A** for internal standard (IS).

Substance	Amount of analyte (mg)	Volume Stock A (mL)	Concentration (mg mL^{-1})	Used Solvent
**IS**	182.22	10	18.22	H_2O/MeCN (50:50)

Table S8. Preparation of stock solution **B** for internal standard (IS).

Stock C_n	Volume Stock A (µL)	Final Volume (mL)	Concentration of IS (µg mL^{-1})
**Stock C_{IS}**	100	10	182.22

8. LC–MS/MS calibration curves of 2a and 3a

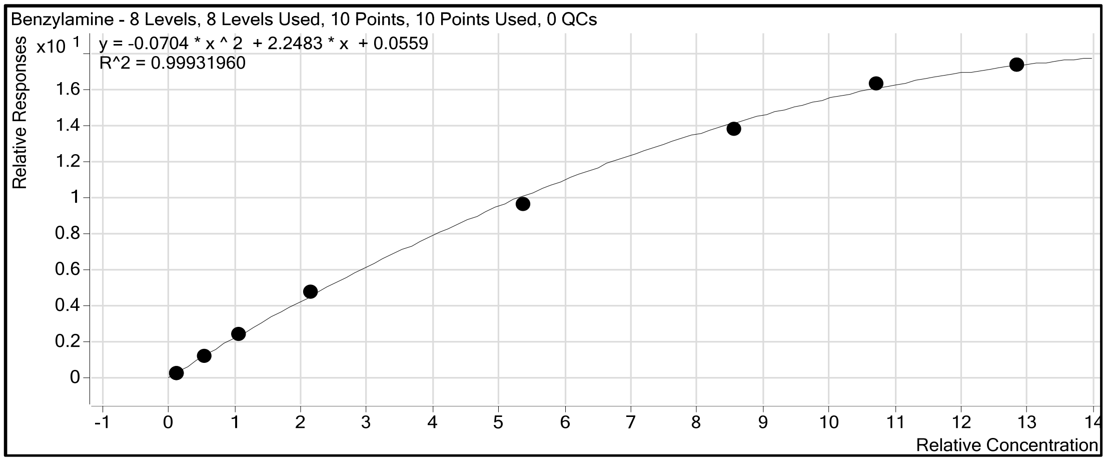

Figure S3. Calibration curve for **2a** using dicyclomine hydrochloride as IS.

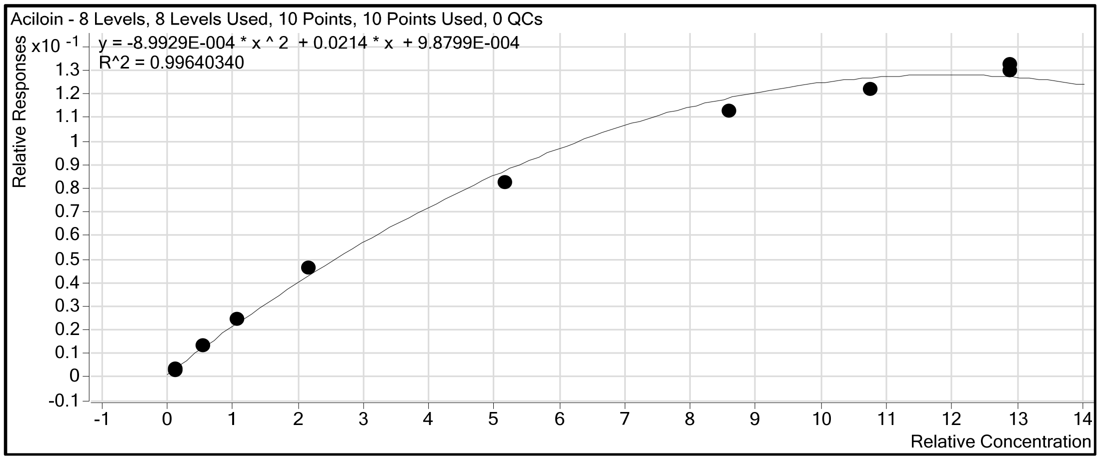

Figure S4. Calibration curve for **3a** using dicyclomine hydrochloride as IS.

9. HPLC calibration curves of 1b–k and 2b–j

The detection wavelengths are as follows: 210 nm for **1e, 1h, 2b–f, 2h,** and IS; 220 nm for **1i, 1j, 2g,** and **2i;** 230 nm for **2j;** 250 nm for **1b–d, 1f,** and **1g;** and 277 nm for **1k.**

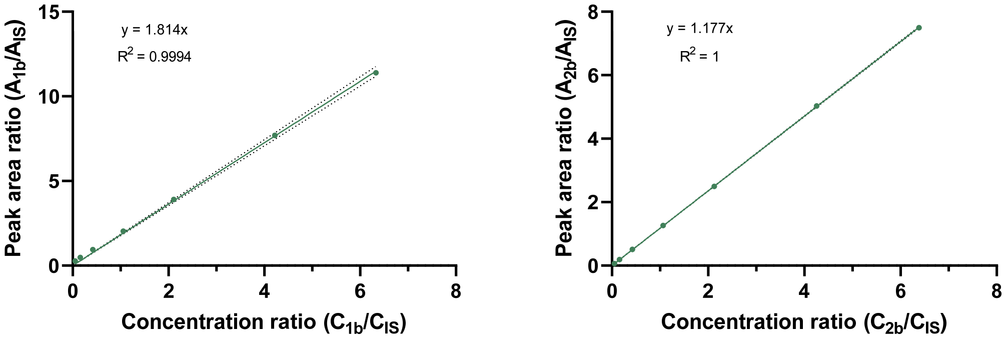

(a) **(b)**

Figure S5. Calibration curve for **(a) 1b** and **(b) 2b** using benzophenone as IS.

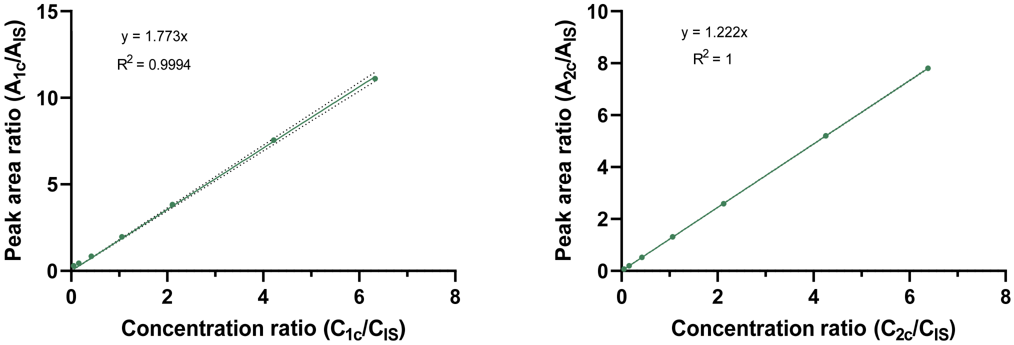

(a) **(b)**

Figure S6. Calibration curve for **(a) 1c** and **(b) 2c** using benzophenone as IS.

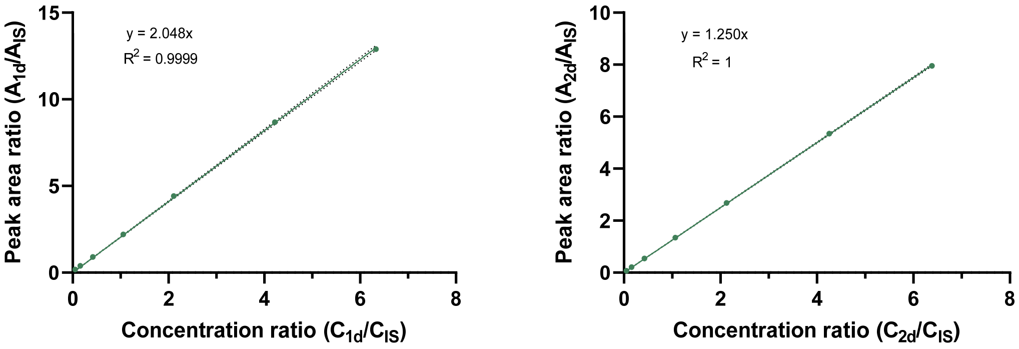

(a) **(b)**

Figure S7. Calibration curve for **(a) 1d** and **(b) 2d** using benzophenone as IS.

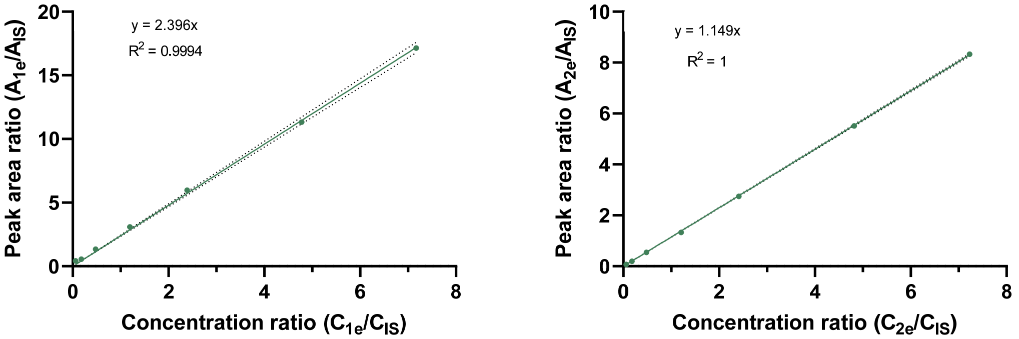

(a) **(b)**

Figure S8. Calibration curve for **(a) 1e** and **(b) 2e** using benzophenone as IS.

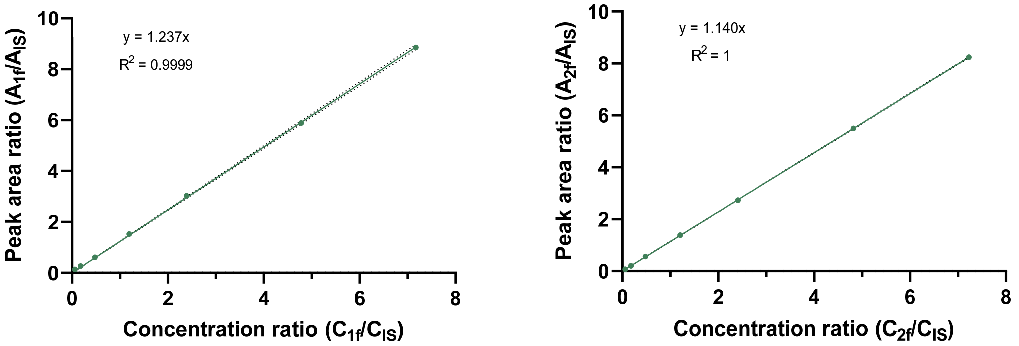

(a) **(b)**

Figure S9. Calibration curve for **(a) 1f** and **(b) 2f** using benzophenone as IS.

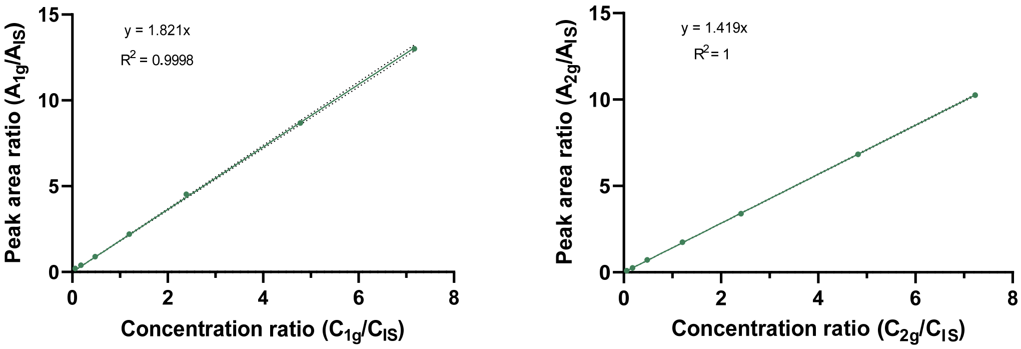

(a) **(b)**

Figure S10. Calibration curve for **(a) 1g** and **(b) 2g** using benzophenone as IS.

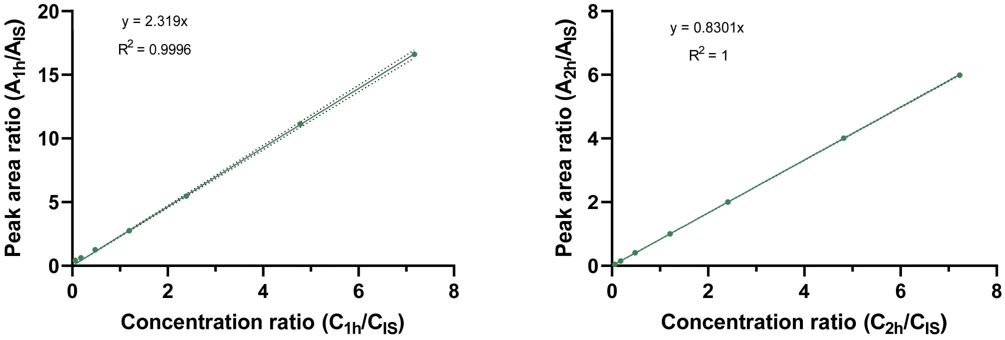

(a) **(b)**

Figure S11. Calibration curve for **(a) 1h** and **(b) 2h** using benzophenone as IS.

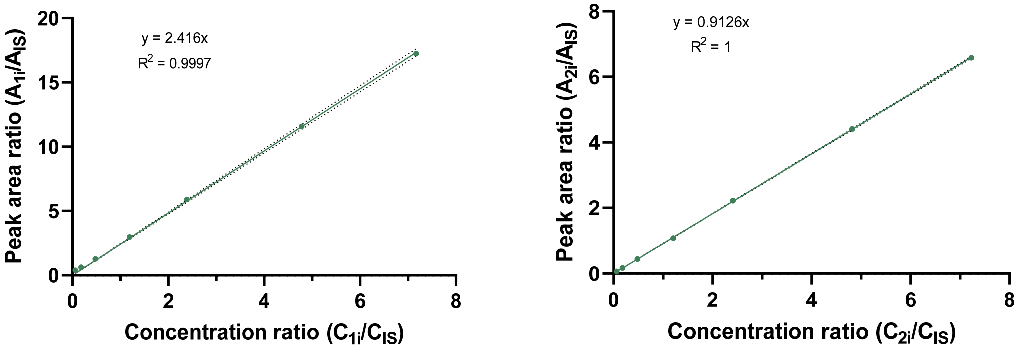

(a) **(b)**

Figure S12. Calibration curve for **(a) 1i** and **(b) 2i** using benzophenone as IS.

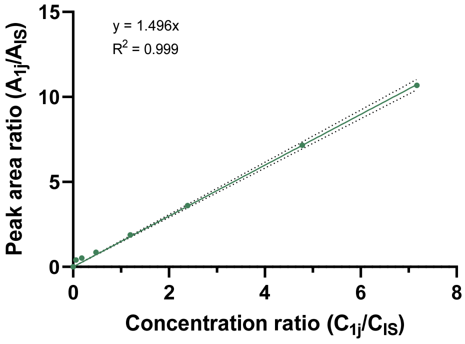

(a)

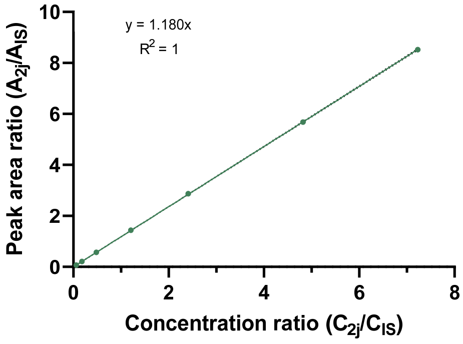

(b)

Figure S13. Calibration curve for **(a) 1j** and **(b) 2j** using benzophenone as IS.

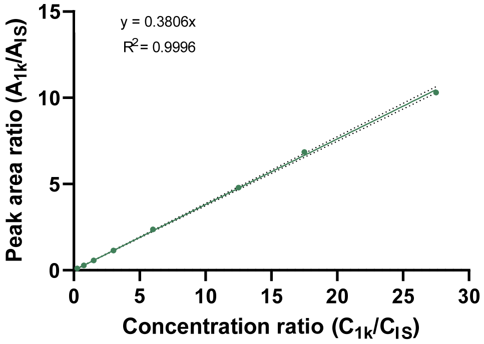

Figure S14. Calibration curve for **1k** using benzophenone as IS.

10. HPLC chromatograms

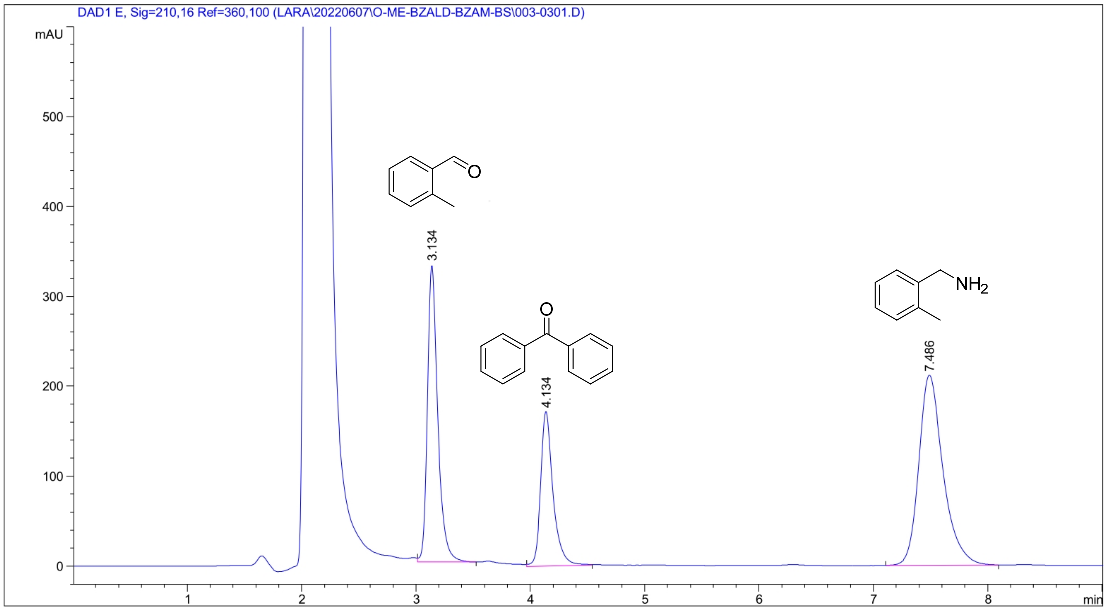

Figure S15. Elution diagram on a Crownpak CR-I(+) column for **1b** and **2b** using benzophenone as IS.

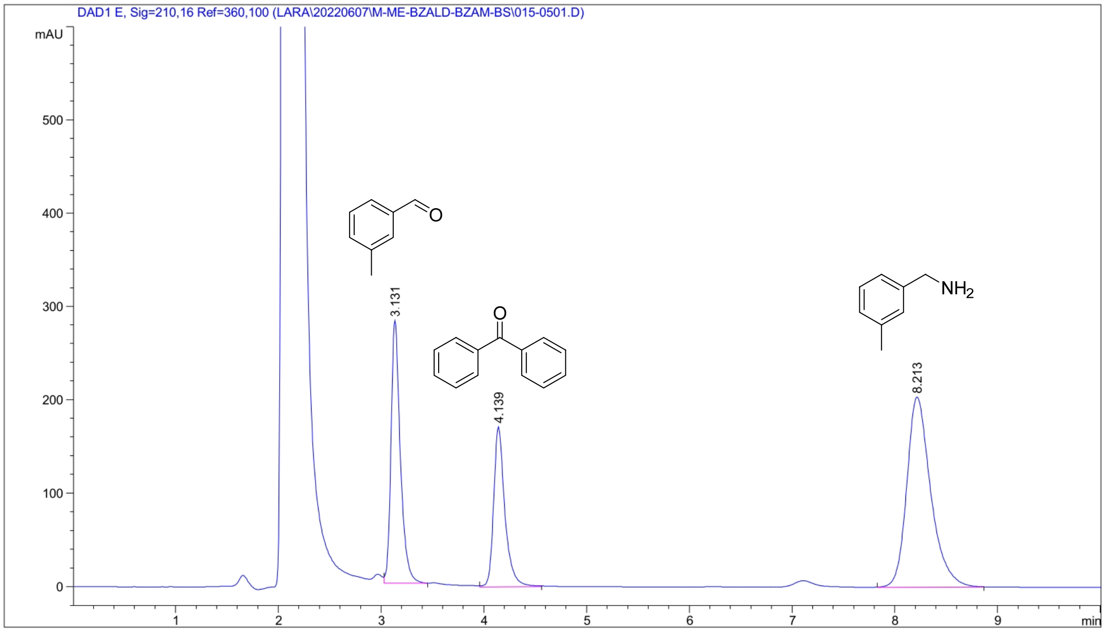

Figure S16. Elution diagram on a Crownpak CR-I(+) column for **1c** and **2c** using benzophenone as IS.

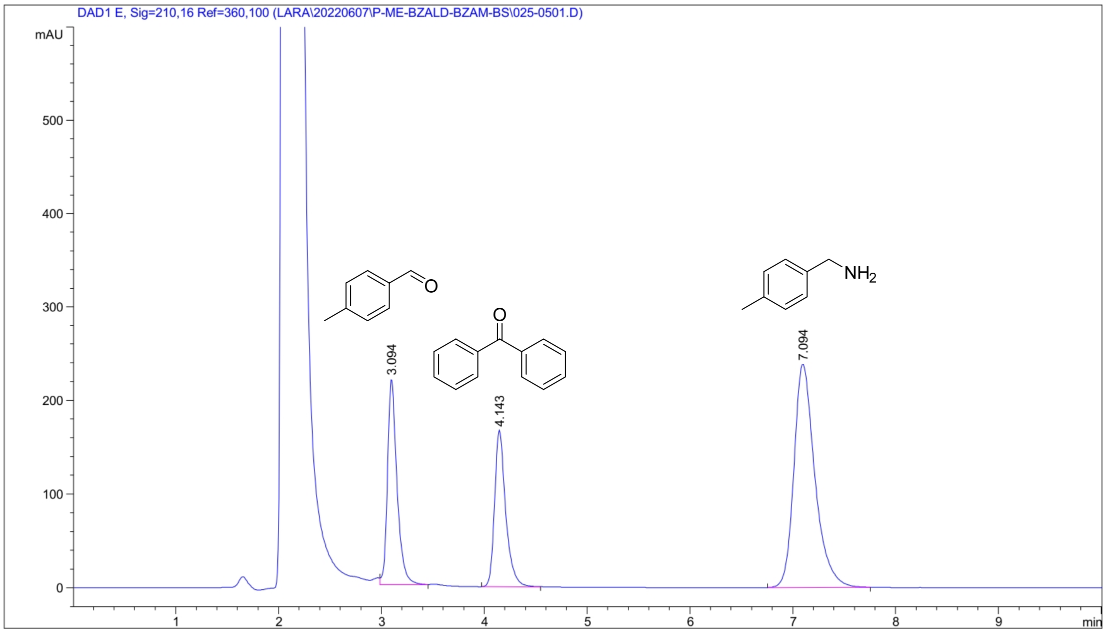

Figure S17. Elution diagram on a Crownpak CR-I(+) column for **1d** and **2d** using benzophenone as IS.

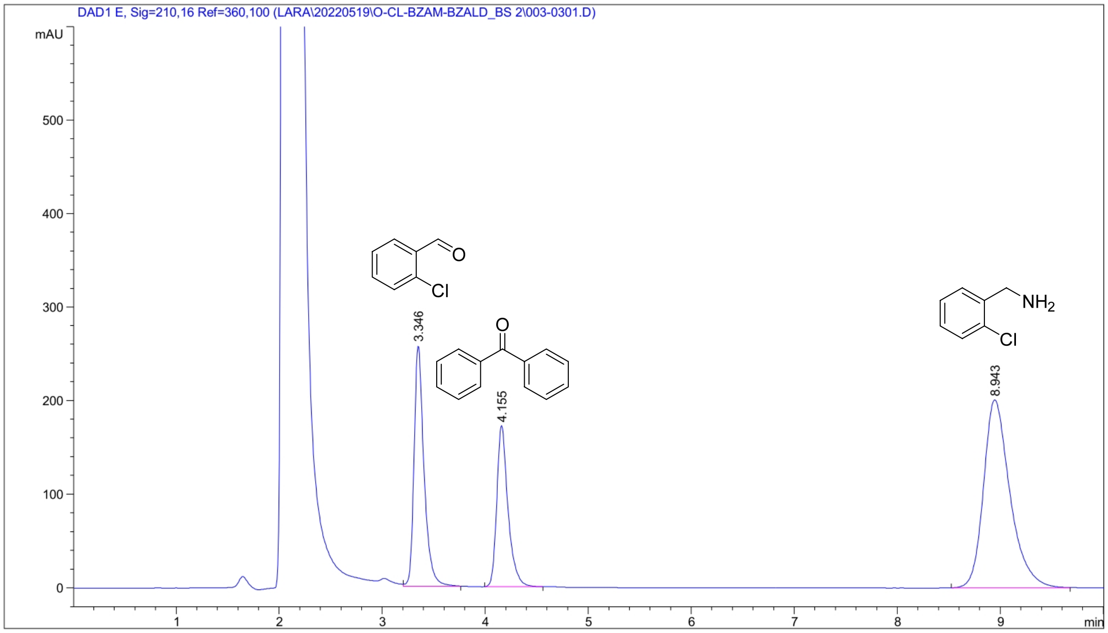

Figure S18. Elution diagram on a Crownpak CR-I(+) column for **1e** and **2e** using benzophenone as IS.

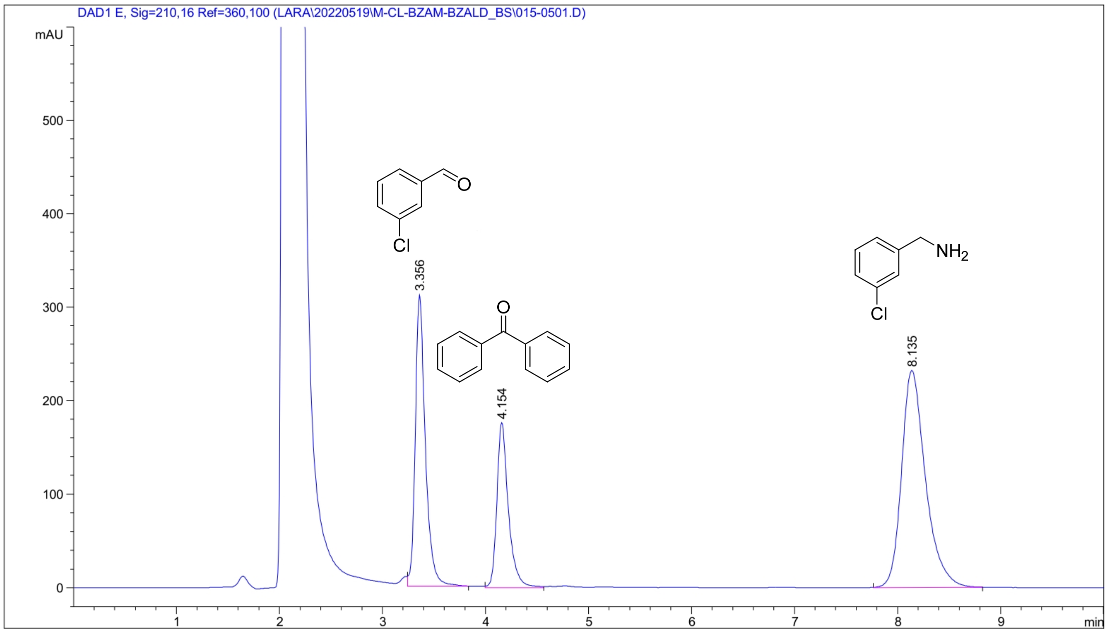

Figure S19. Elution diagram on a Crownpak CR-I(+) column for **1f** and **2f** using benzophenone as IS.

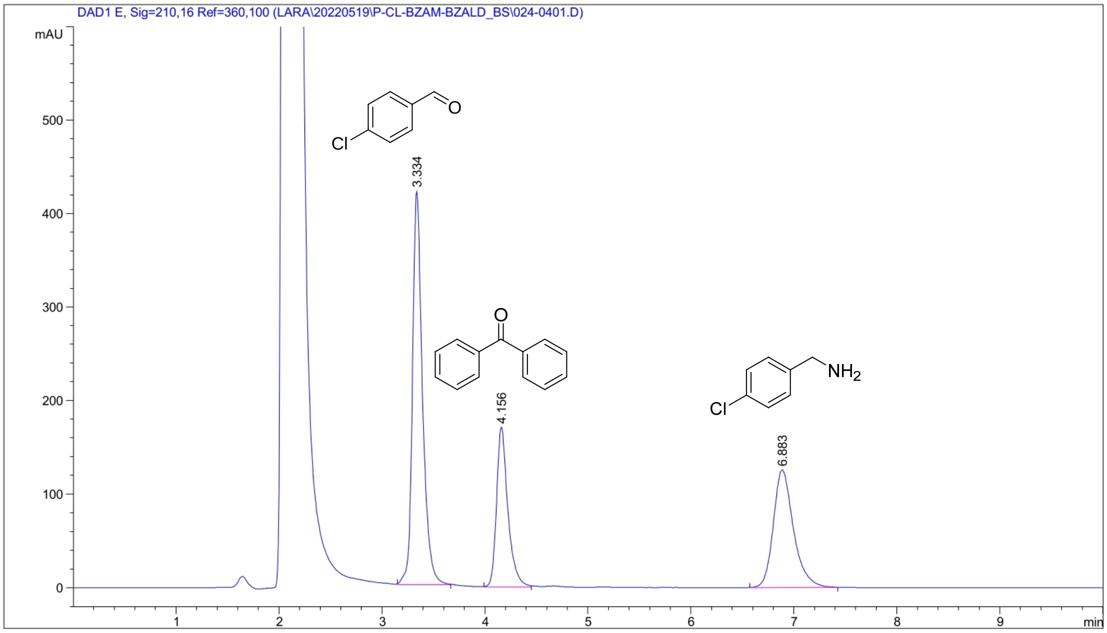

Figure S20. Elution diagram on a Crownpak CR-I(+) column for **1g** and **2g** using benzophenone as IS.

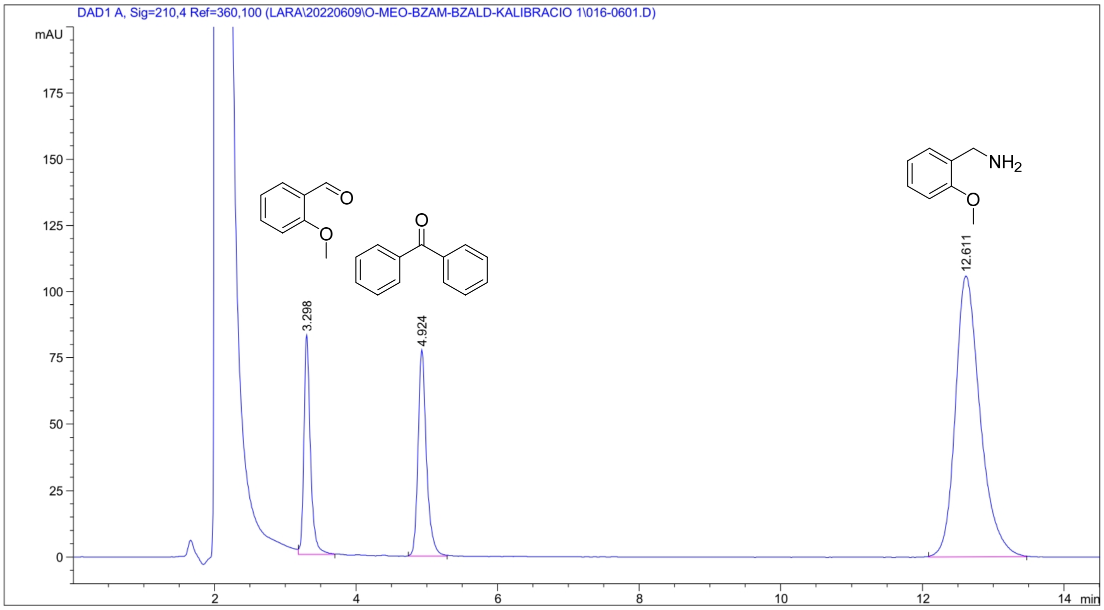

Figure S21. Elution diagram on a Crownpak CR-I(+) column for **1h** and **2h** using benzophenone as IS.

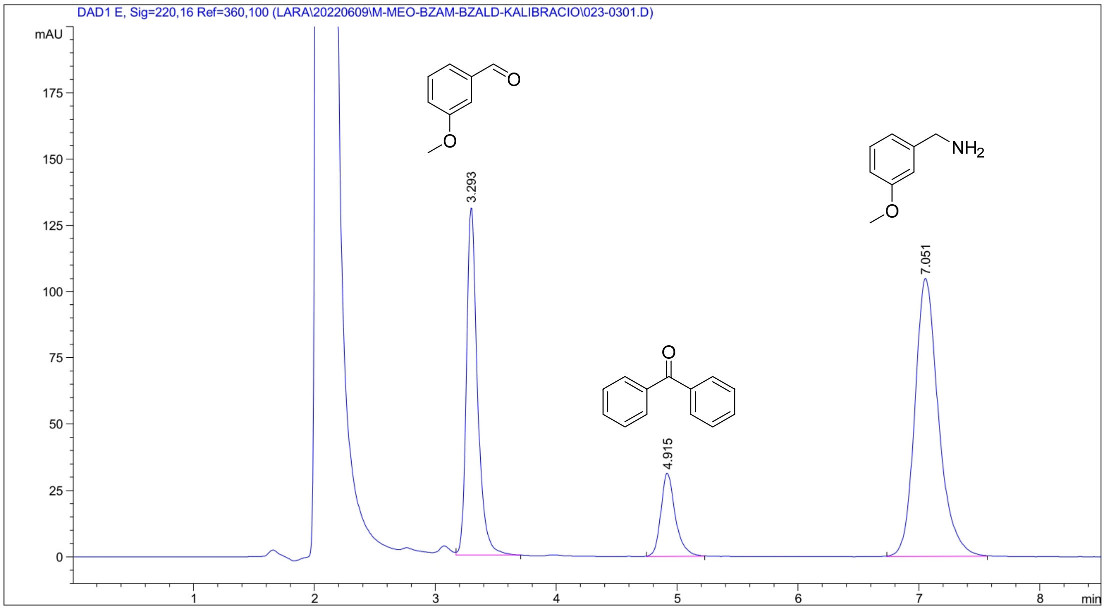

Figure S22. Elution diagram on a Crownpak CR-I(+) column for **1i** and **2i** using benzophenone as IS.

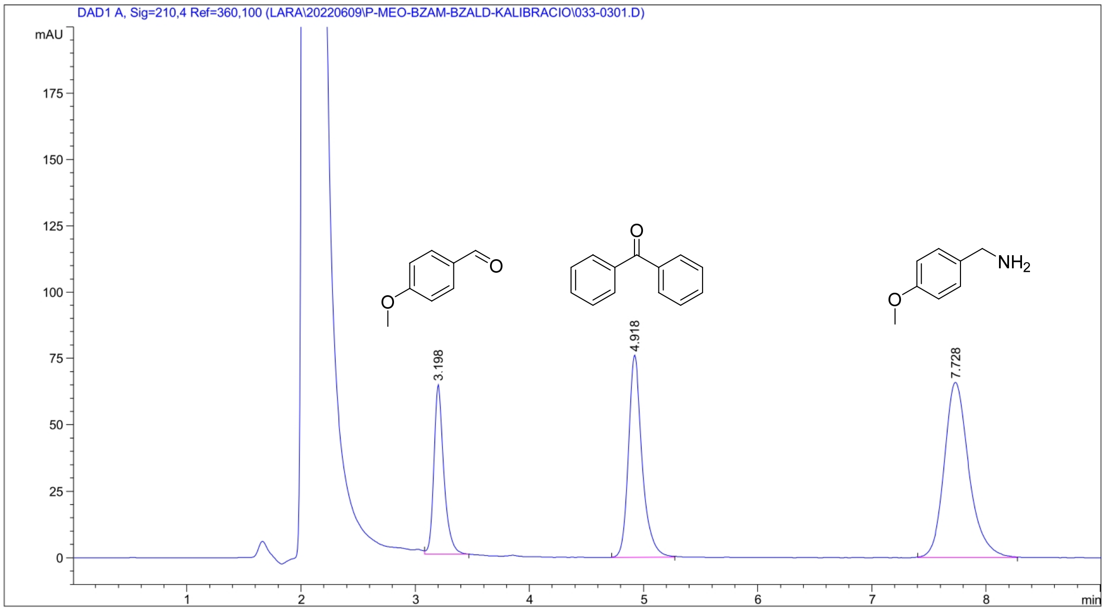

Figure S23. Elution diagram on a Crownpak CR-I(+) column for **1j** and **2j** using benzophenone as IS.

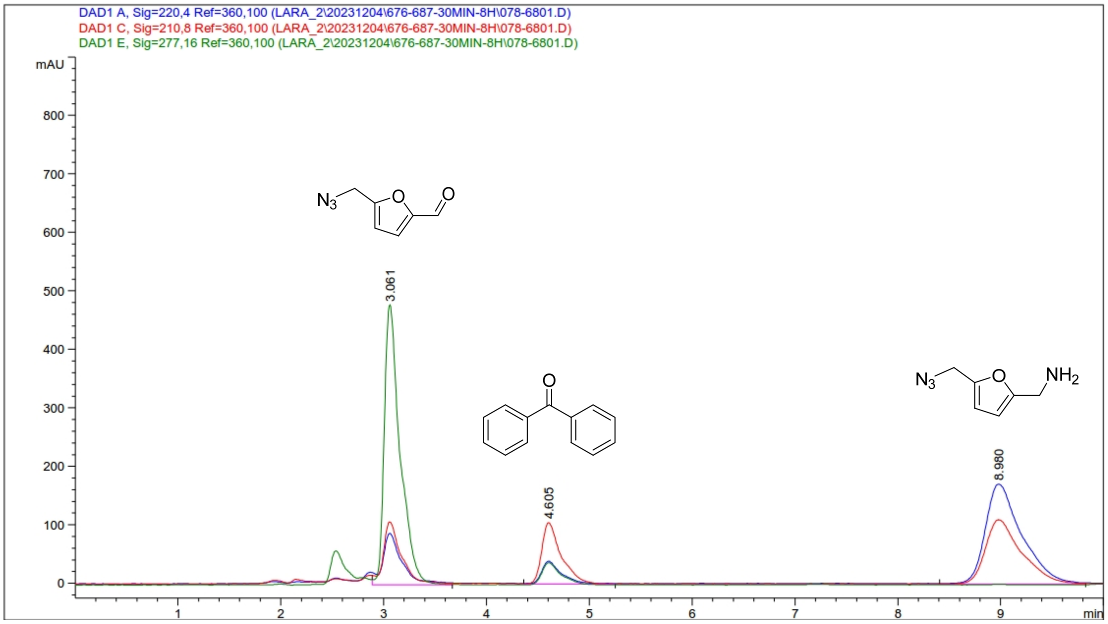

Figure S24. Elution diagram on a Crownpak CR-I(+) column for **1k** and **2k** using benzophenone as IS.

11. ^{1}H and ^{13}C NMR analyses

11.1. ^{1}H and ^{13}C NMR analyses of 2a–j

Benzylamine **(2a):**

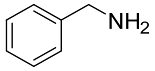

^{1}H NMR (400 MHz, CD_3CN) δ 7.37 – 7.16 (m, 5H), 3.78 (s, 2H), 1.51 (br s, 2H); ^{13}C NMR (101 MHz, CD_3CN) δ 145.37, 129.22, 127.97, 127.26, 46.92.

2-Methylbenzylamine **(2b):**

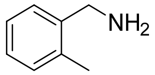

^{1}H NMR (400 MHz, CD_3CN) δ 7.35 – 7.30 (m, 1H), 7.20 – 7.11 (m, 3H), 3.77 (s, 2H), 2.30 (s, 3H), 1.55 (br s, 2H); ^{13}C NMR (101 MHz, CD_3CN) δ 142.87, 136.58, 130.81, 127.93, 127.31, 126.82, 44.50, 18.86.

3-Methylbenzylamine **(2c):**

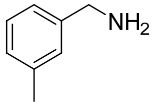

^{1}H NMR (400 MHz, CD_3CN) δ 7.24 – 6.99 (m, 4H), 3.73 (s, 2H), 2.32 (s, 3H), 1.47 (br s, 2H); ^{13}C NMR (101 MHz, CD_3CN) δ 145.34, 138.78, 129.15, 128.69, 127.92, 124.99, 46.91, 21.44.

4-Methylbenzylamine **(2d):**

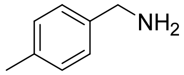

^{1}H NMR (400 MHz, CD_3CN) δ 7.20 (d, J = 8.0 Hz, 2H), 7.13 (d, J = 7.9 Hz, 2H), 3.72 (s, 2H), 2.30 (s, 3H), 1.44 (br s, 2H); ^{13}C NMR (101 MHz, CD_3CN) δ 142.37, 136.77, 129.81, 127.95, 46.67, 21.05.

2-Chlorobenzylamine **(2e):**

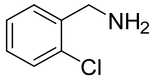

^{1}H NMR (400 MHz, CD_3CN) δ 7.52 – 7.17 (m, 4H), 3.85 (s, 2H), 1.52 (br s, 2H); ^{13}C NMR (101 MHz, CD_3CN) δ 142.29, 133.58, 130.01, 129.80, 128.90, 128.09, 44.64.

3-Chlorobenzylamine **(2f):**

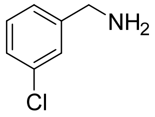

^{1}H NMR (400 MHz, CD_3CN) δ 7.40 – 7.19 (m, 4H), 3.77 (s, 2H), 1.51 (br s, 2H); ^{13}C NMR (101 MHz, CD_3CN) δ 147.90, 134.47, 130.80, 127.88, 127.16, 126.44, 46.29.

4-Chlorobenzylamine **(2g):**

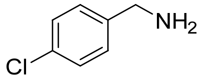

^{1}H NMR (400 MHz, CD_3CN) δ 7.31 (s, 4H), 3.76 (s, 2H), 1.50 (br s, 2H); ^{13}C NMR (101 MHz, CD_3CN) δ 144.23, 132.29, 129.65, 129.08, 46.11.

2-Methoxybenzylamine **(2h):**

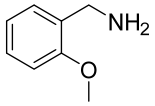

^{1}H NMR (400 MHz, CD_3CN) δ 7.30 – 7.17 (m, 2H), 6.95 – 6.85 (m, 2H), 3.81 (s, 3H), 3.71 (s, 2H), 1.46 (br s, 2H); ^{13}C NMR (101 MHz, CD_3CN) δ 158.25, 133.28, 128.93, 128.62, 121.27, 111.20, 55.84, 42.53.

3-Methoxybenzylamine **(2i):**

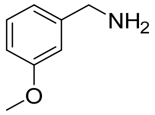

^{1}H NMR (400 MHz, CD_3CN) δ 7.26 – 7.18 (m, 1H), 6.93 – 6.86 (m, 2H), 6.81 – 6.71 (m, 1H), 3.77 (s, 3H), 3.74 (s, 2H), 1.49 (br s, 2H); ^{13}C NMR (101 MHz, CD_3CN) δ 160.76, 147.19, 130.25, 120.15, 113.43, 112.60, 55.69, 46.89.

4-Methoxybenzylamine **(2j):**

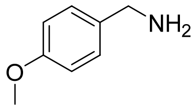

^{1}H NMR (400 MHz, CD_3CN) δ 7.23 (d, J = 8.7 Hz, 2H), 6.87 (d, J = 8.6 Hz, 2H), 3.76 (s, 3H), 3.70 (s, 2H), 1.47 (br s, 2H); ^{13}C NMR (101 MHz, CD_3CN) δ 159.27, 137.46, 129.15, 114.53, 55.77, 46.32.

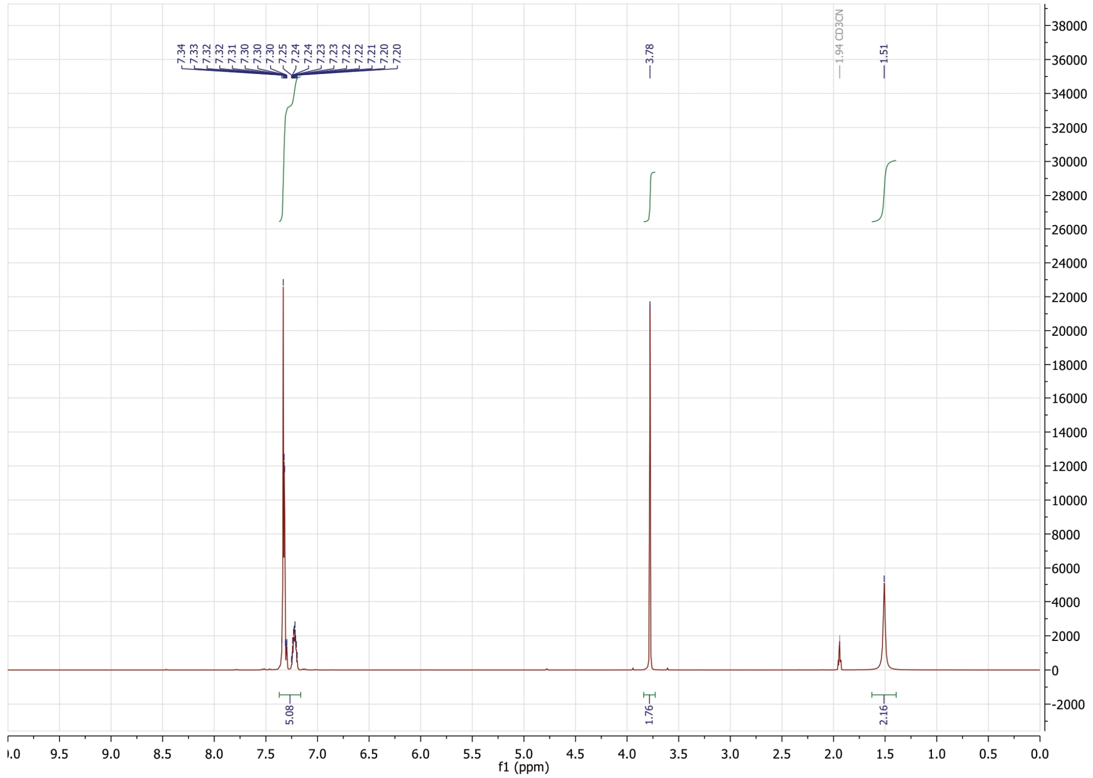

Figure S25. ^{1}H NMR spectrum of **2a** (400 MHz, CD_3CN).

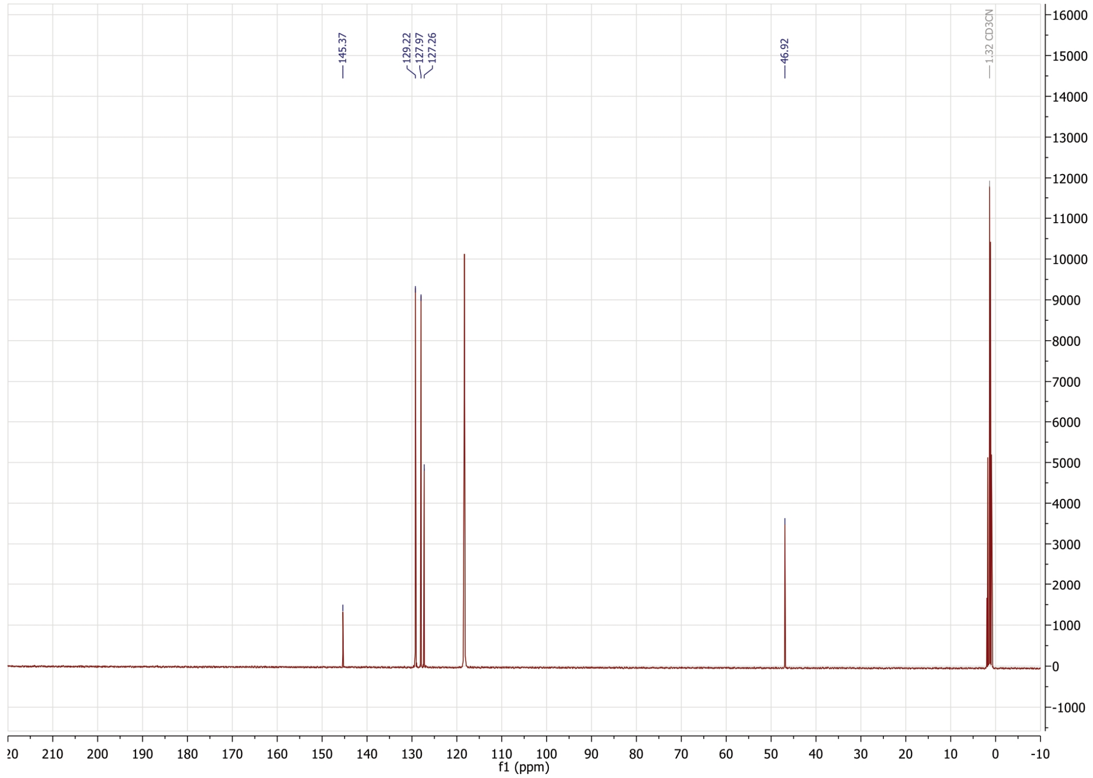

Figure S26. ^{13}C NMR spectrum of **2a** (101 MHz, CD_3CN).

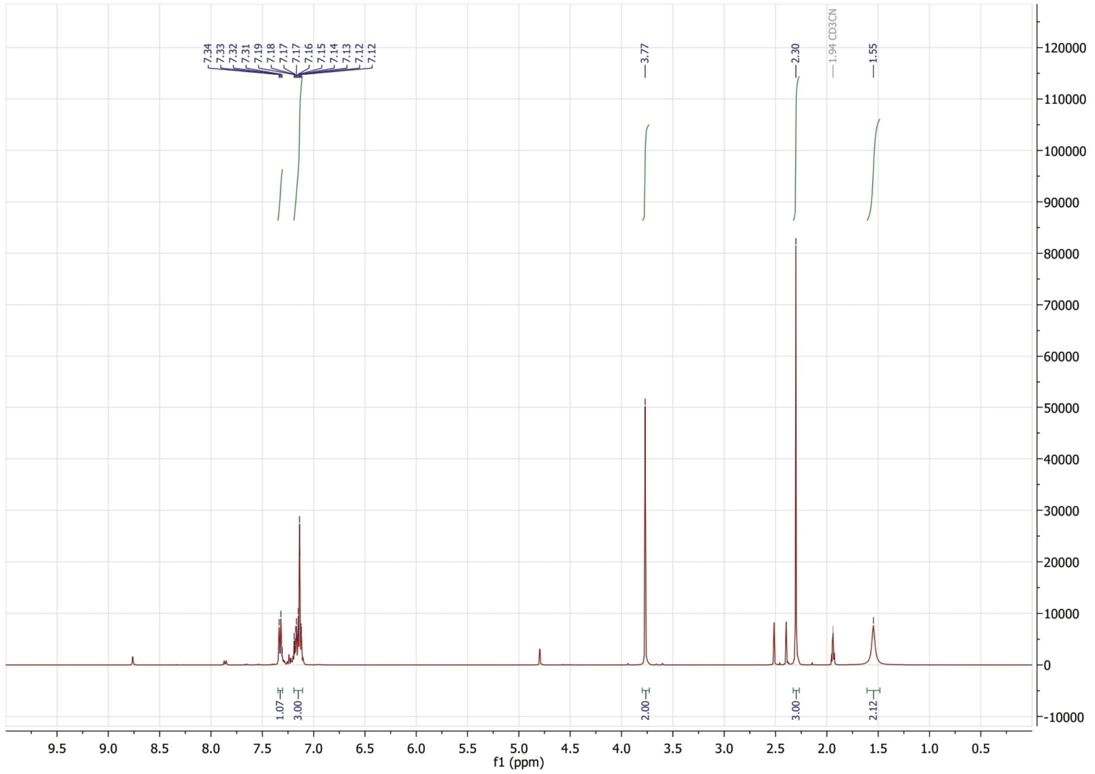

Figure S27. ^{1}H NMR spectrum of **2b** (400 MHz, CD_3CN).

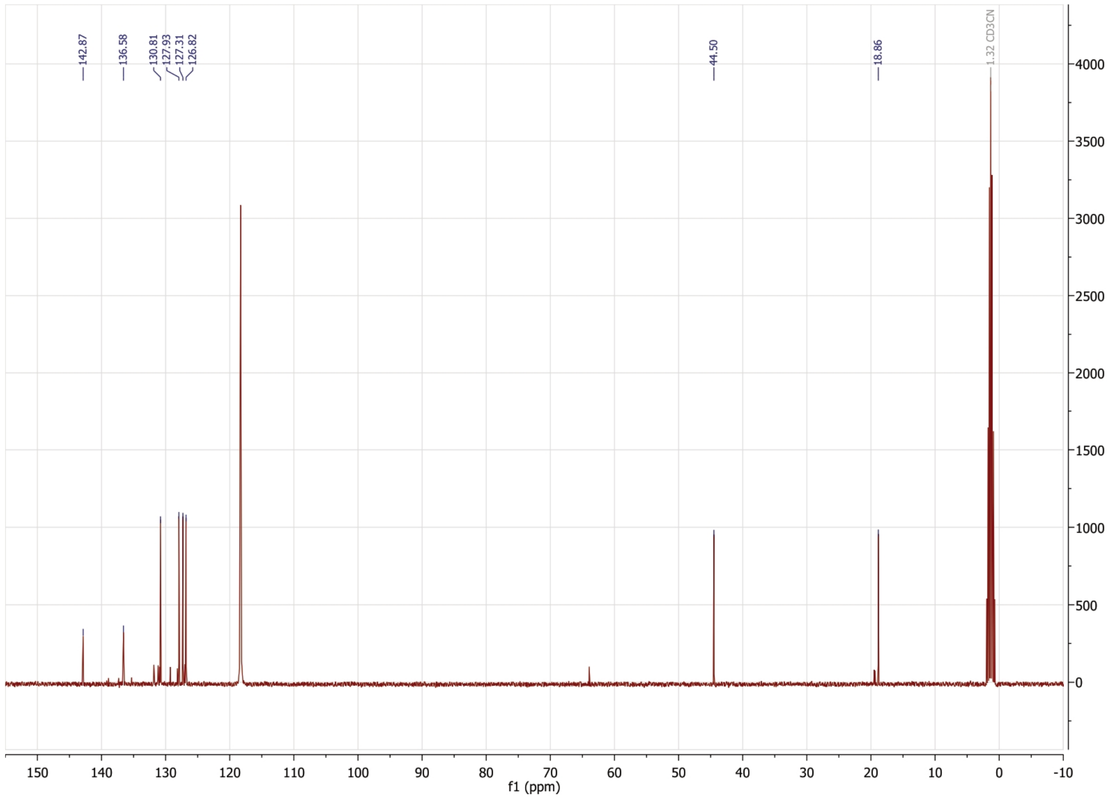

Figure S28. ^{13}C NMR spectrum of **2b** (101 MHz, CD_3CN).

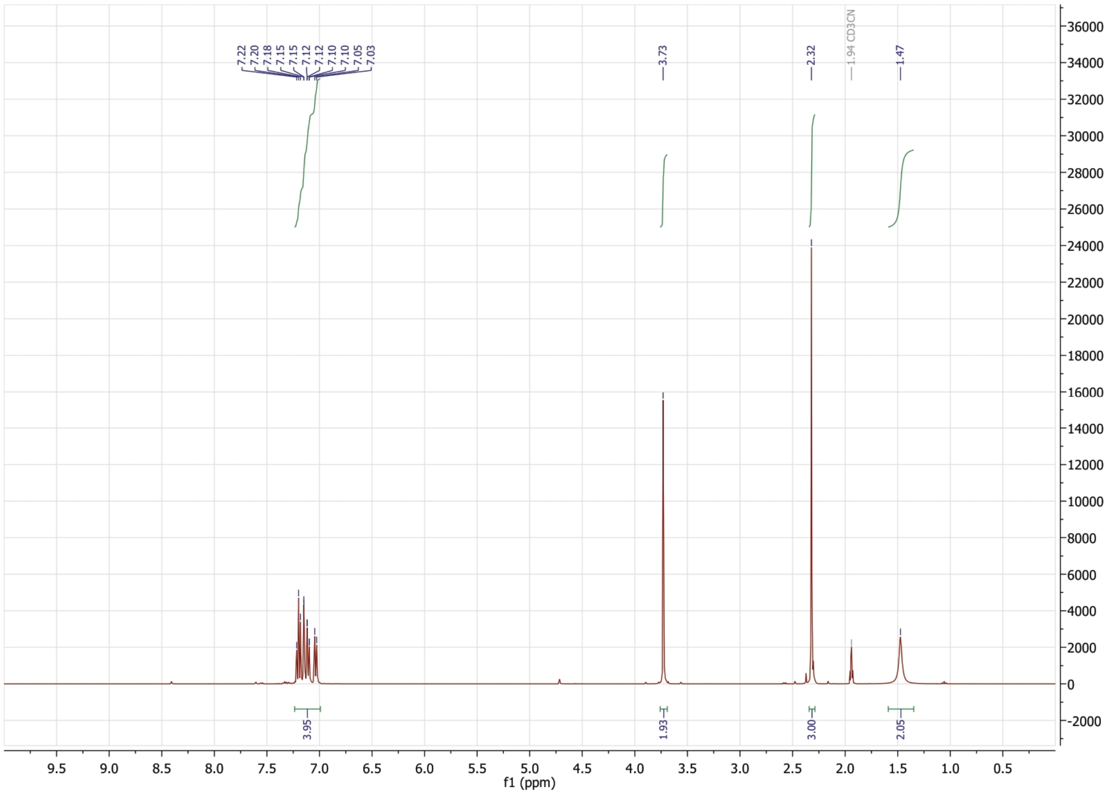

Figure S29. ^{1}H NMR spectrum of **2c** (400 MHz, CD_3CN).

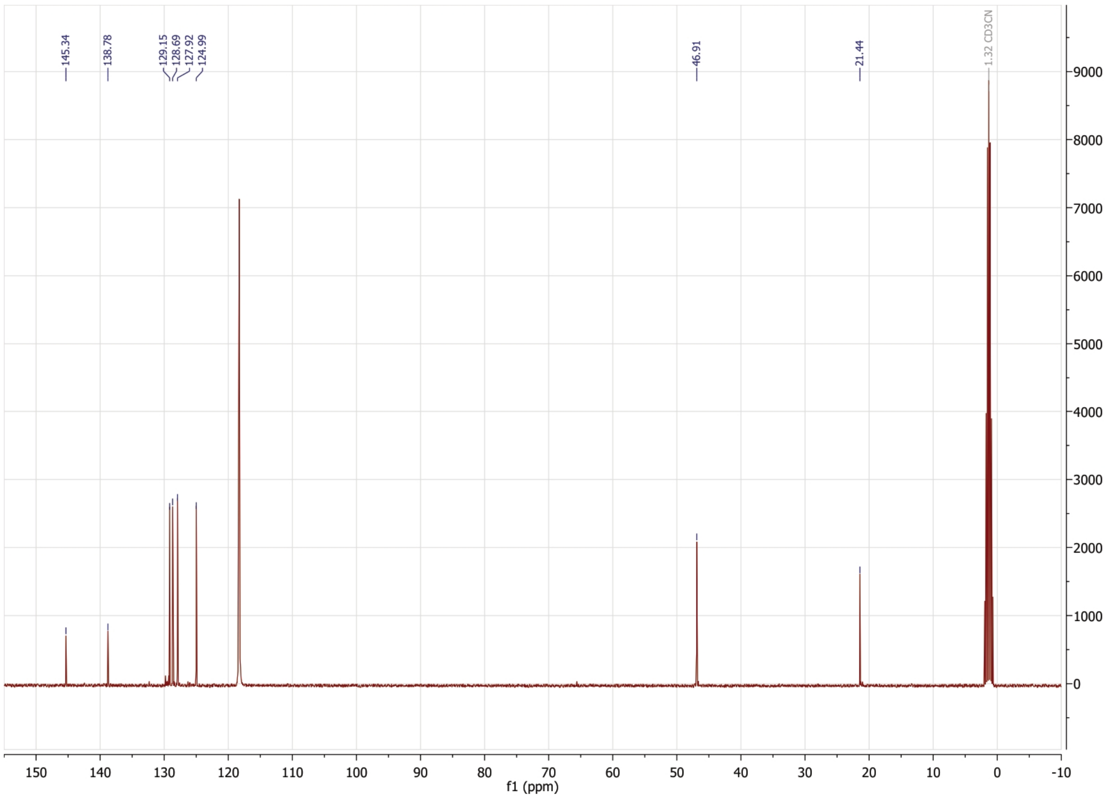

Figure S30. ^{13}C NMR spectrum of **2c** (101 MHz, CD_3CN).

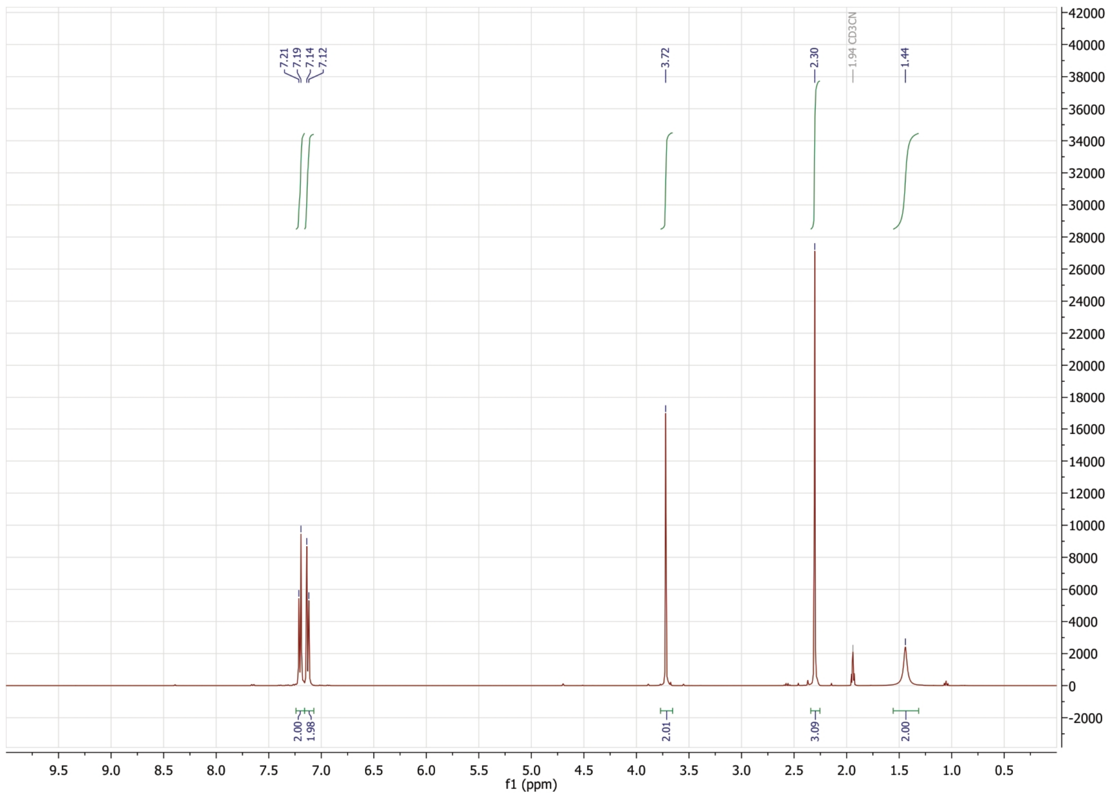

Figure S31. ^{1}H NMR spectrum of **2d** (400 MHz, CD_3CN).

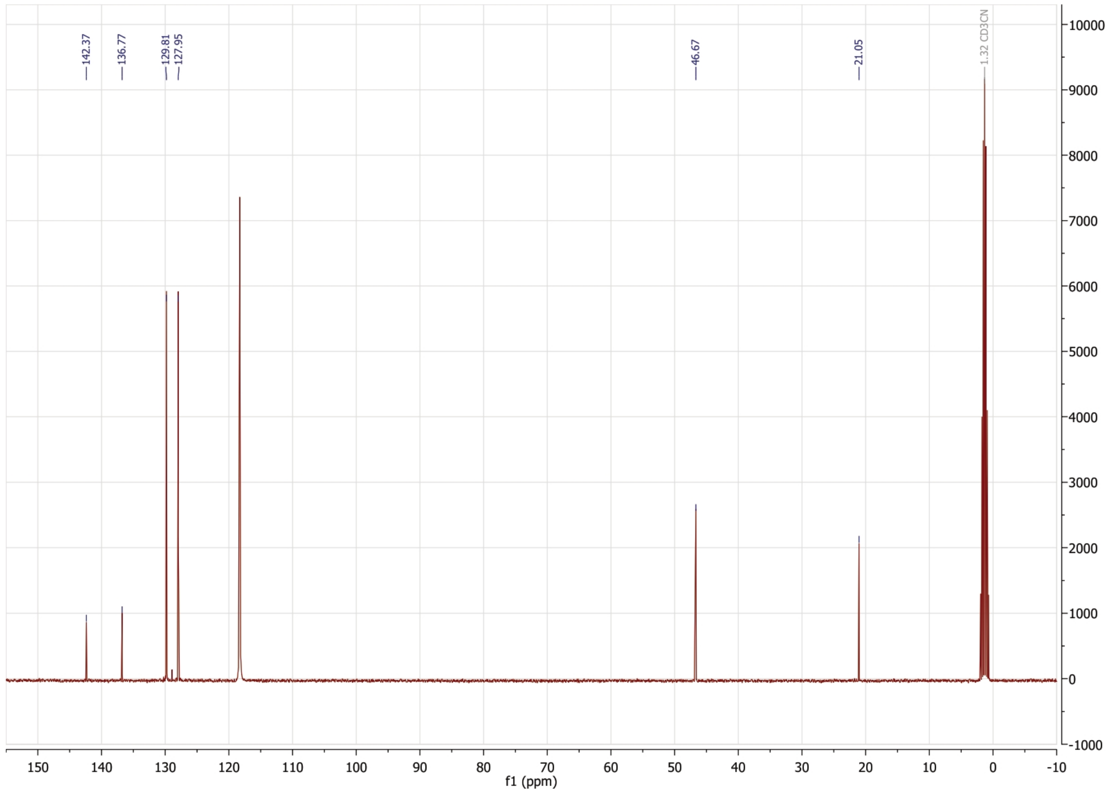

Figure S32. ^{13}C NMR spectrum of **2d** (101 MHz, CD_3CN).

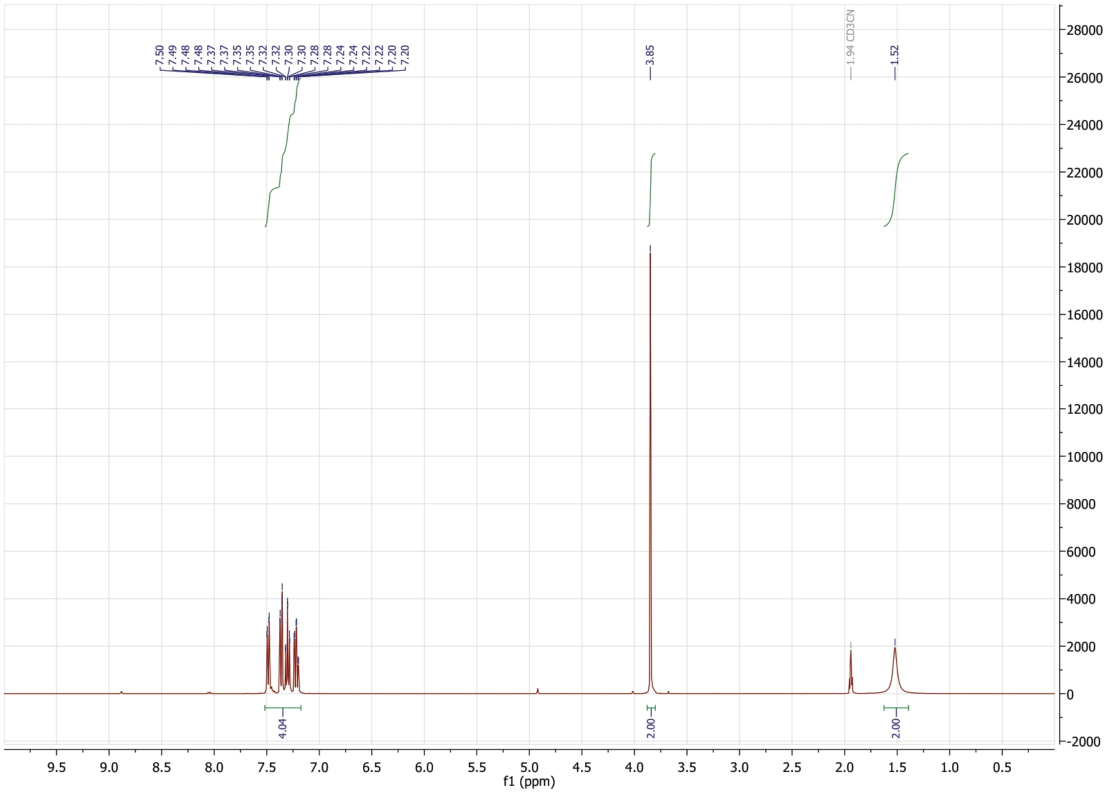

Figure S33. ^{1}H NMR spectrum of **2e** (400 MHz, CD_3CN).

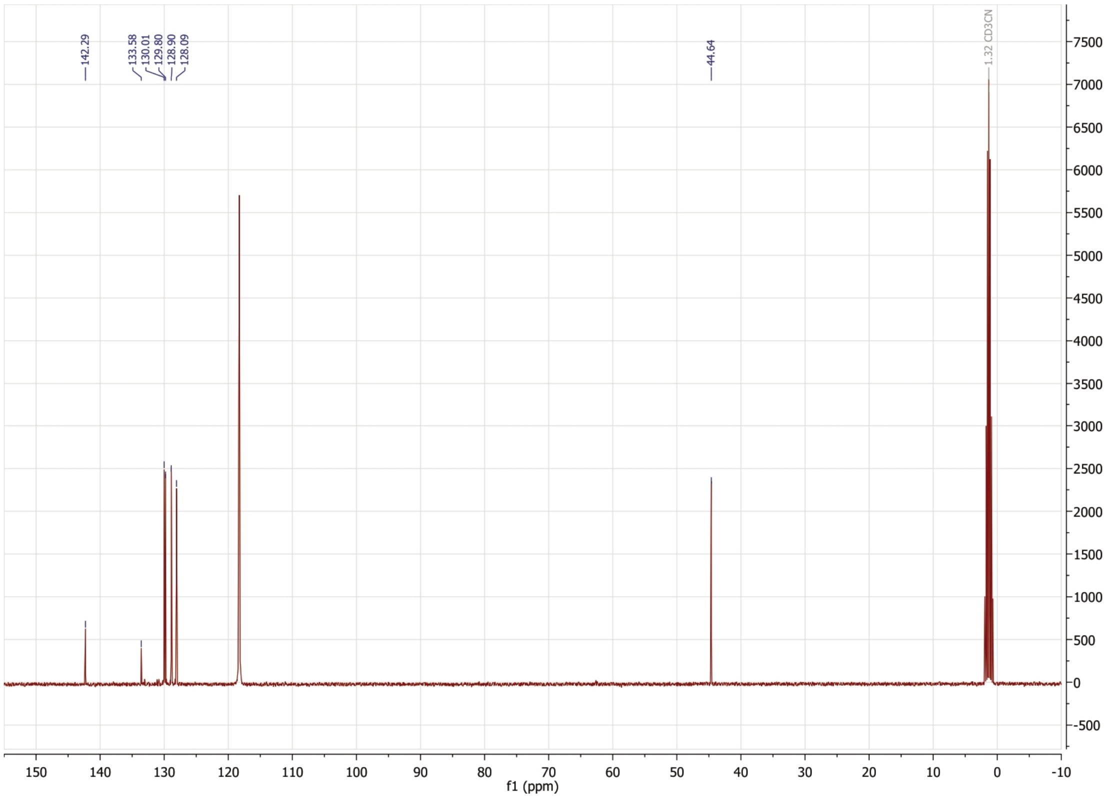

Figure S34. ^{13}C NMR spectrum of **2e** (101 MHz, CD_3CN).

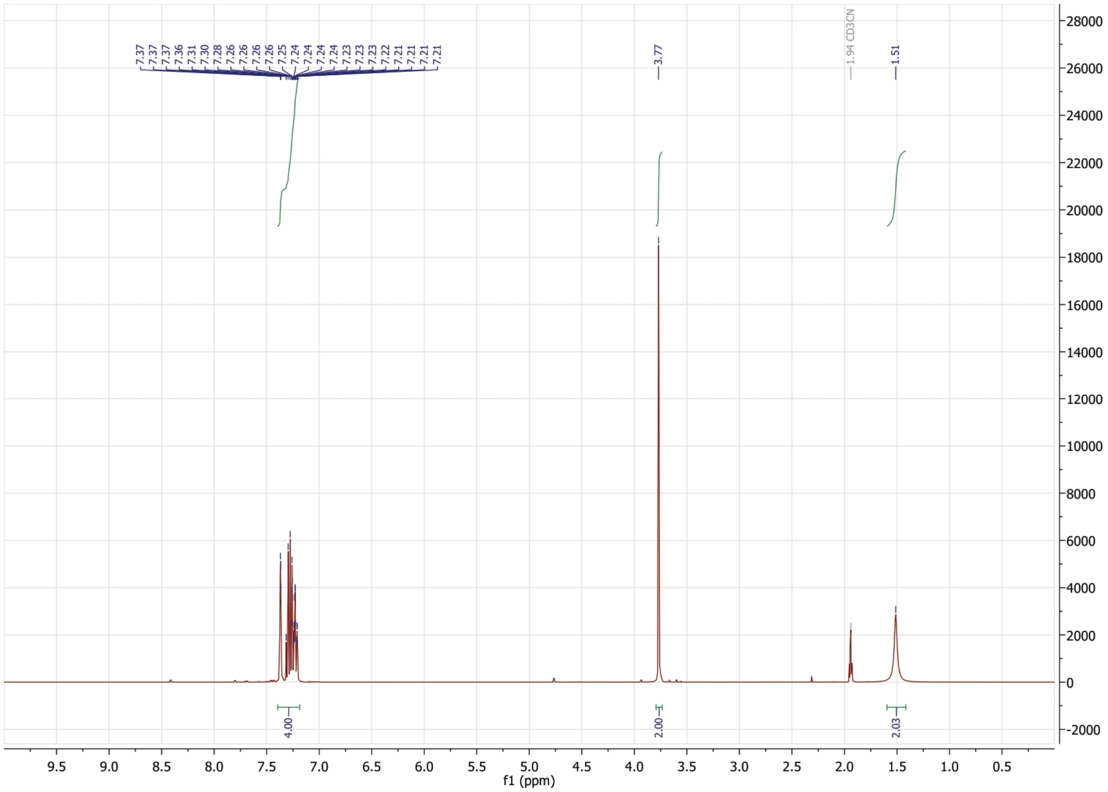

Figure S35. ^{1}H NMR spectrum of **2f** (400 MHz, CD_3CN).

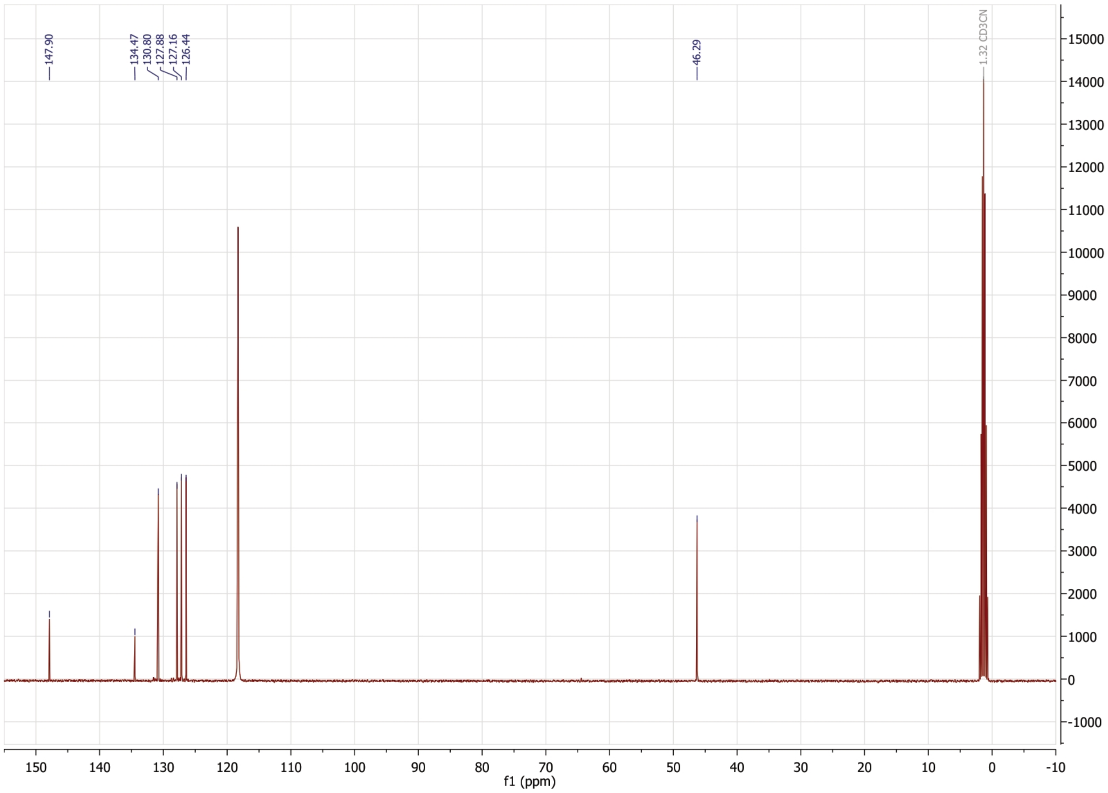

Figure S36. ^{13}C NMR spectrum of **2f** (101 MHz, CD_3CN).

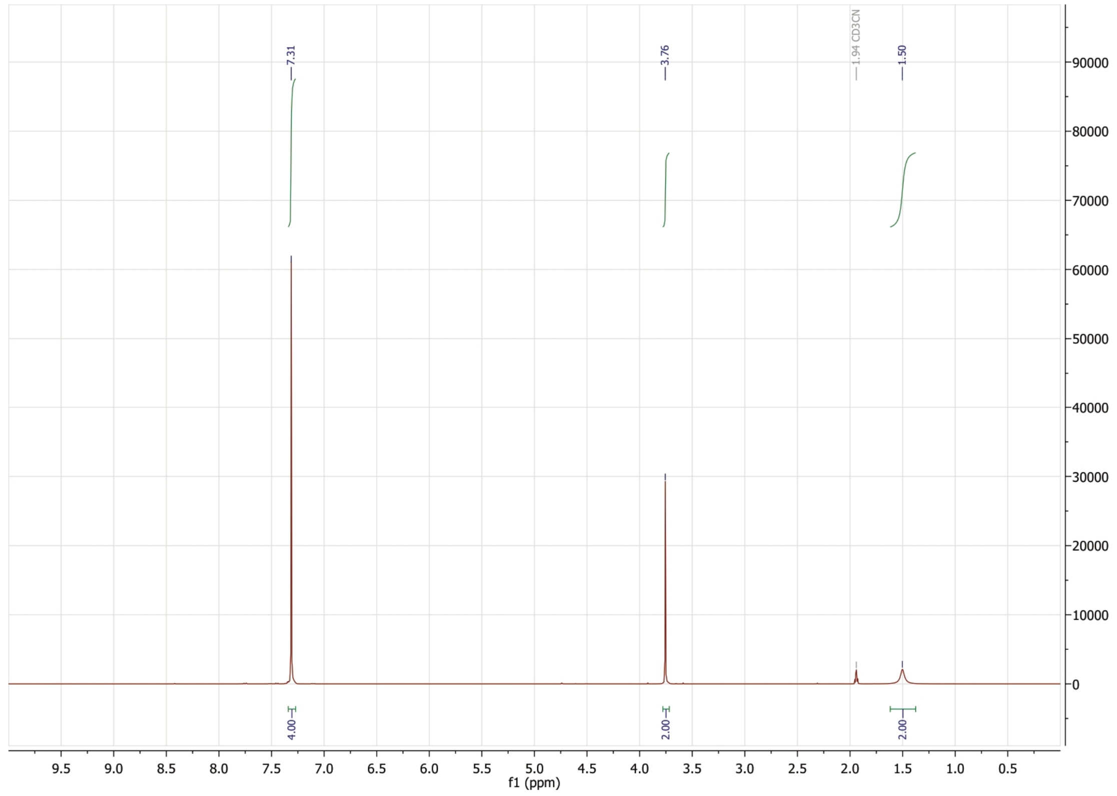

Figure S37. ^{1}H NMR spectrum of **2g** (400 MHz, CD_3CN).

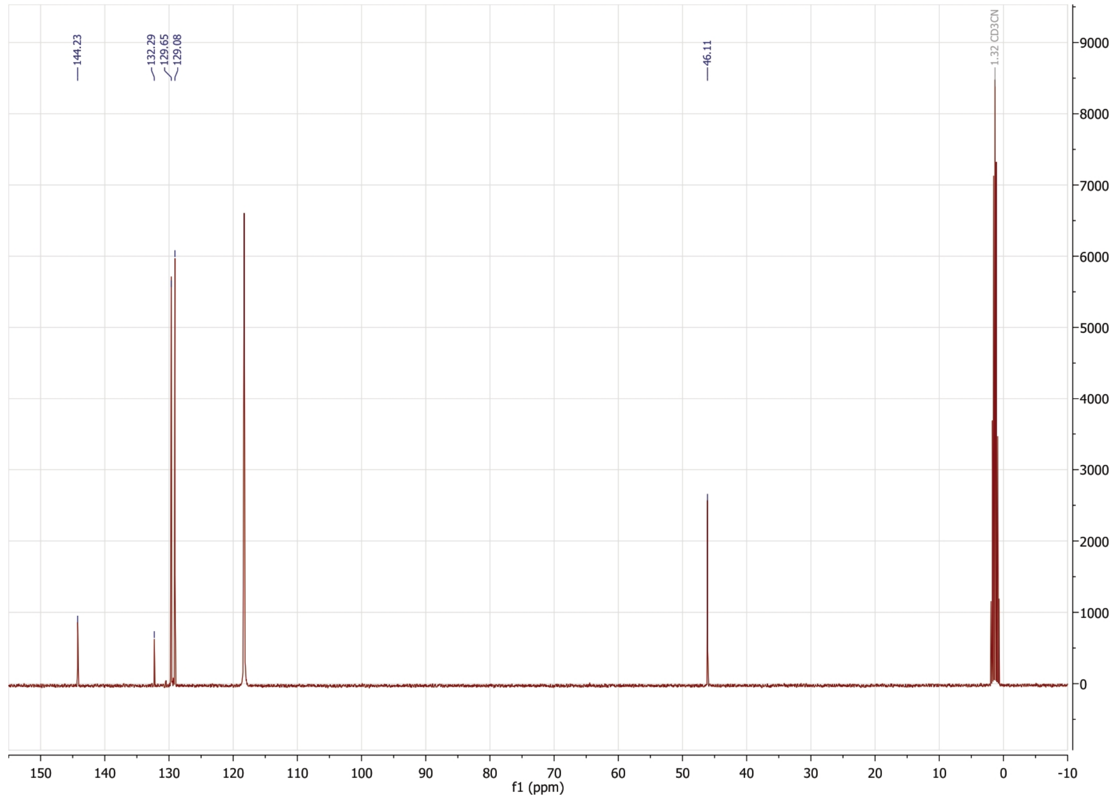

Figure S38. ^{13}C NMR spectrum of **2g** (101 MHz, CD_3CN).

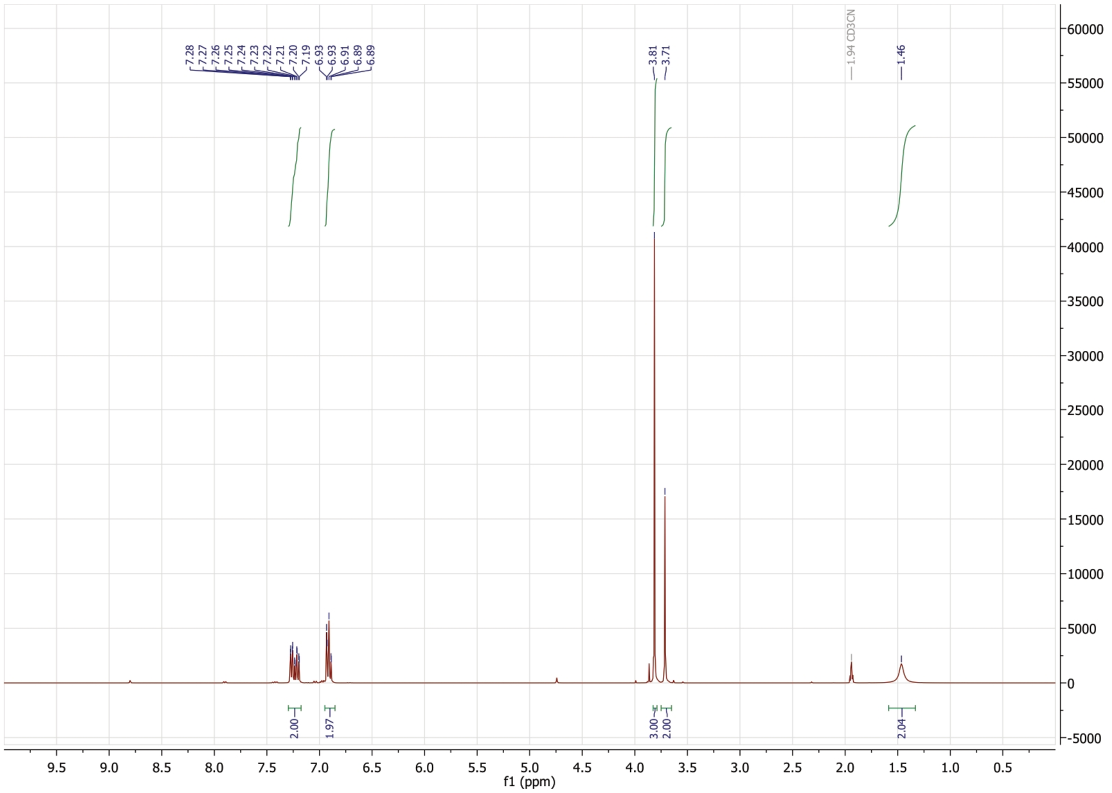

Figure S39. ^{1}H NMR spectrum of **2h** (400 MHz, CD_3CN).

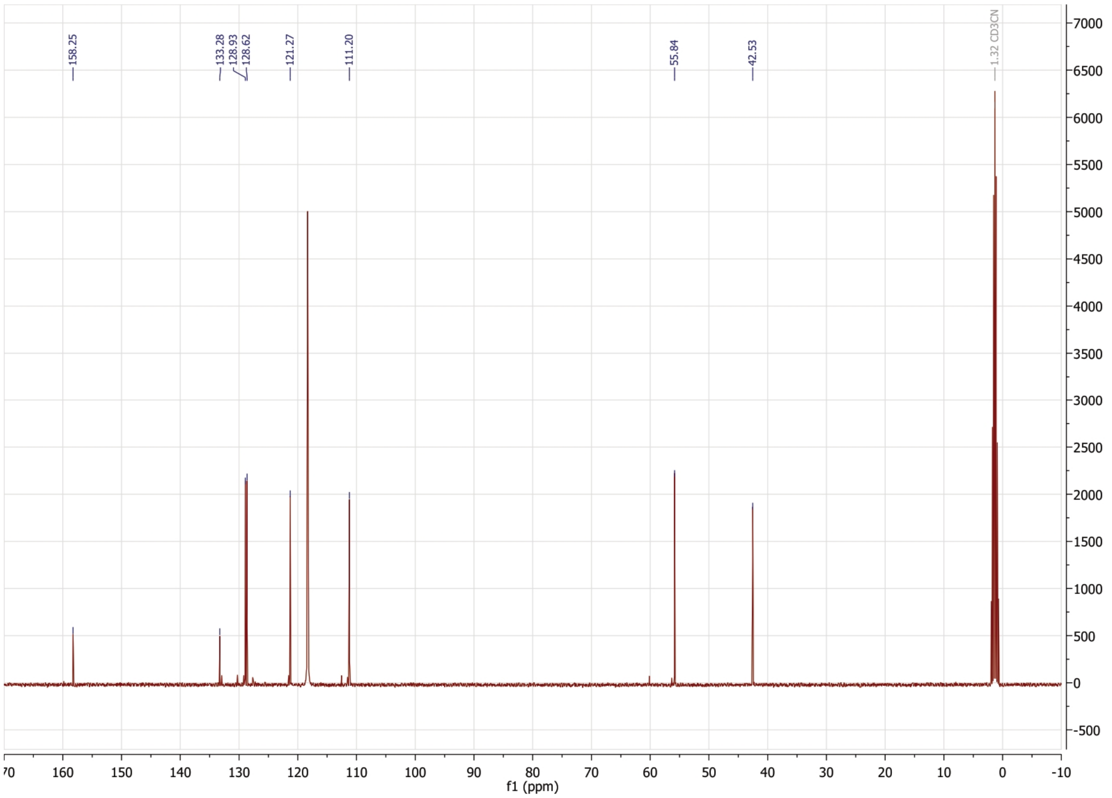

Figure S40. ^{13}C NMR spectrum of **2h** (101 MHz, CD_3CN).

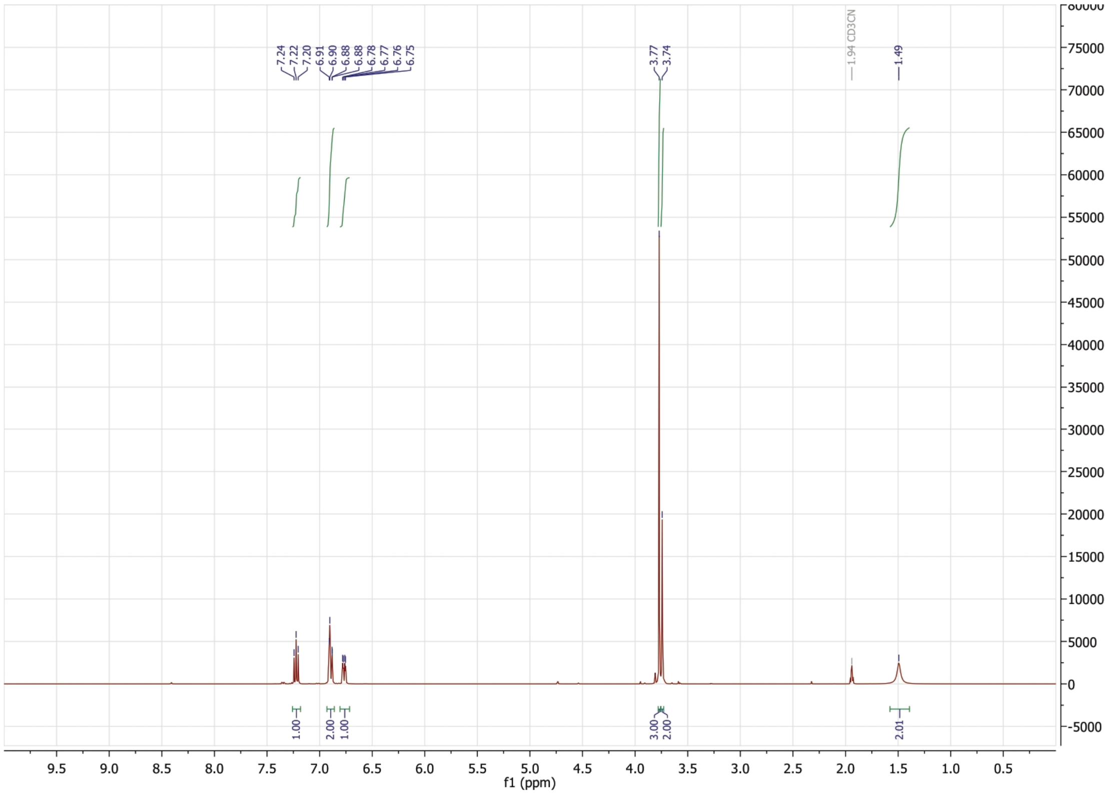

Figure S41. ^{1}H NMR spectrum of **2i** (400 MHz, CD_3CN).

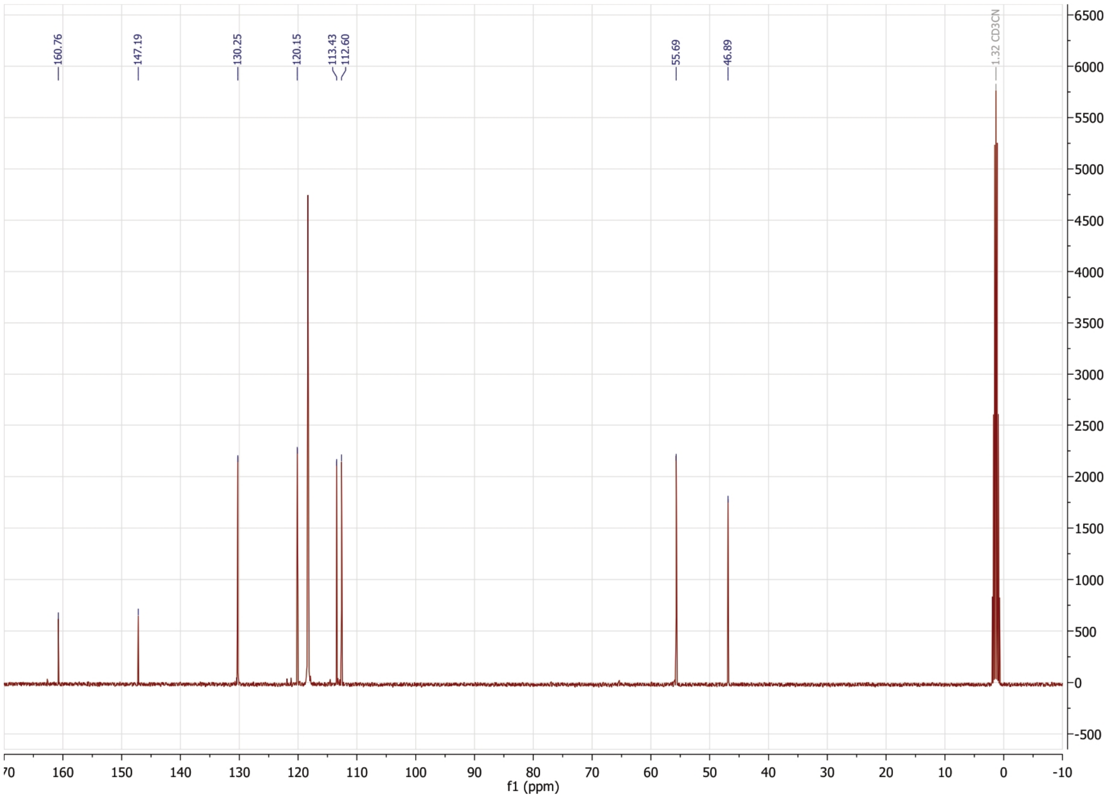

Figure S42. ^{13}C NMR spectrum of **2i** (101 MHz, CD_3CN).

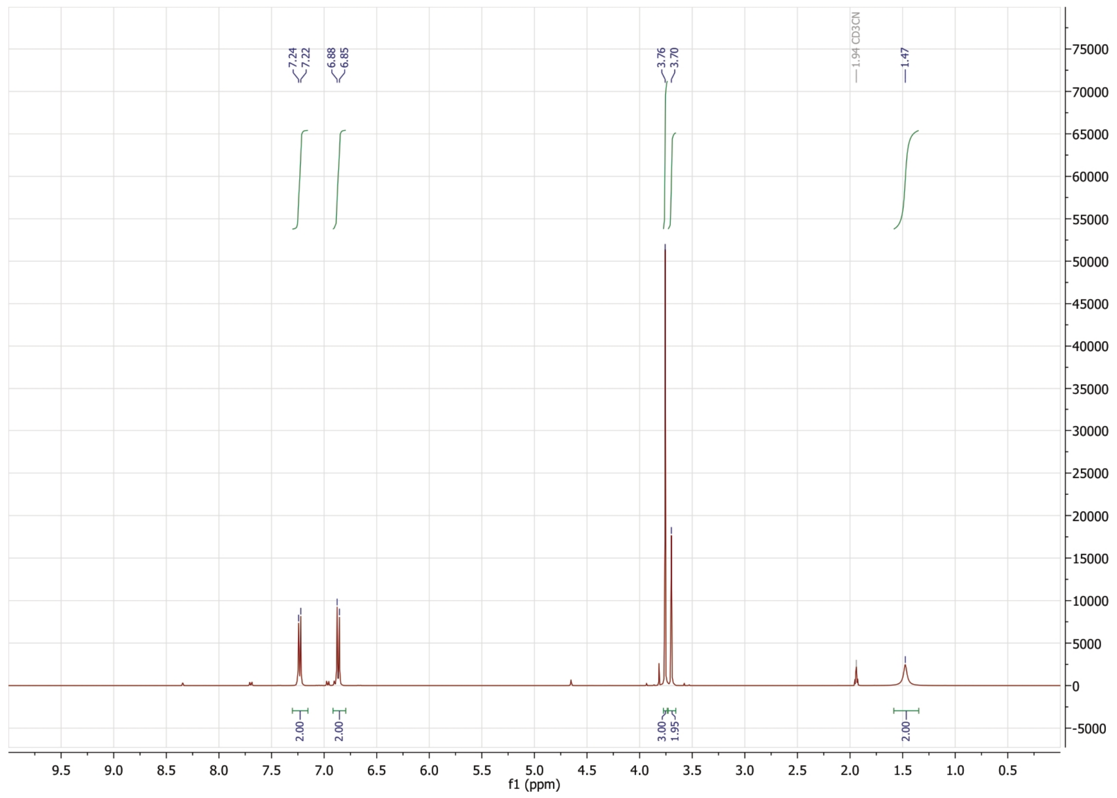

Figure S43. ^{1}H NMR spectrum of **2j** (400 MHz, CD_3CN).

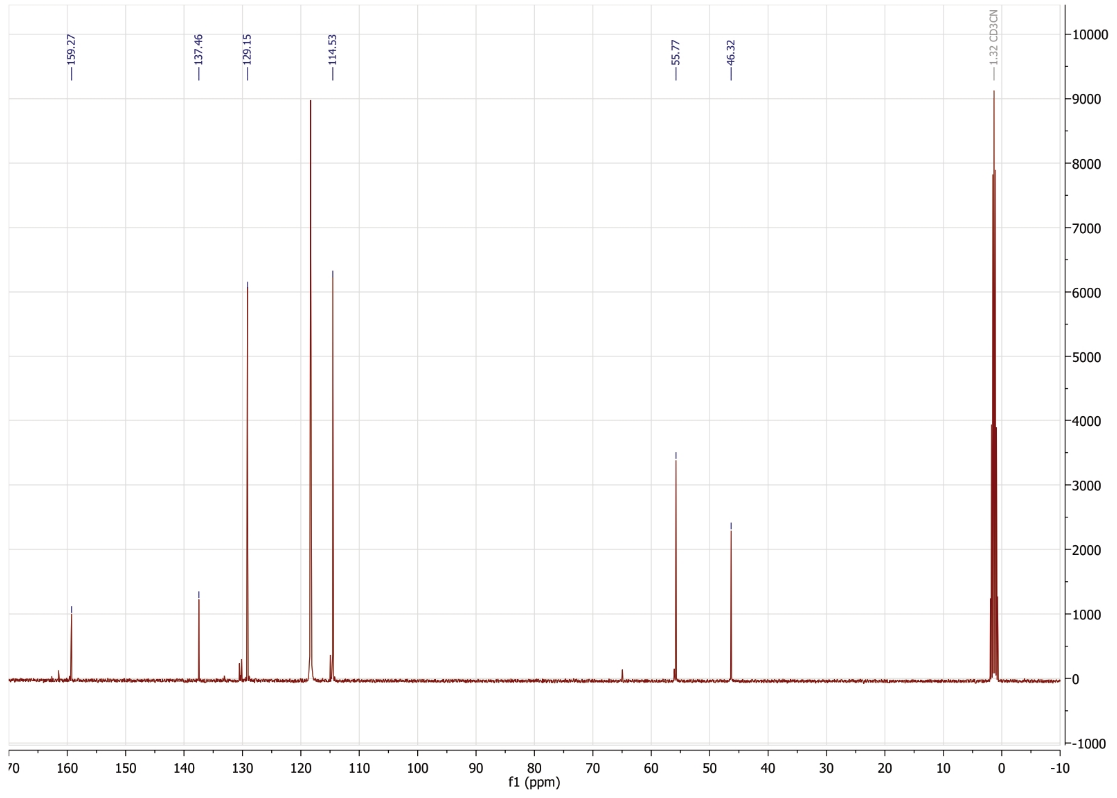

Figure S44. ^{13}C NMR spectrum of **2j** (101 MHz, CD_3CN).

11.2. ^{1}H and ^{13}C NMR analyses of 3a–j

Rac-1-hydroxy-1-phenylpropan-2-one (*rac*-**3a**):

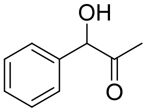

^{1}H NMR (400 MHz, $CDCl_3$) δ 7.44 – 7.28 (m, 5H), 5.09 (s, 1H), 4.31 (br s, 1H), 2.08 (s, 3H); ^{13}C NMR (101 MHz, $CDCl_3$) δ 207.21, 138.04, 129.16, 128.89, 127.49, 80.27, 25.41.

Rac-1-hydroxy-1-(2-methylphenyl)propan-2-one (*rac*-**3b**):

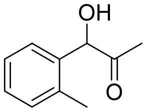

^{1}H NMR (600 MHz, $CDCl_3$) δ 7.26 – 7.18 (m, 3H), 7.18 – 7.10 (m, 1H), 5.26 (s, 1H), 4.19 (br s, 1H), 2.40 (s, 3H), 2.05 (s, 3H); ^{13}C NMR (151 MHz, $CDCl_3$) δ 207.75, 136.56, 136.02, 131.46, 128.83, 128.43, 126.75, 78.20, 25.51, 19.43.

Rac-1-hydroxy-1-(3-methylphenyl)propan-2-one (*rac*-**3c**):

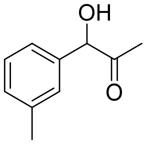

^{1}H NMR (600 MHz, $CDCl_3$) δ 7.30 – 7.24 (m, 1H), 7.19 – 7.09 (m, 3H), 5.05 (s, 1H), 4.29 (br s, 1H), 2.35 (s, 3H), 2.08 (s, 3H); ^{13}C NMR (151 MHz, $CDCl_3$) δ 207.34, 138.94, 137.97, 129.62, 128.99, 128.00, 124.68, 80.24, 25.40, 21.49.

Rac-1-hydroxy-1-(4-methylphenyl)propan-2-one (*rac*-**3d**):

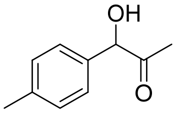

^{1}H NMR (600 MHz, $CDCl_3$) δ 7.22 – 7.17 (m, 4H), 5.06 (d, J = 3.7 Hz, 1H), 4.26 (d, J = 4.3 Hz, 1H), 2.35 (s, 3H), 2.07 (s, 3H); ^{13}C NMR (151 MHz, $CDCl_3$) δ 207.42, 138.73, 135.12, 129.81, 127.40, 80.05, 25.37, 21.31.

Rac-1-(2-chlorophenyl)-1-hydroxypropan-2-one (*rac*-**3e**):

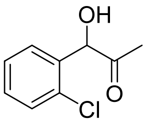

^{1}H NMR (400 MHz, $CDCl_3$) δ 7.46 – 7.38 (m, 1H), 7.32 – 7.26 (m, 3H), 5.59 (d, J = 4.0 Hz, 1H), 4.37 (d, J = 4.2 Hz, 1H), 2.13 (s, 3H); ^{13}C NMR (101 MHz, $CDCl_3$) δ 206.43, 135.76, 133.53, 130.21, 130.03, 129.03, 127.69, 76.59, 25.42.

Rac-1-(3-chlorophenyl)-1-hydroxypropan-2-one (*rac*-**3f**):

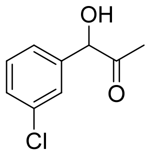

^{1}H NMR (400 MHz, $CDCl_3$) δ 7.36 – 7.29 (m, 3H), 7.25 – 7.20 (m, 1H), 5.06 (s, 1H), 4.32 (br s, 1H), 2.11 (s, 3H); ^{13}C NMR (101 MHz, $CDCl_3$) δ 206.40, 140.00, 135.11, 130.42, 129.10, 127.58, 125.64, 79.62, 25.39.

Rac-1-(4-chlorophenyl)-1-hydroxypropan-2-one (*rac*-**3g**):

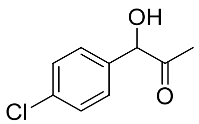

^{1}H NMR (600 MHz, $CDCl_3$) δ 7.40 – 7.34 (m, 2H), 7.29 – 7.26 (m, 2H), 5.07 (d, *J* = 3.4 Hz, 1H), 4.31 (d, *J* = 4.1 Hz, 1H), 2.08 (s, 3H); ^{13}C NMR (151 MHz, $CDCl_3$) δ 206.63, 136.56, 134.82, 129.35, 128.79, 79.54, 25.33.

Rac-1-hydroxy-1-(2-methoxyphenyl)propan-2-one (*rac*-**3h**):

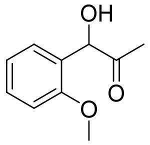

^{1}H NMR (600 MHz, $CDCl_3$) δ 7.34 – 7.29 (m, 1H), 7.26 – 7.23 (m, 1H), 7.00 – 6.95 (m, 1H), 6.94 – 6.89 (m, 1H), 5.37 (s, 1H), 4.21 (br s, 1H), 3.85 (s, 3H), 2.07 (s, 3H); ^{13}C NMR (151 MHz, $CDCl_3$) δ 207.55, 156.96, 130.03, 129.20, 126.56, 121.23, 111.19, 75.40, 55.60, 25.11.

Rac-1-hydroxy-1-(3-methoxyphenyl)propan-2-one (*rac*-**3i**):

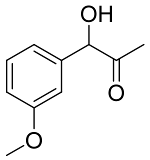

^{1}H NMR (400 MHz, $CDCl_3$) δ 7.33 – 7.27 (m, 1H), 6.95 – 6.81 (m, 3H), 5.06 (d, *J* = 3.4 Hz, 1H), 4.31 (d, *J* = 4.1 Hz, 1H), 3.80 (s, 3H), 2.09 (s, 3H); ^{13}C NMR (101 MHz, $CDCl_3$) δ 207.12, 160.18, 139.52, 130.16, 119.88, 114.47, 112.65, 80.12, 55.39, 25.35.

Rac-1-hydroxy-1-(4-methoxyphenyl)propan-2-one (*rac*-**3j**):

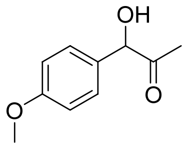

^{1}H NMR (600 MHz, $CDCl_3$) δ 7.25 – 7.21 (m, 2H), 6.94 – 6.89 (m, 2H), 5.04 (s, 1H), 4.25 (br s, 1H), 3.81 (s, 3H), 2.07 (s, 3H); ^{13}C NMR (151 MHz, $CDCl_3$) δ 207.52, 160.06, 130.15, 128.77, 114.57, 79.70, 55.46, 25.39.

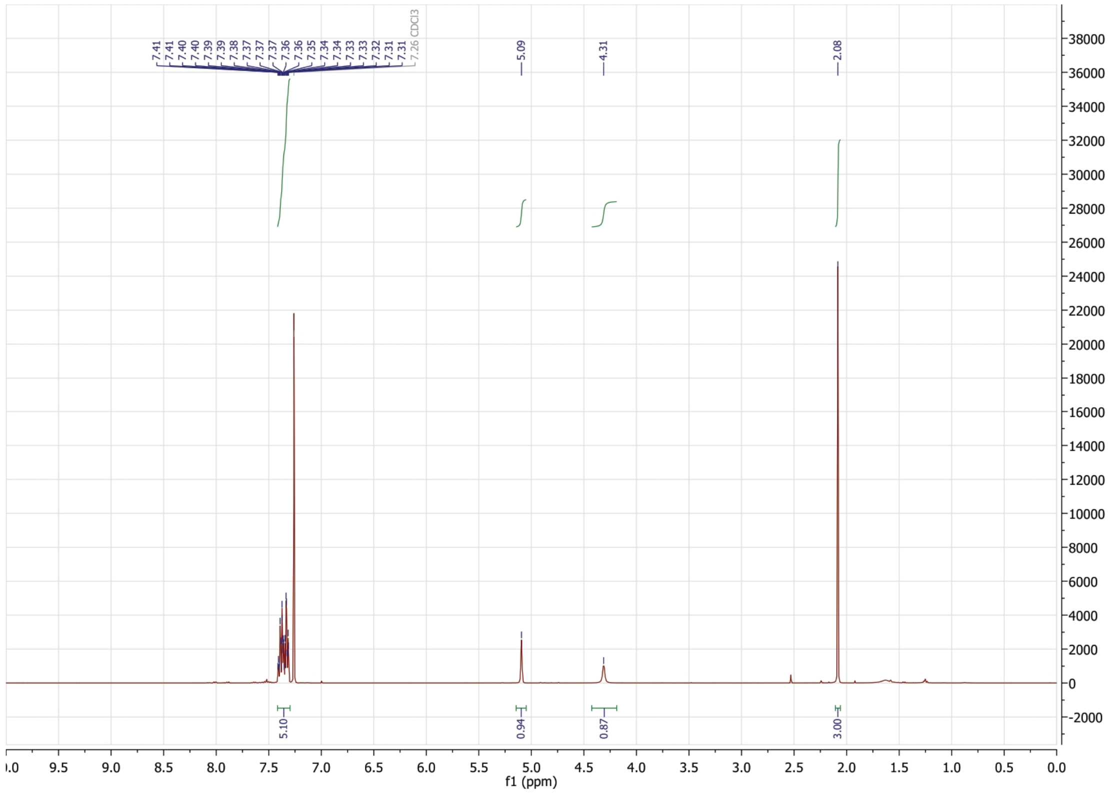

Figure S45. ^{1}H NMR spectrum of **3a** (400 MHz, $CDCl_3$).

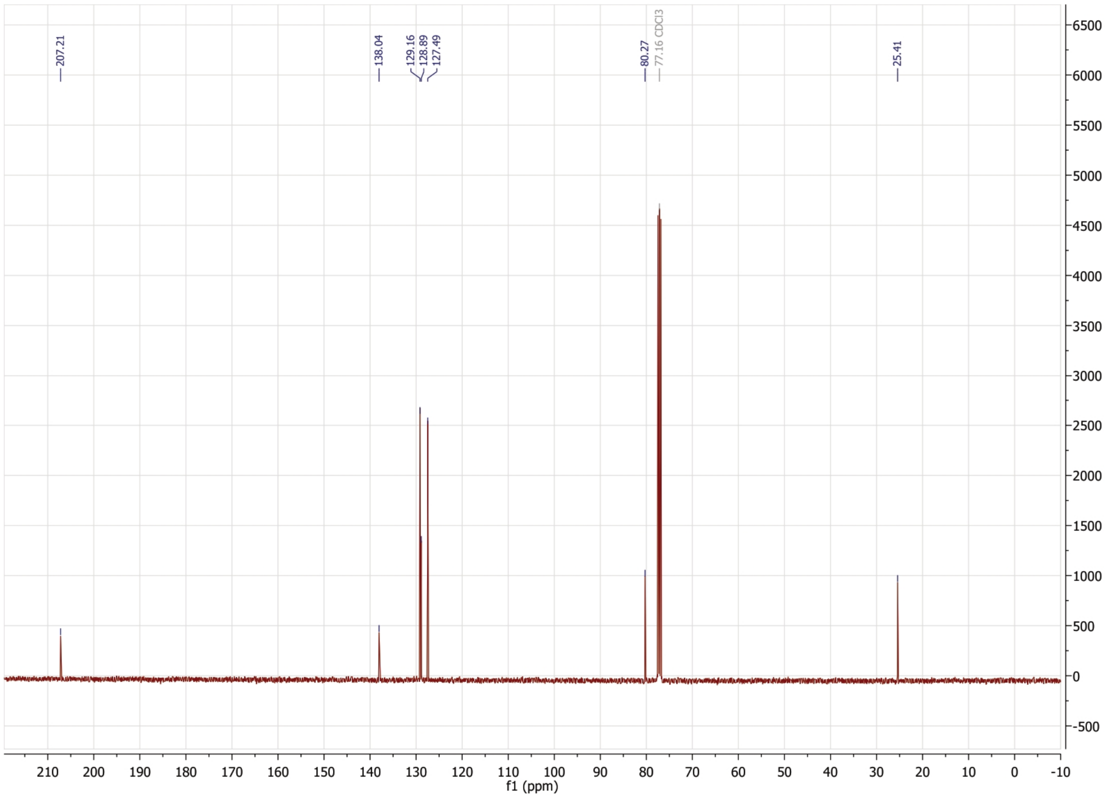

Figure S46. ^{13}C NMR spectrum of **3a** (101 MHz, $CDCl_3$).

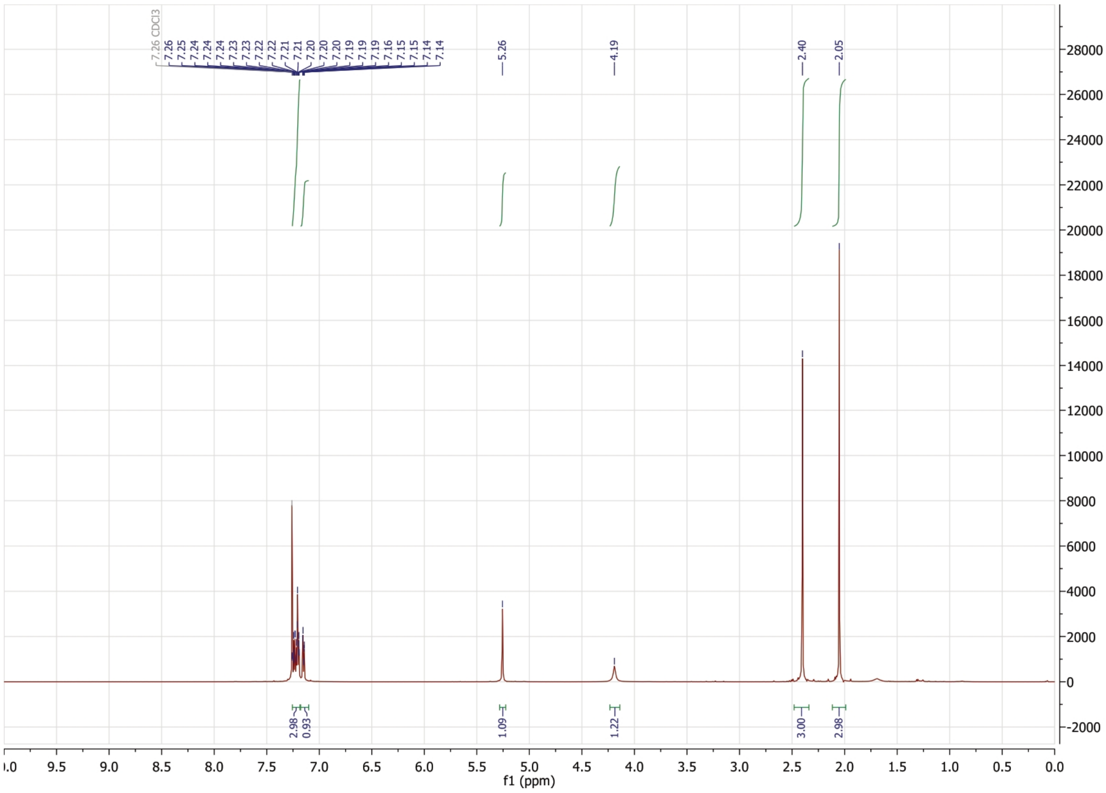

Figure S47. ^{1}H NMR spectrum of **3b** (600 MHz, $CDCl_3$).

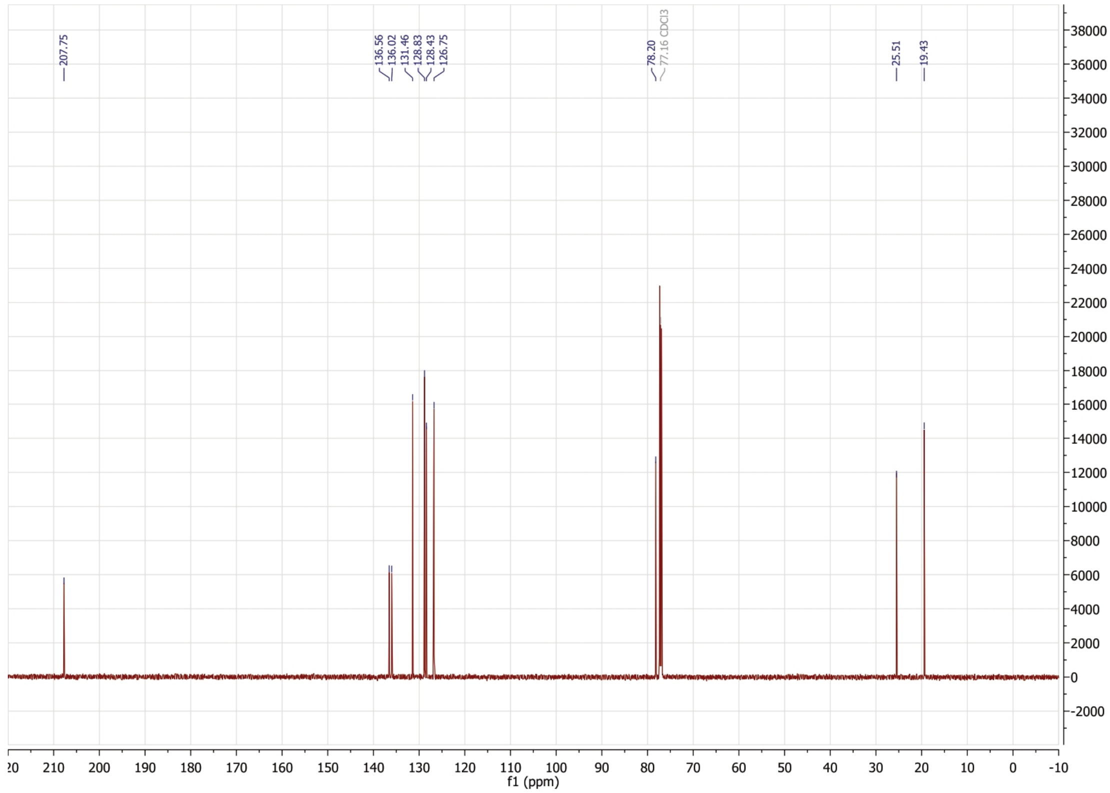

Figure S48. ^{13}C NMR spectrum of **3b** (151 MHz, $CDCl_3$).

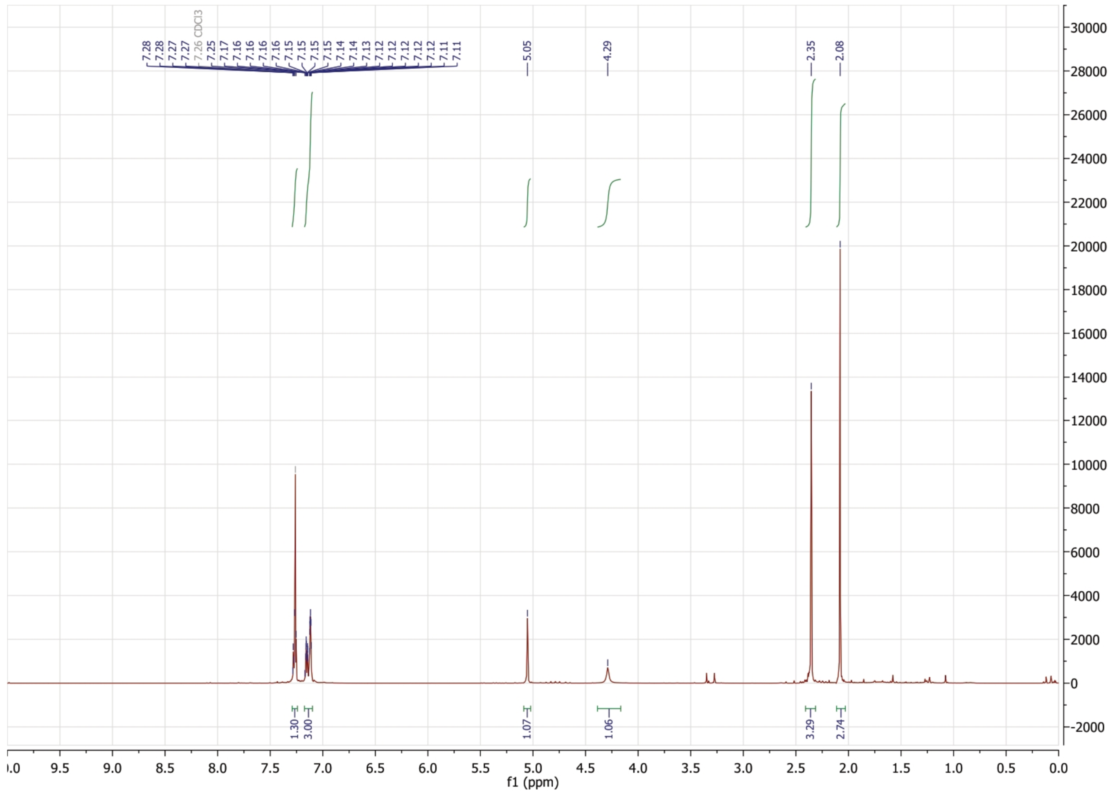

Figure S49. ^{1}H NMR spectrum of **3c** (600 MHz, $CDCl_3$).

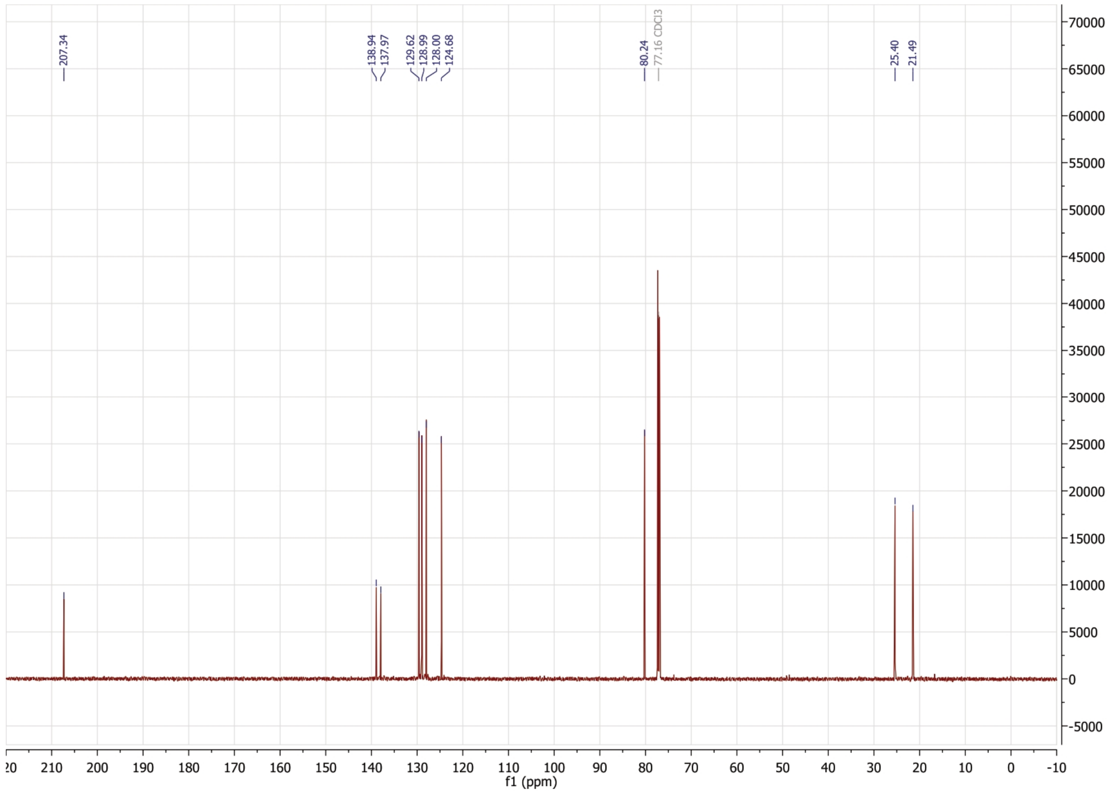

Figure S50. ^{13}C NMR spectrum of **3c** (151 MHz, $CDCl_3$).

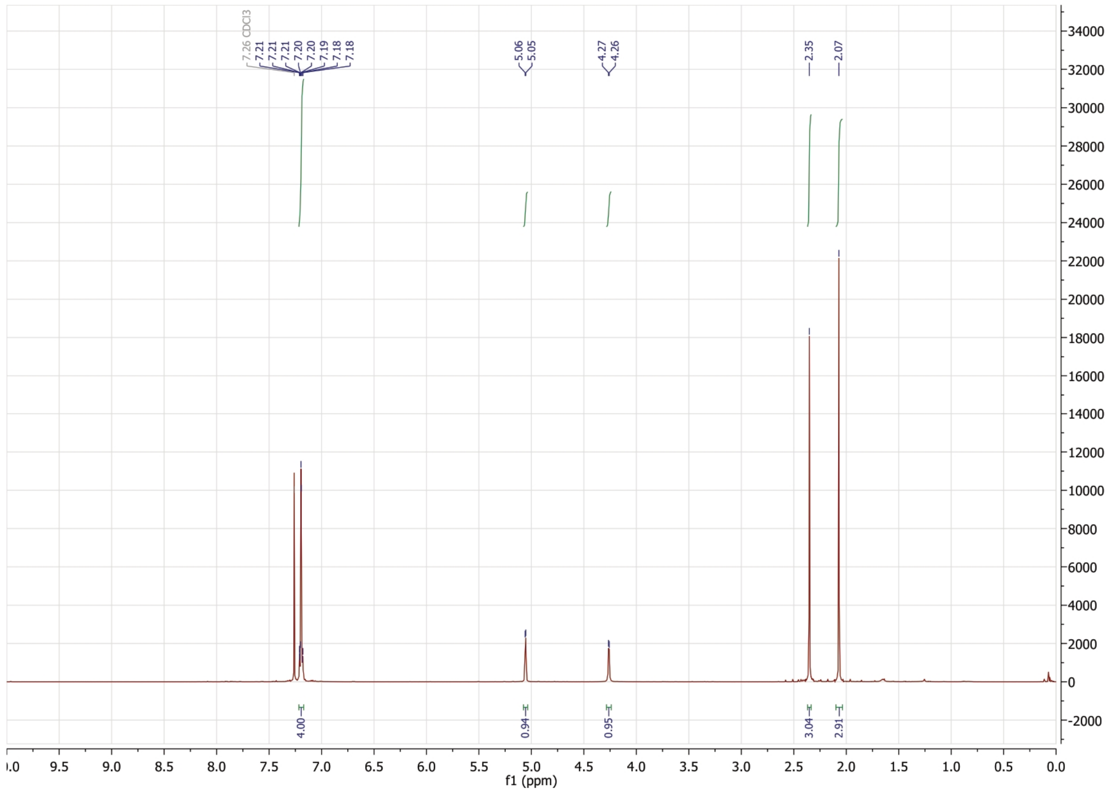

Figure S51. ^{1}H NMR spectrum of **3d** (600 MHz, $CDCl_3$).

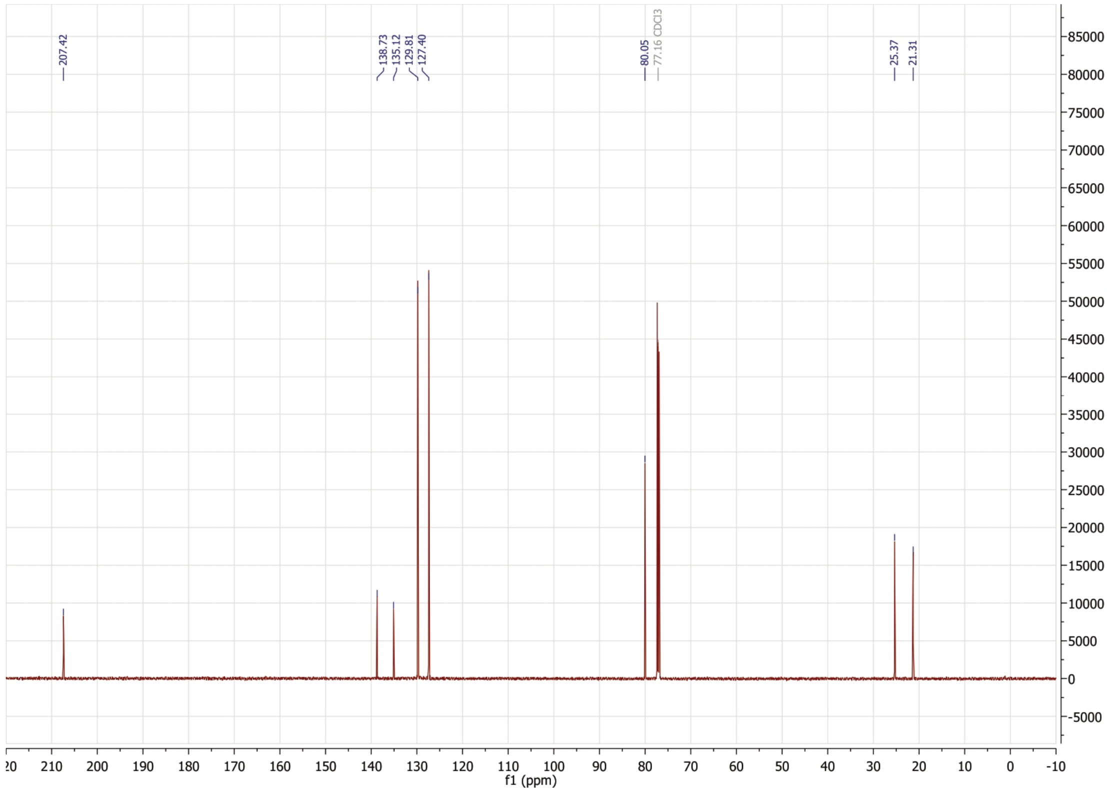

Figure S52. ^{13}C NMR spectrum of **3d** (151 MHz, $CDCl_3$).

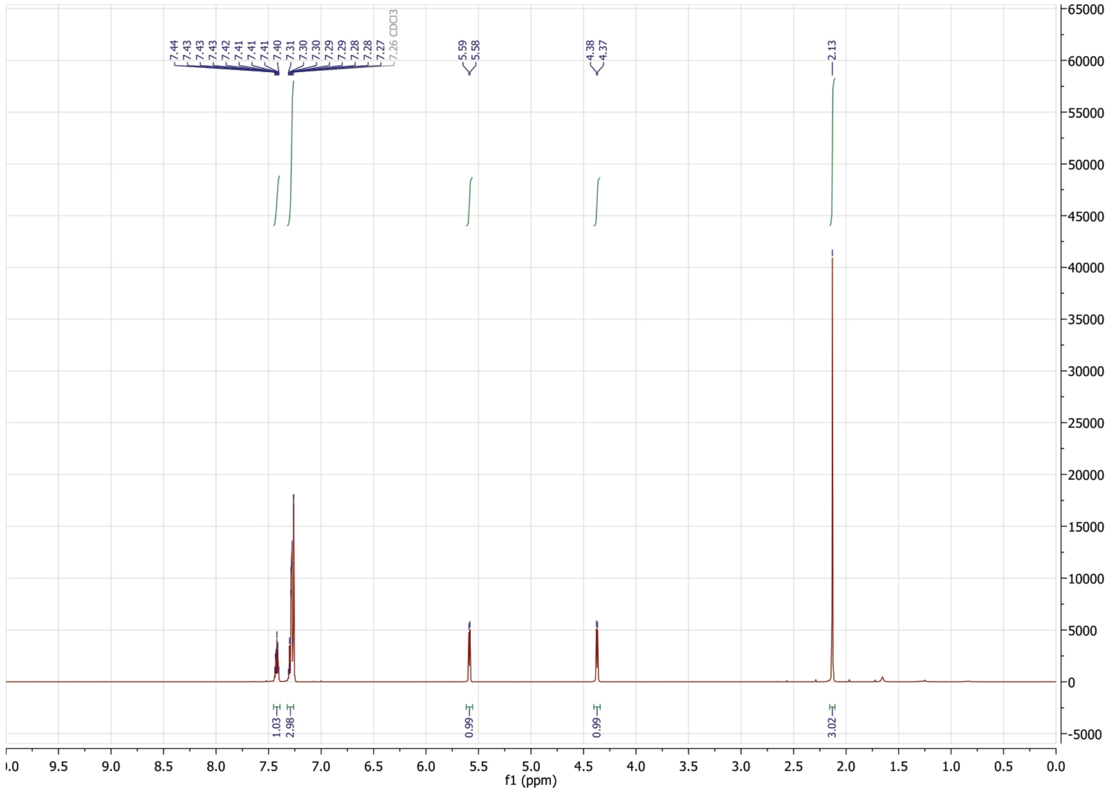

Figure S53. ^{1}H NMR spectrum of **3e** (400 MHz, $CDCl_3$).

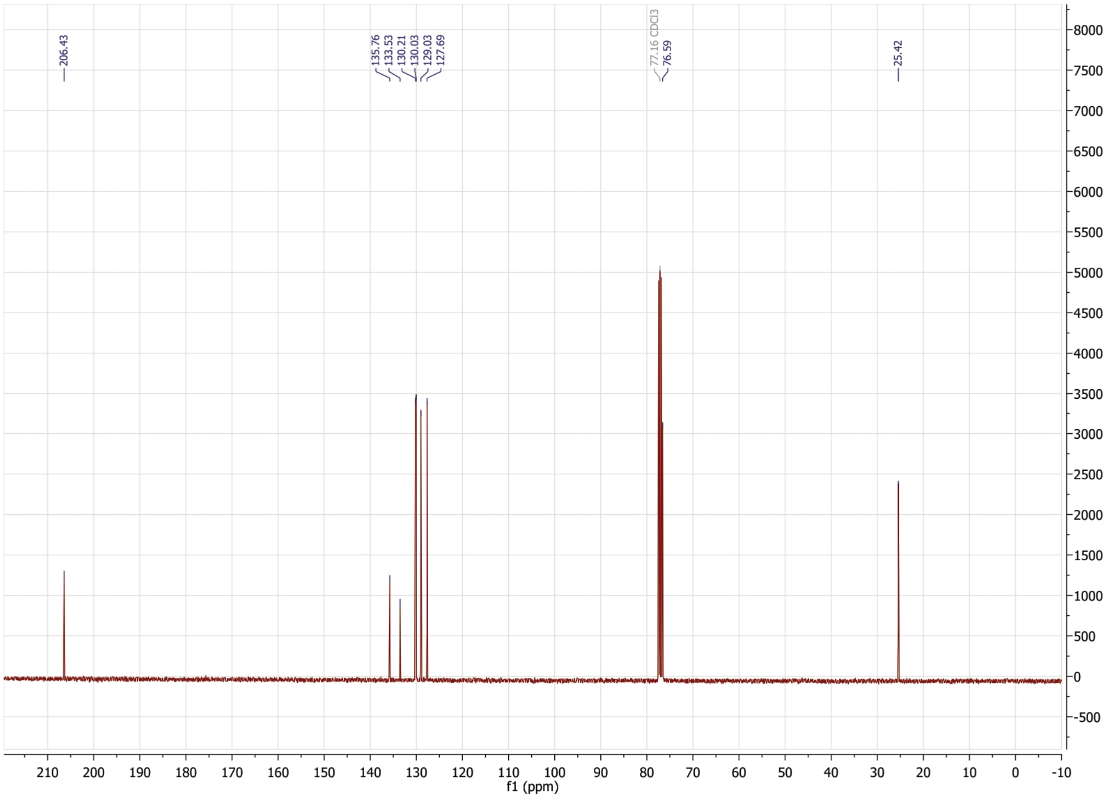

Figure S54. ^{13}C NMR spectrum of **3e** (101 MHz, $CDCl_3$).

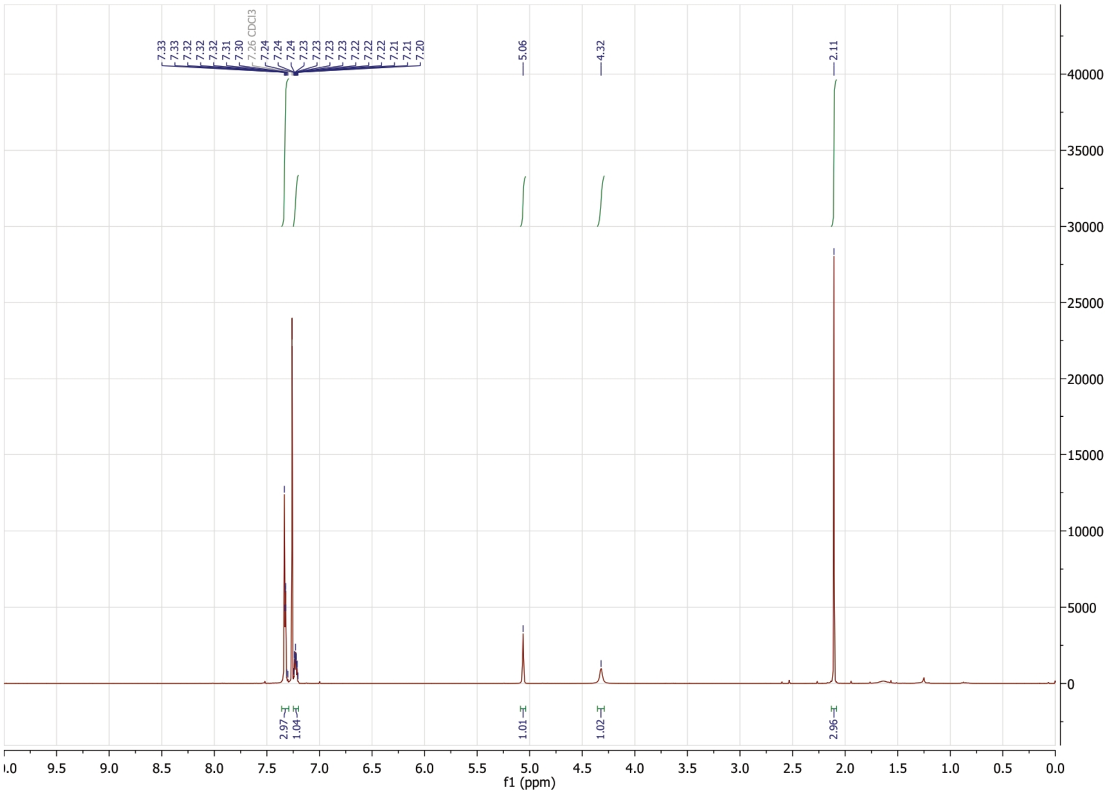

Figure S55. ^{1}H NMR spectrum of **3f** (400 MHz, $CDCl_3$).

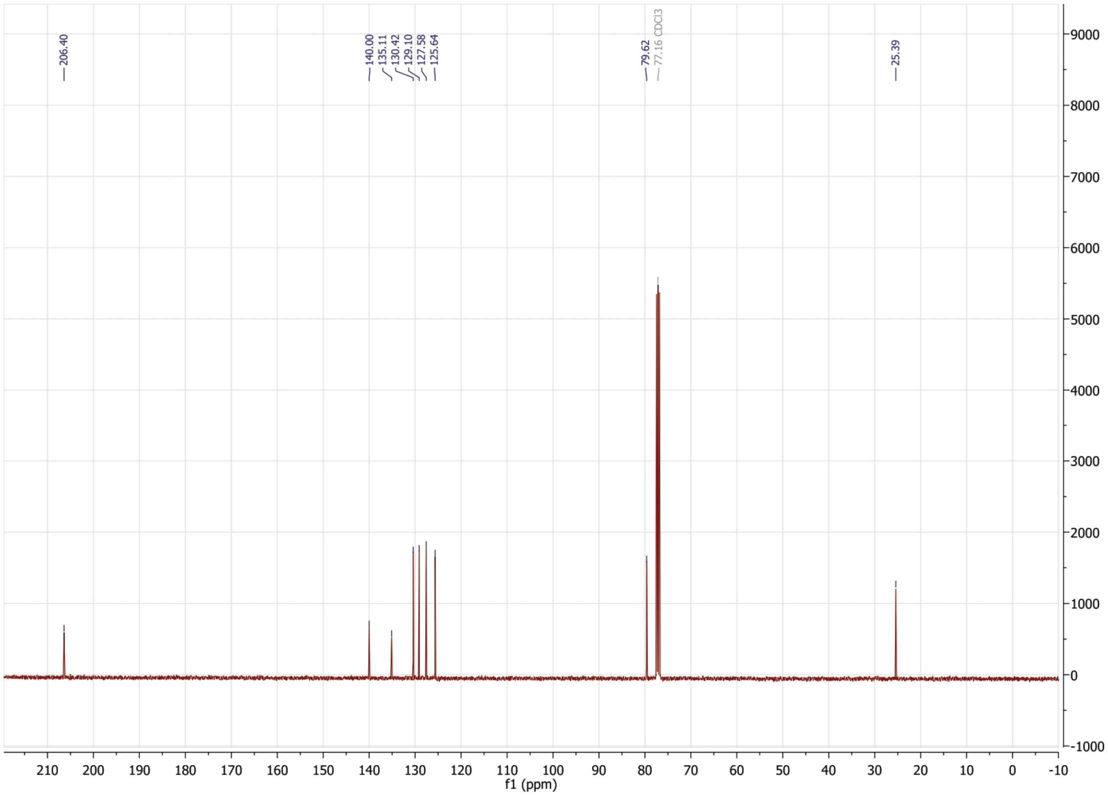

Figure S56. ^{13}C NMR spectrum of **3f** (101 MHz, $CDCl_3$).

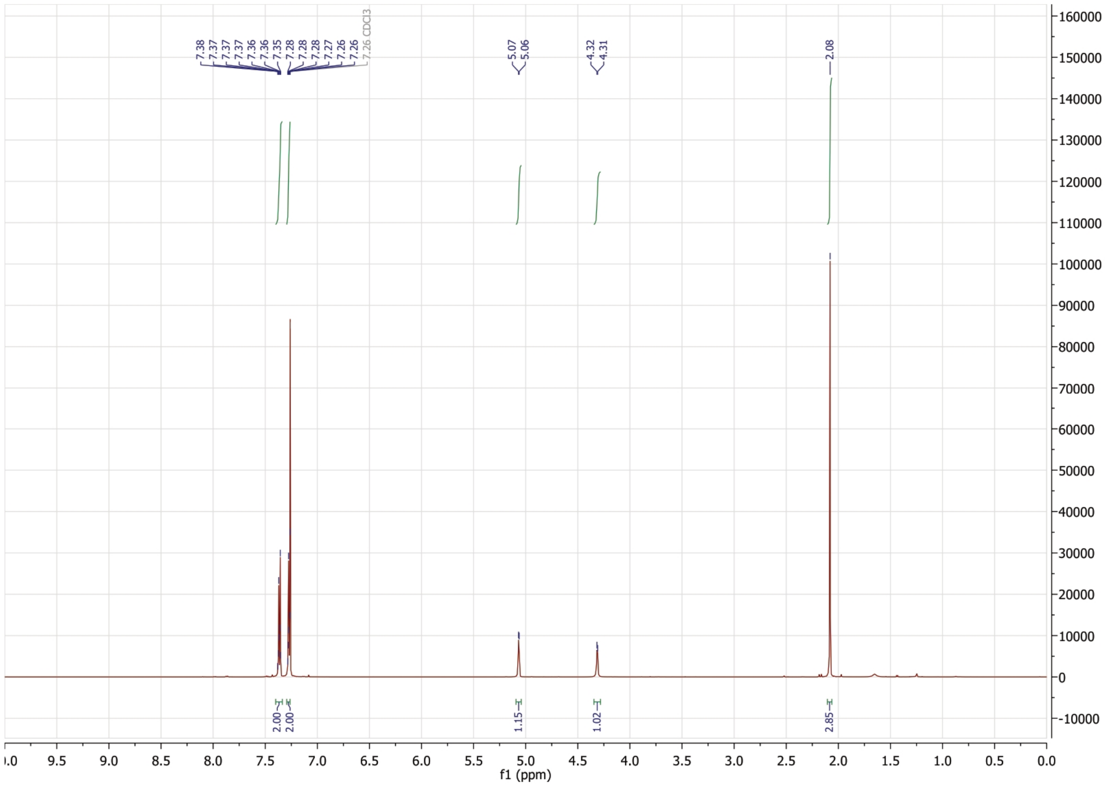

Figure S57. ^{1}H NMR spectrum of **3g** (600 MHz, $CDCl_3$).

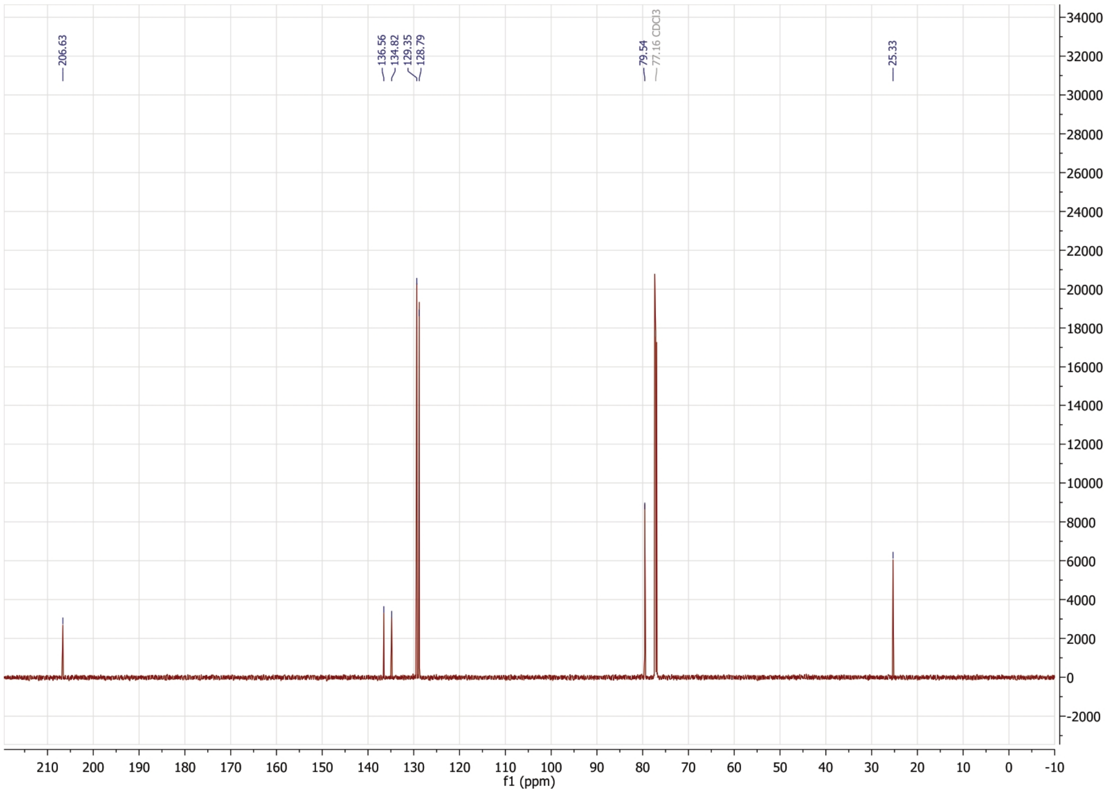

Figure S58. ^{13}C NMR spectrum of **3g** (151 MHz, $CDCl_3$).

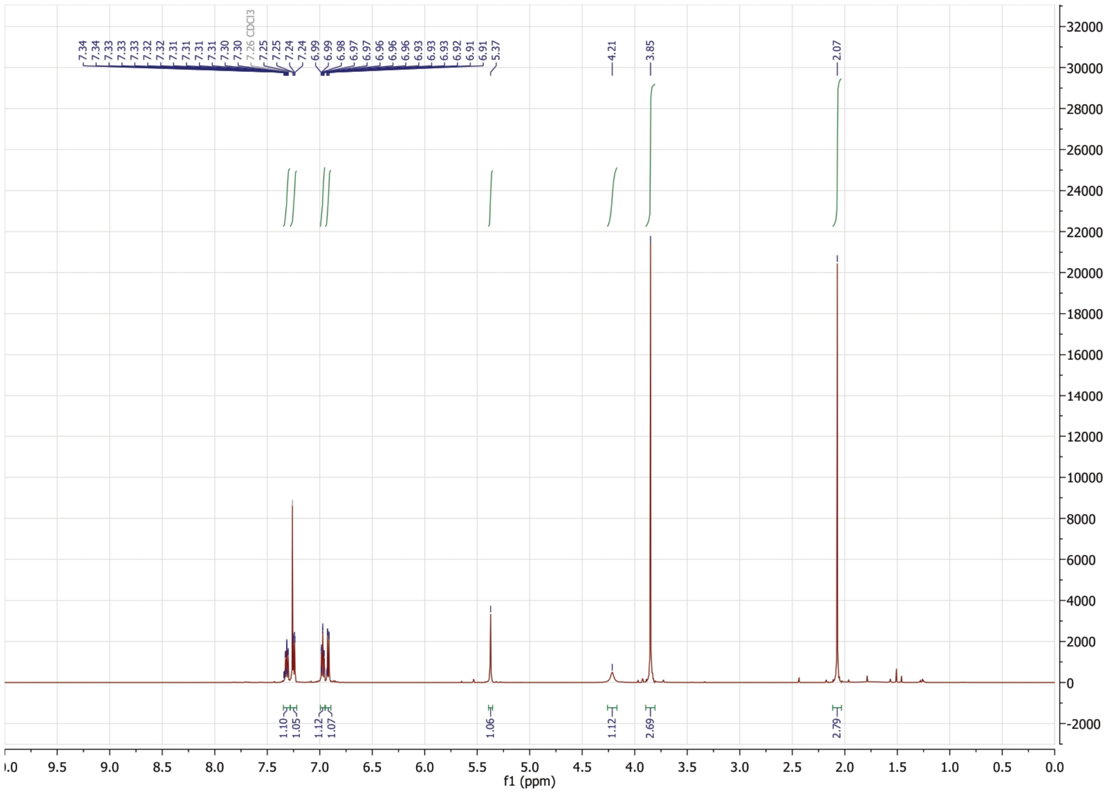

Figure S59. ^{1}H NMR spectrum of **3h** (600 MHz, $CDCl_3$).

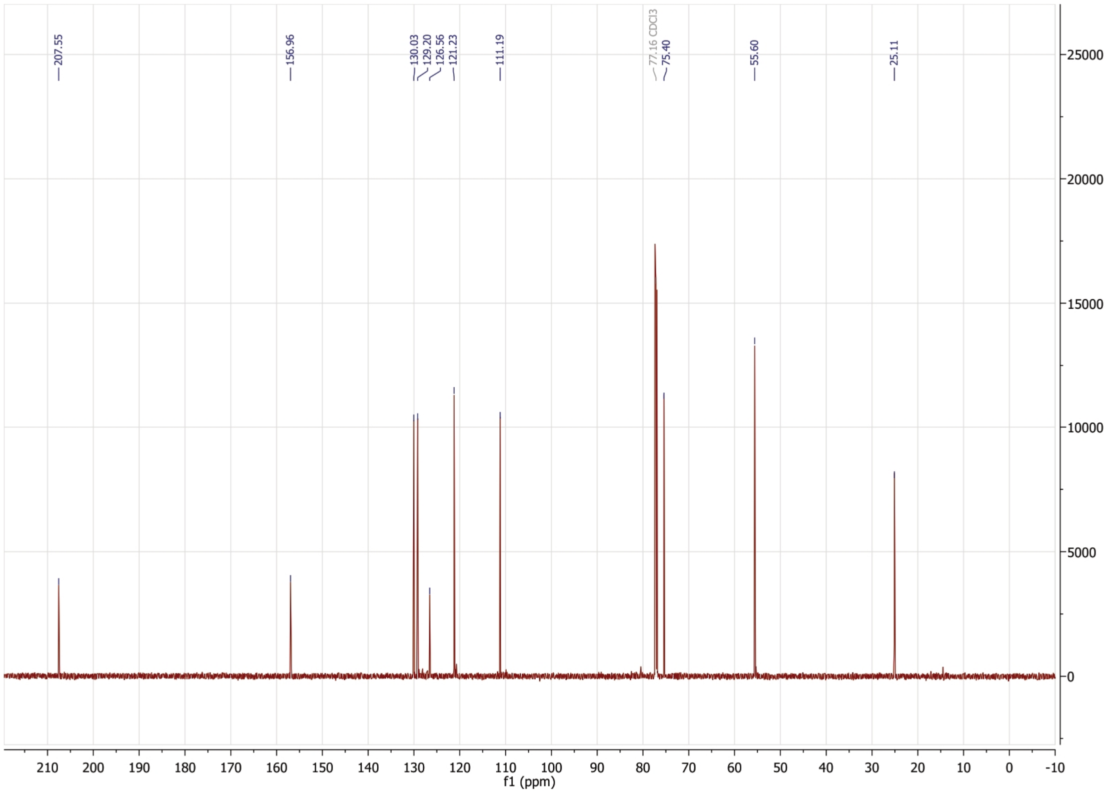

Figure S60. ^{13}C NMR spectrum of **3h** (151 MHz, $CDCl_3$).

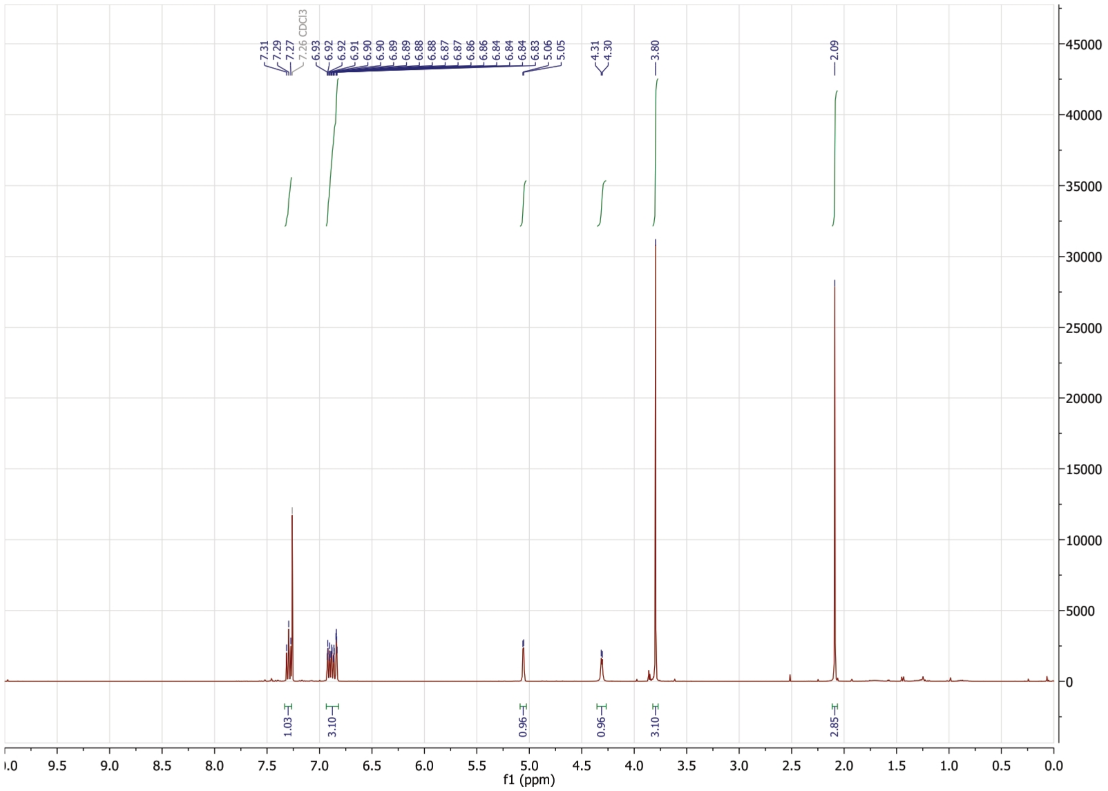

Figure S61. ^{1}H NMR spectrum of **3i** (400 MHz, $CDCl_3$).

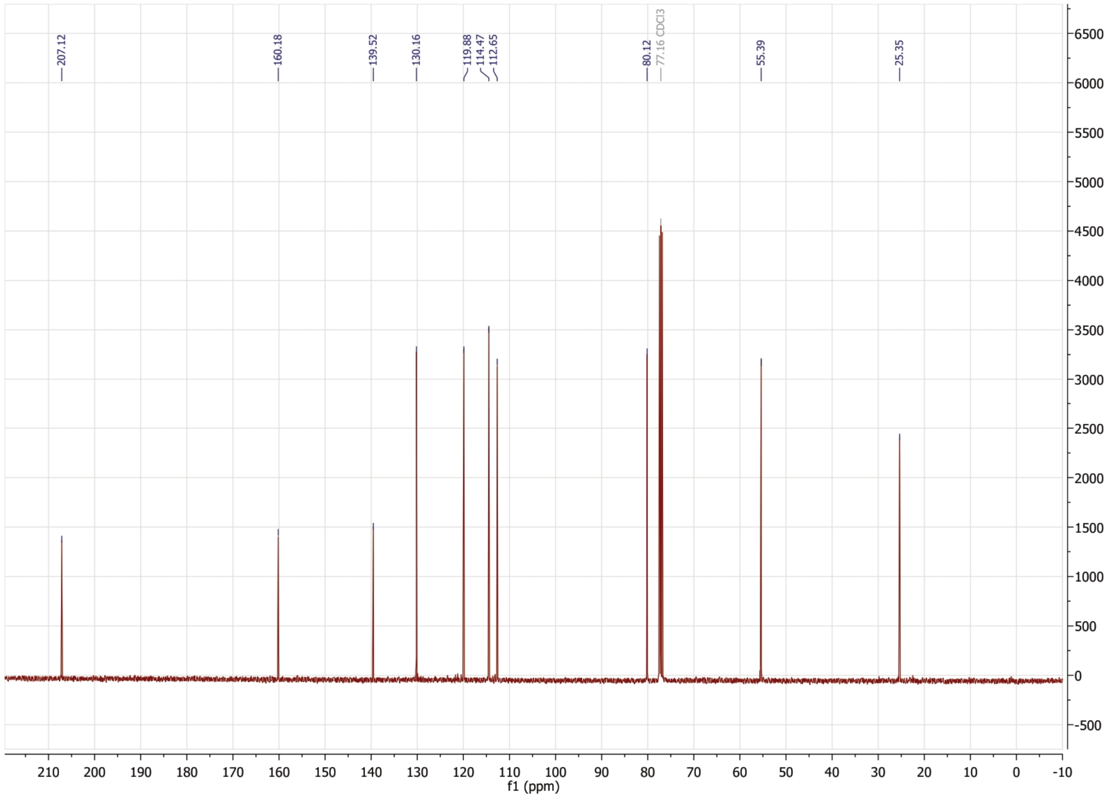

Figure S62. ^{13}C NMR spectrum of **3i** (101 MHz, $CDCl_3$).

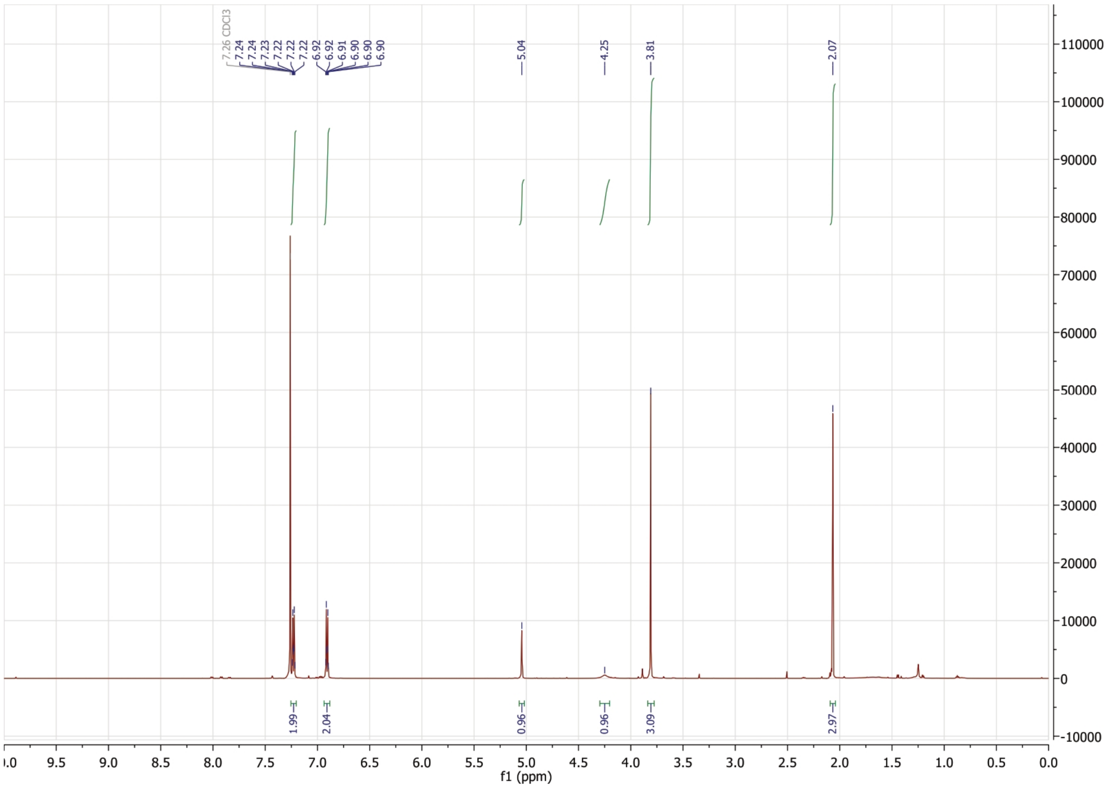

Figure S63. ^{1}H NMR spectrum of **3j** (600 MHz, $CDCl_3$).

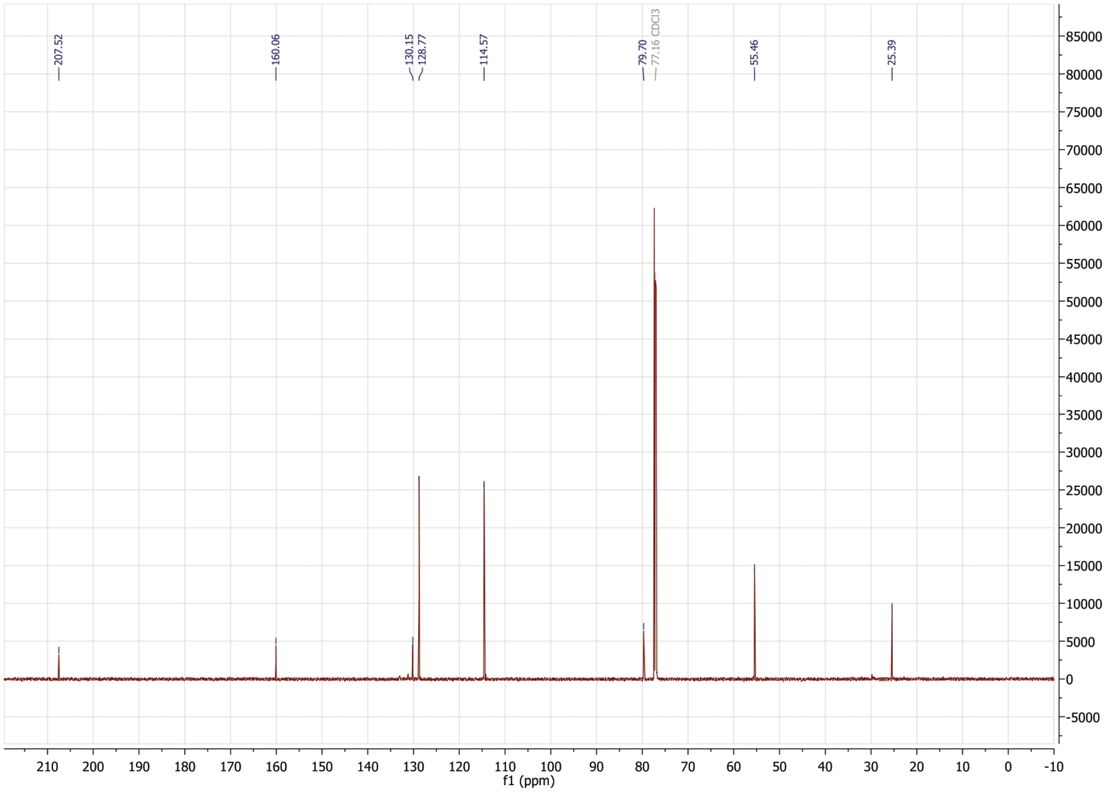

Figure S64. ^{13}C NMR spectrum of **3j** (151 MHz, $CDCl_3$).

11.3. ^{1}H and ^{13}C NMR analyses of furan derivatives

5-(Chloromethyl)furan-2-carbaldehyde:

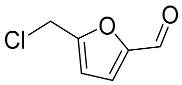

^{1}H NMR (400 MHz, $CDCl_3$) δ 9.62 (s, 1H), 7.19 (d, J = 3.6 Hz, 1H), 6.58 (d, J = 3.6 Hz, 1H), 4.60 (s, 2H); ^{13}C NMR (101 MHz, $CDCl_3$) δ 177.86, 156.15, 152.94, 121.92, 112.06, 36.63.

5-(Azidomethyl)furan-2-carbaldehyde **(1k):**

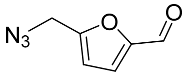

^{1}H NMR (600 MHz, $CDCl_3$) δ 9.62 (s, 1H), 7.22 (d, J = 3.6 Hz, 1H), 6.55 (d, J = 3.5 Hz, 1H), 4.41 (s, 2H); ^{13}C NMR (151 MHz, $CDCl_3$) δ 177.75, 155.44, 153.02, 121.95, 111.55, 47.07.

Furan-2,5-diyldimethanamine:

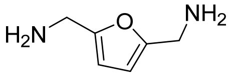

^{1}H NMR (400 MHz, D_2O) δ 6.16 (s, 2H), 3.72 (s, 4H); ^{13}C NMR (101 MHz, D_2O) δ 153.71, 107.06, 37.19.

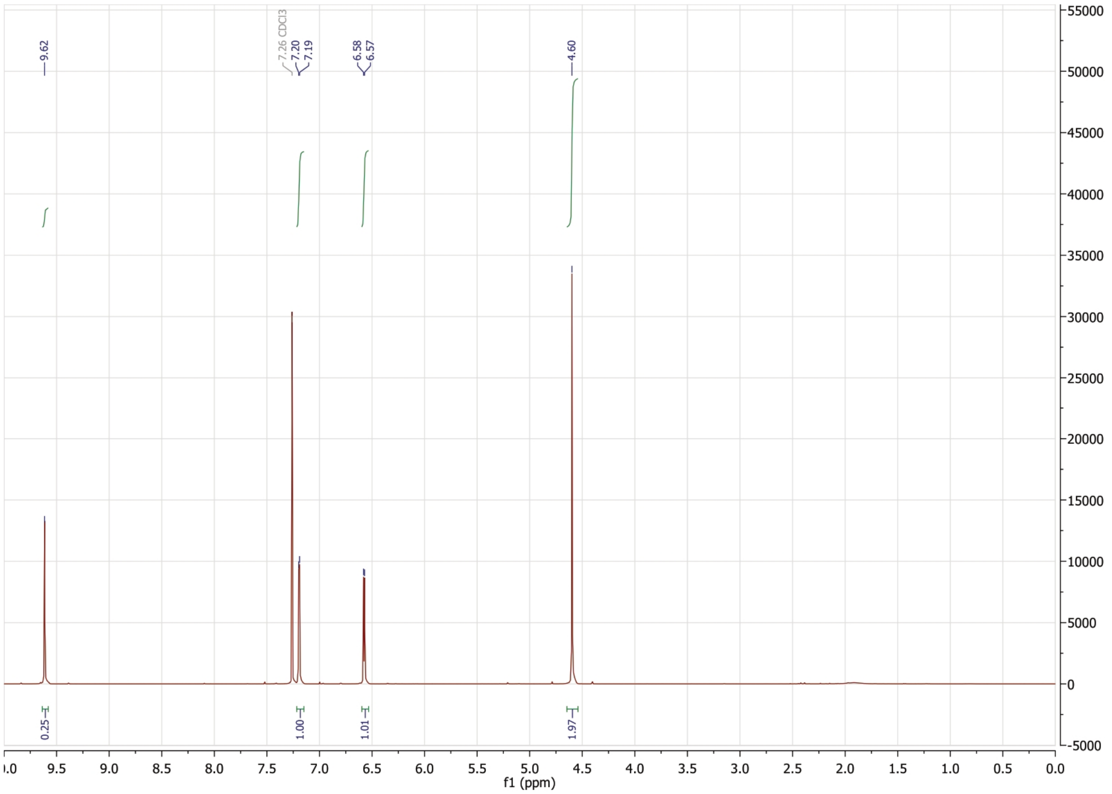

Figure S65. ^{1}H NMR spectrum of 5-(chloromethyl)furan-2-carbaldehyde (400 MHz, $CDCl_3$).

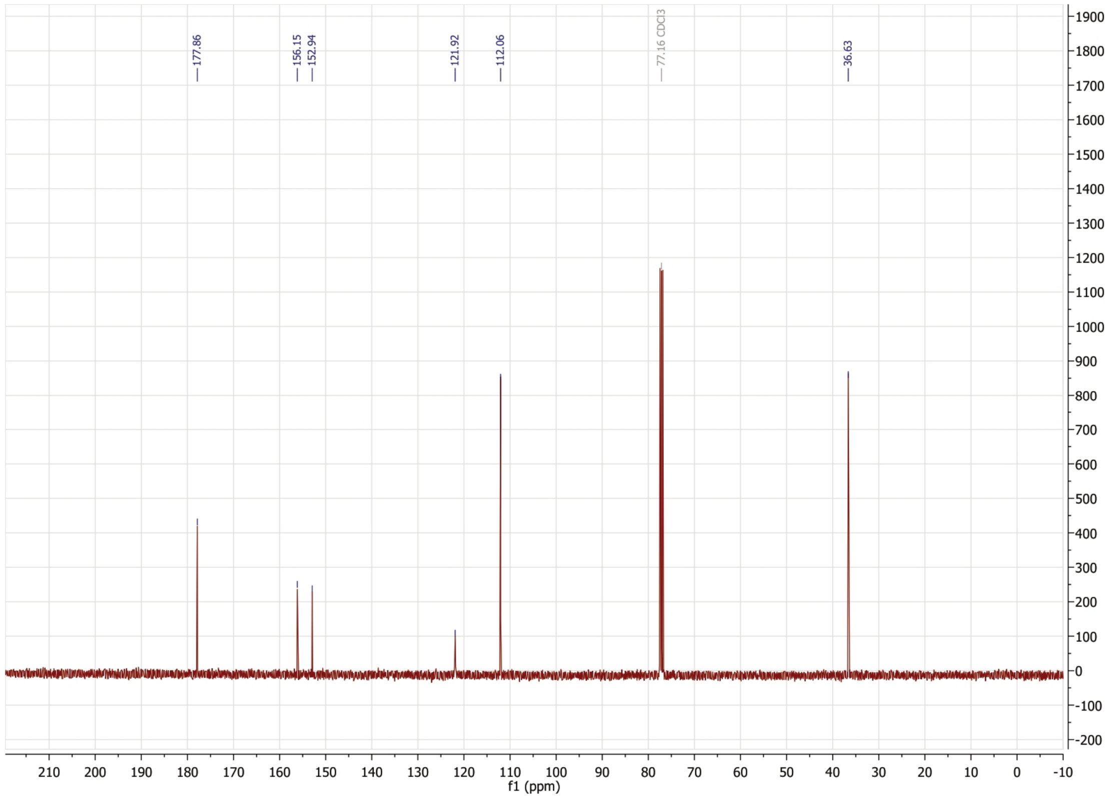

Figure S66. ^{13}C NMR spectrum of 5-(chloromethyl)furan-2-carbaldehyde (101 MHz, $CDCl_3$).

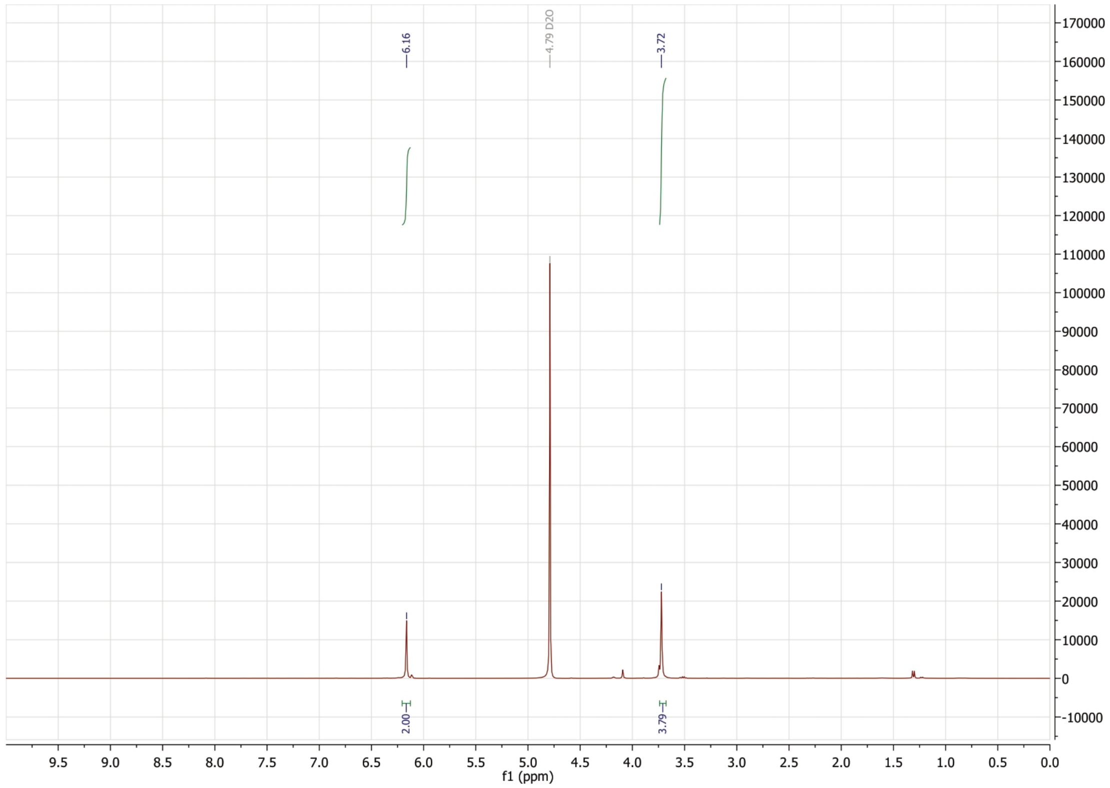

Figure S67. ^{1}H NMR spectrum of furan-2,5-diyldimethanamine (400 MHz, D_2O).

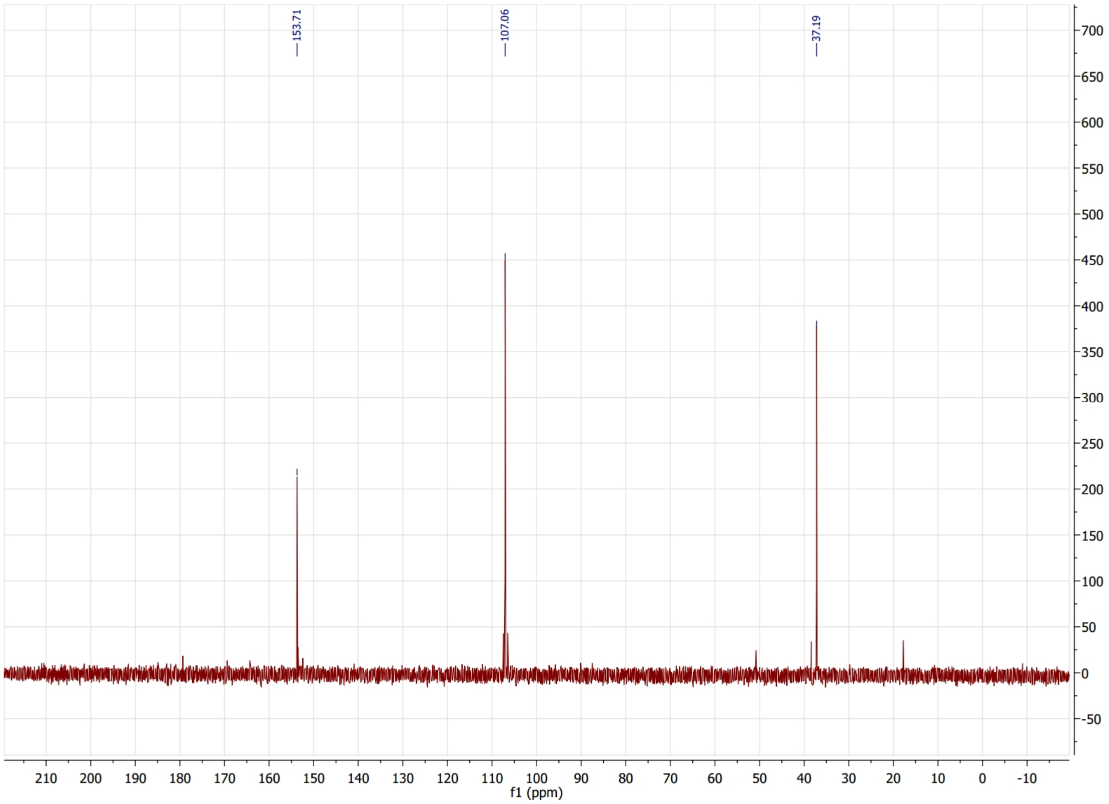

Figure S68. ^{13}C NMR spectrum of furan-2,5-diyldimethanamine (101 MHz, D_2O).

12. Qualitative mass spectrometric analysis

12.1. Qualitative analysis of compounds 2b–j by mass spectrometry

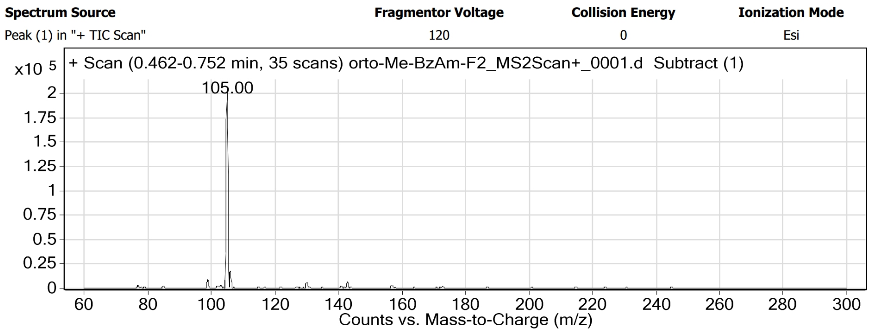

Figure S69. ESI-MS spectrum of compound **2b** in positive ion mode showing the $[M+H–NH_3]^+$ ion.

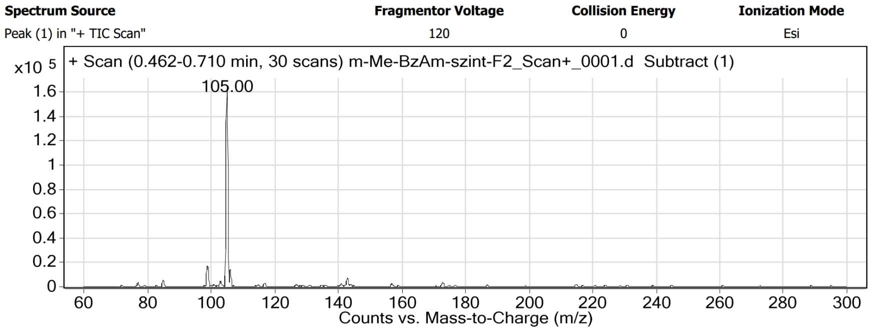

Figure S70. ESI-MS spectrum of compound **2c** in positive ion mode showing the $[M+H–NH_3]^+$ ion.

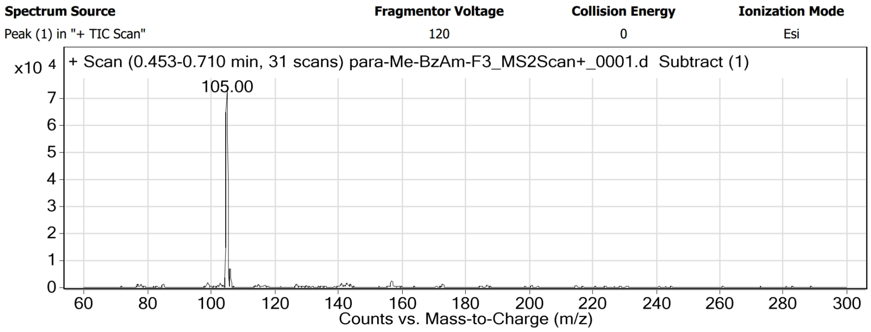

Figure S71. ESI-MS spectrum of compound **2d** in positive ion mode showing the $[M+H–NH_3]^+$ ion.

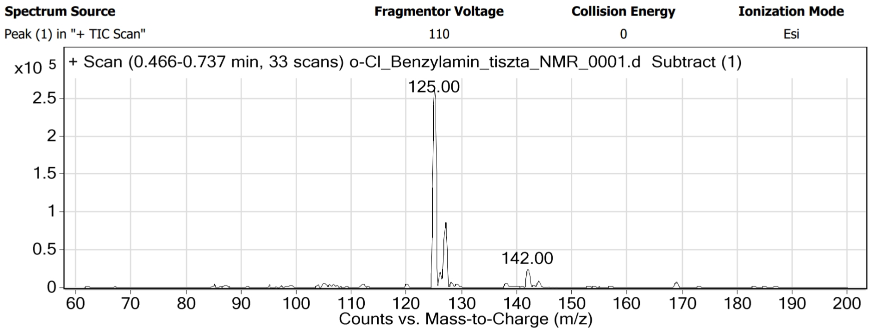

Figure S72. ESI-MS spectrum of compound **2e** in positive ion mode showing the $[M+H]^+$ and $[M+H–NH_3]^+$ ions.

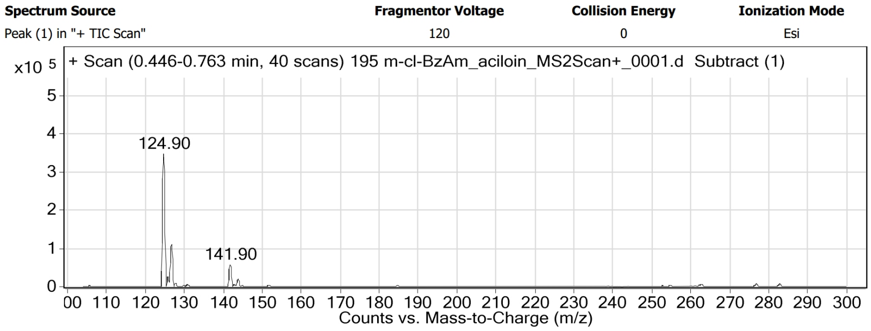

Figure S73. ESI-MS spectrum of compound **2f** in positive ion mode showing the $[M+H]^+$ and $[M+H–NH_3]^+$ ions.

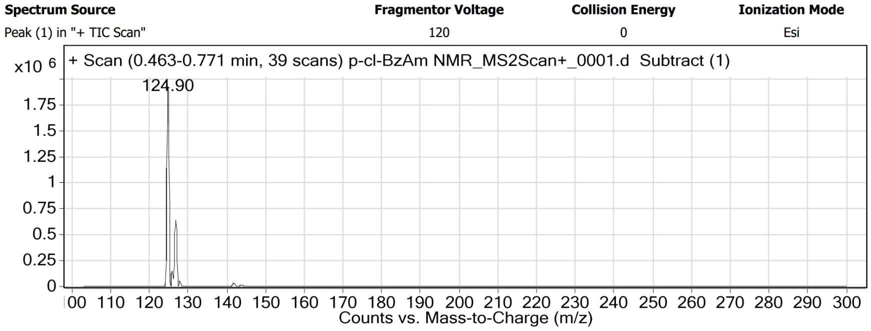

Figure S74. ESI-MS spectrum of compound **2g** in positive ion mode showing the $[M+H–NH_3]^+$ ion.

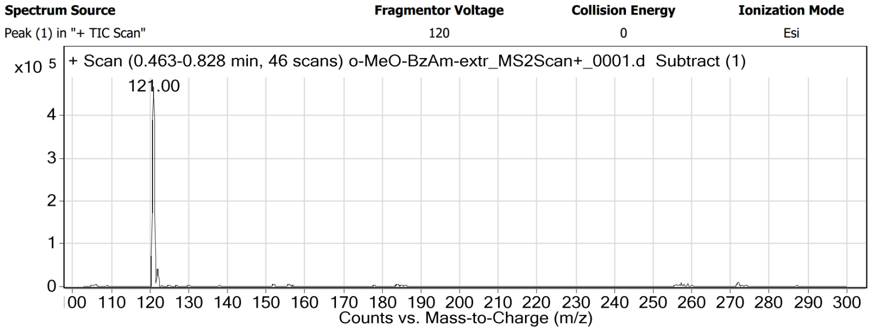

Figure S75. ESI-MS spectrum of compound **2h** in positive ion mode showing the $[M+H–NH_3]^+$ ion.

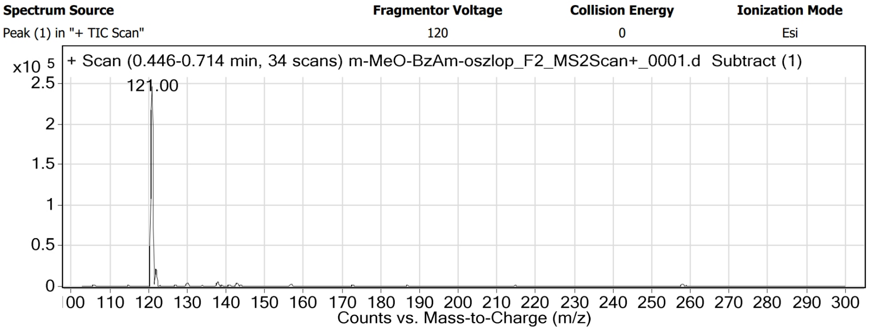

Figure S76. ESI-MS spectrum of compound **2i** in positive ion mode showing the $[M+H–NH_3]^+$ ion.

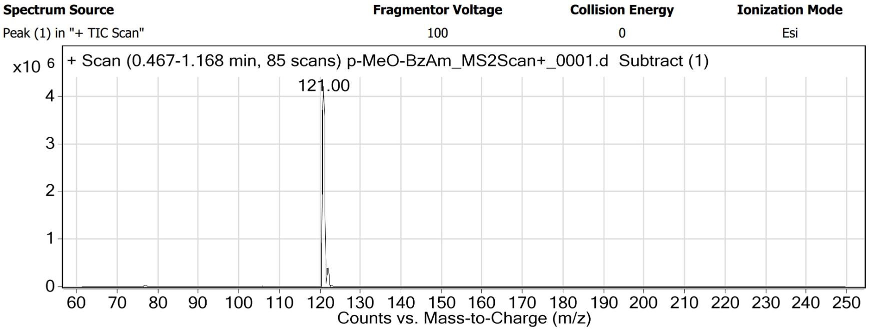

Figure S77. ESI-MS spectrum of compound **2j** in positive ion mode showing the $[M+H–NH_3]^+$ ion.

12.2. Qualitative analysis of furan derivatives by mass spectrometry

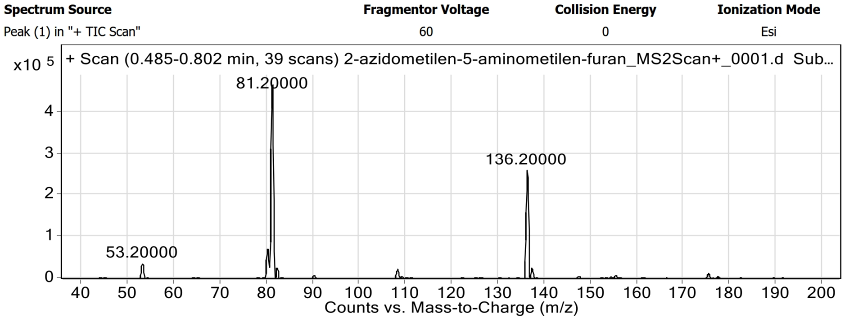

Figure S78. ESI-MS spectrum of compound **2k** in positive ion mode.

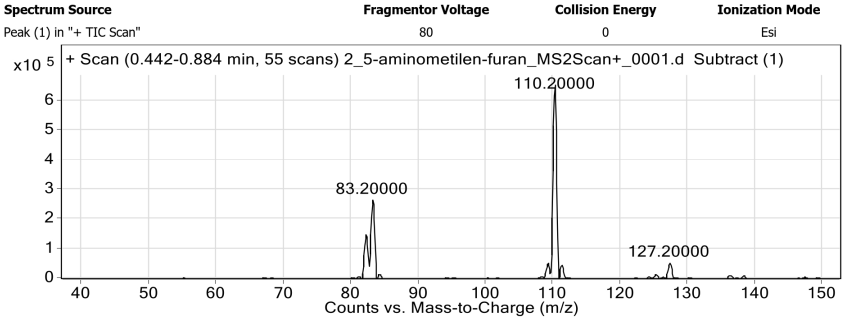

Figure S79. ESI-MS spectrum of furan-2,5-diyldimethanamine in positive ion mode.

13. Chiral separation of *rac*-3a–j enantiomers and determination of the optical purity of secondary products in the enzymatic cascade system

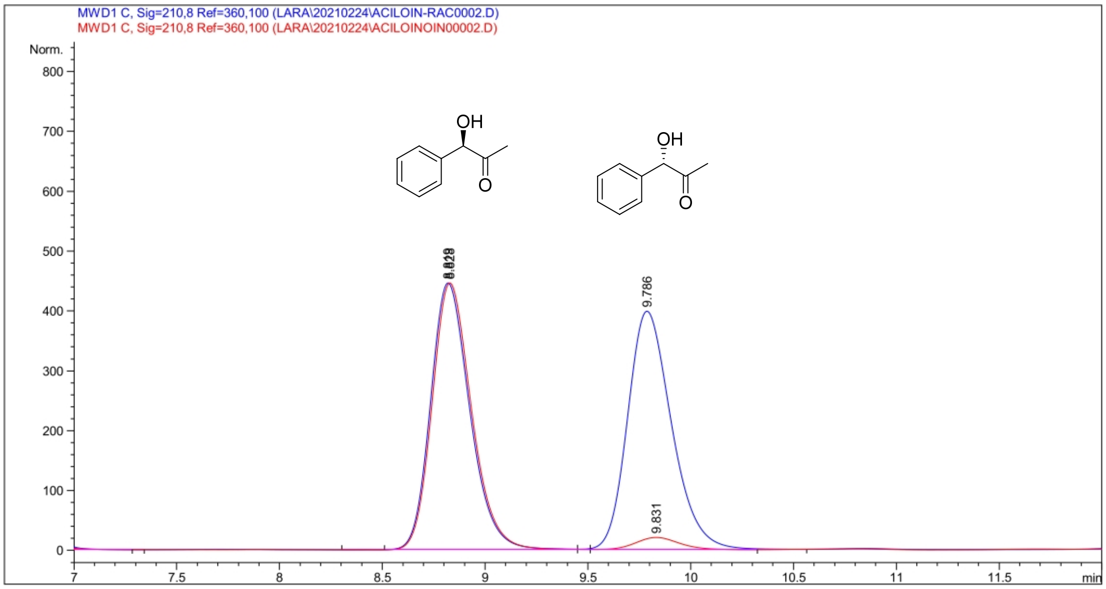

Figure S80. Chiral separation of the enantiomers of *rac*-**3a** and enantiomeric composition of **3a** formed in the *Zm*PDC-catalyzed carboligation reaction of **1a. Blue trace:** chiral separation of *rac*-**3a; red trace:** enantiomeric composition of **3a** obtained from the enzymatic reaction.

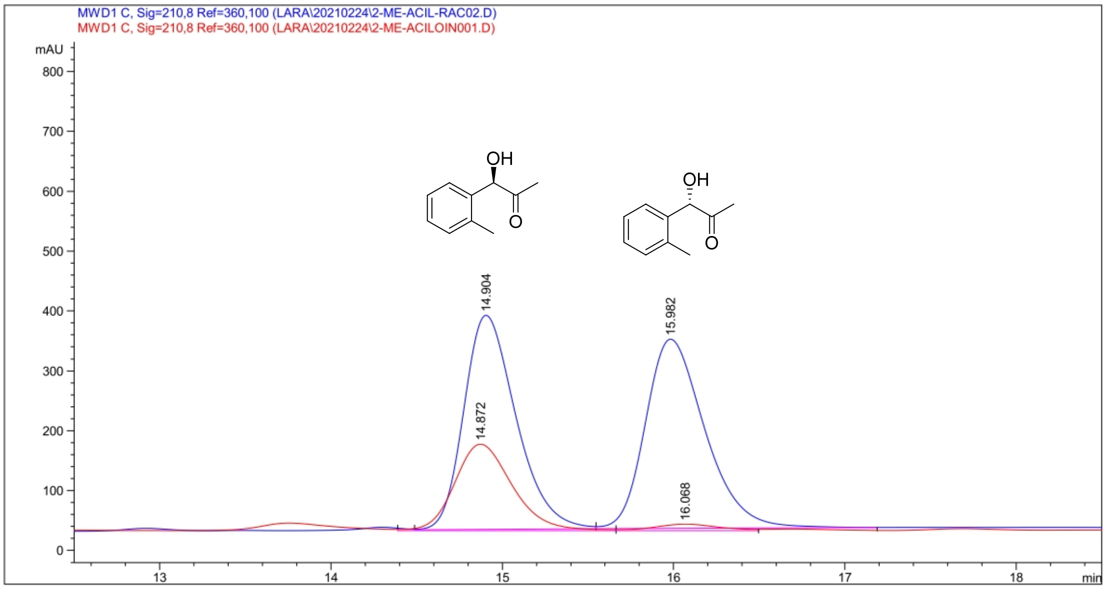

Figure S81. Chiral separation of the enantiomers of *rac*-**3b** and enantiomeric composition of **3b** formed in the *Zm*PDC-catalyzed carboligation reaction of **1b. Blue trace:** chiral separation of *rac*-**3b; red trace:** enantiomeric composition of **3b** obtained from the enzymatic reaction.

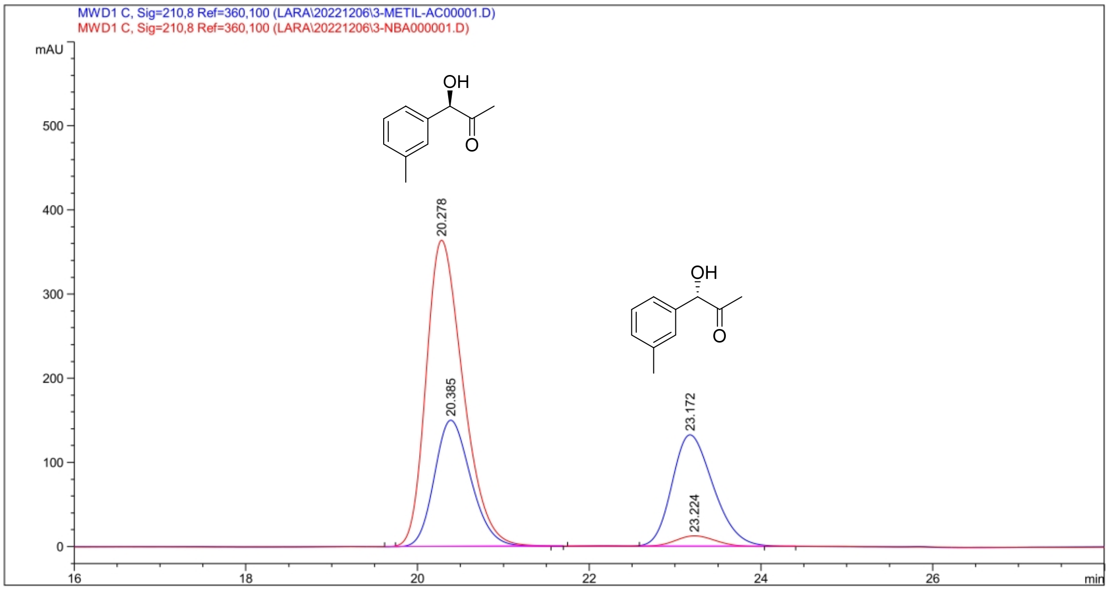

Figure S82. Chiral separation of the enantiomers of *rac*-**3c** and enantiomeric composition of **3c** formed in the *Zm*PDC-catalyzed carboligation reaction of **1c. Blue trace:** chiral separation of *rac*-**3c; red trace:** enantiomeric composition of **3c** obtained from the enzymatic reaction.

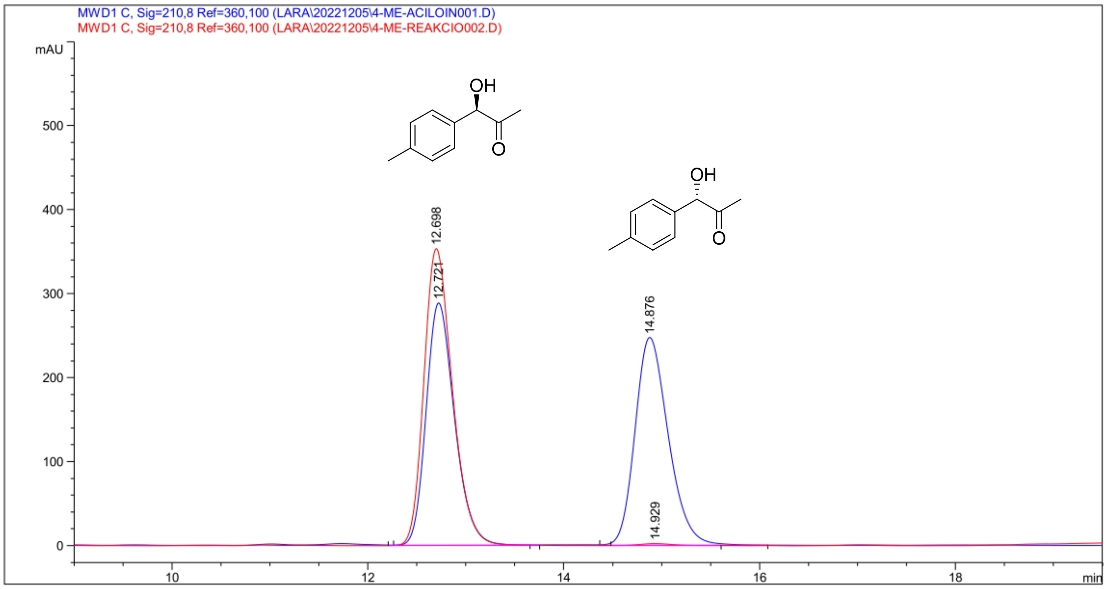

Figure S83. Chiral separation of the enantiomers of *rac*-**3d** and enantiomeric composition of **3d** formed in the *Zm*PDC-catalyzed carboligation reaction of **1d. Blue trace:** chiral separation of *rac*-**3d; red trace:** enantiomeric composition of **3d** obtained from the enzymatic reaction.

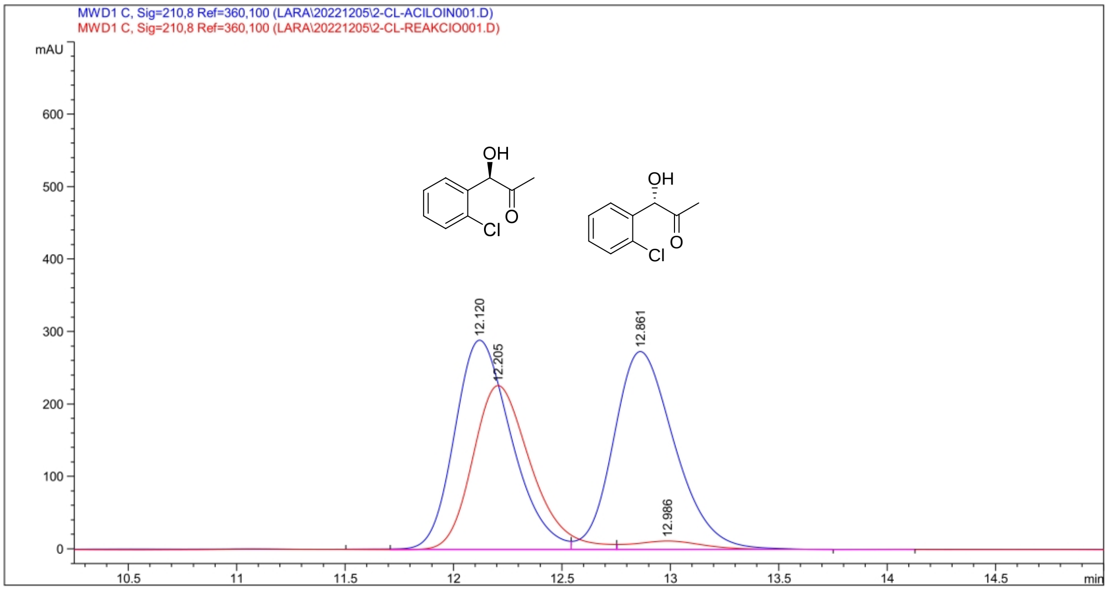

Figure S84. Chiral separation of the enantiomers of *rac*-**3e** and enantiomeric composition of **3e** formed in the *Zm*PDC-catalyzed carboligation reaction of **1e. Blue trace:** chiral separation of *rac*-**3e; red trace:** enantiomeric composition of **3e** obtained from the enzymatic reaction.

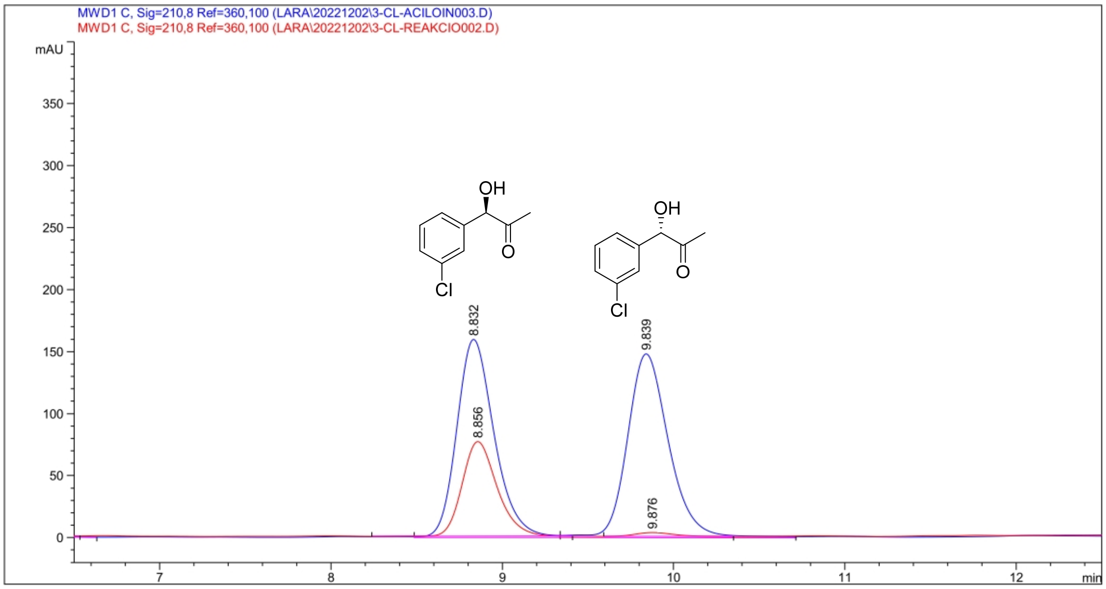

Figure S85. Chiral separation of the enantiomers of *rac*-**3f** and enantiomeric composition of **3f** formed in the *Zm*PDC-catalyzed carboligation reaction of **1f. Blue trace:** chiral separation of *rac*-**3f; red trace:** enantiomeric composition of **3f** obtained from the enzymatic reaction.

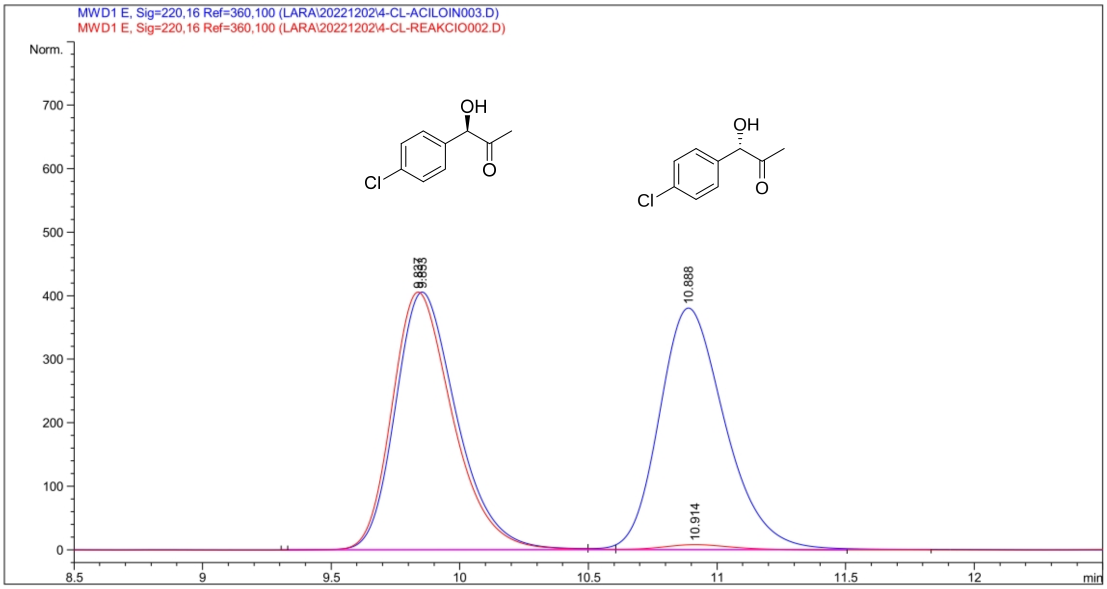

Figure S86. Chiral separation of the enantiomers of *rac*-**3g** and enantiomeric composition of **3g** formed in the *Zm*PDC-catalyzed carboligation reaction of **1g. Blue trace:** chiral separation of *rac*-**3g; red trace:** enantiomeric composition of **3g** obtained from the enzymatic reaction.

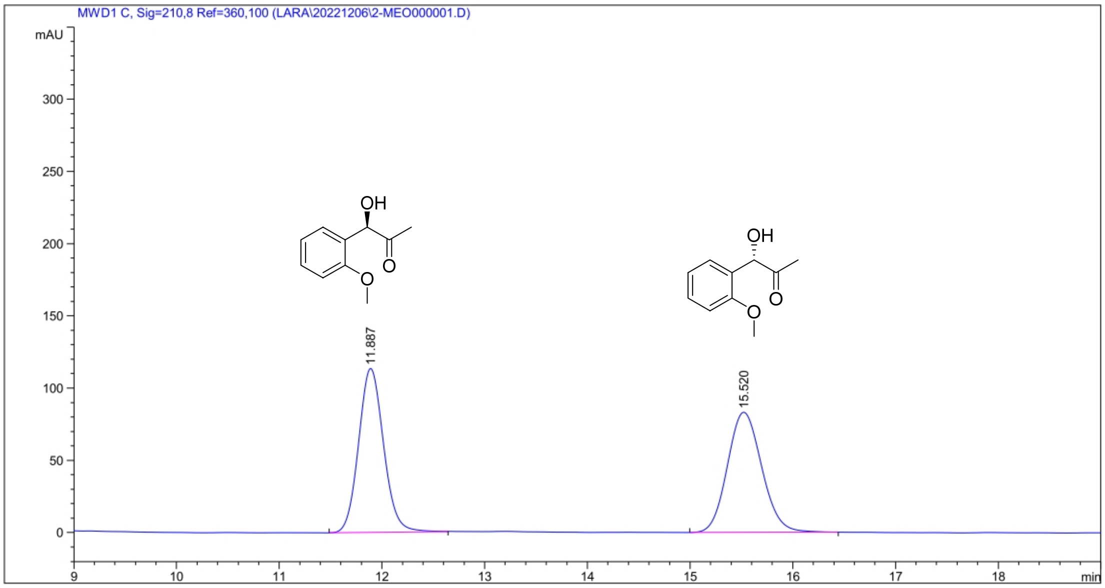

Figure S87. Chiral separation of the enantiomers of *rac*-**3h.**

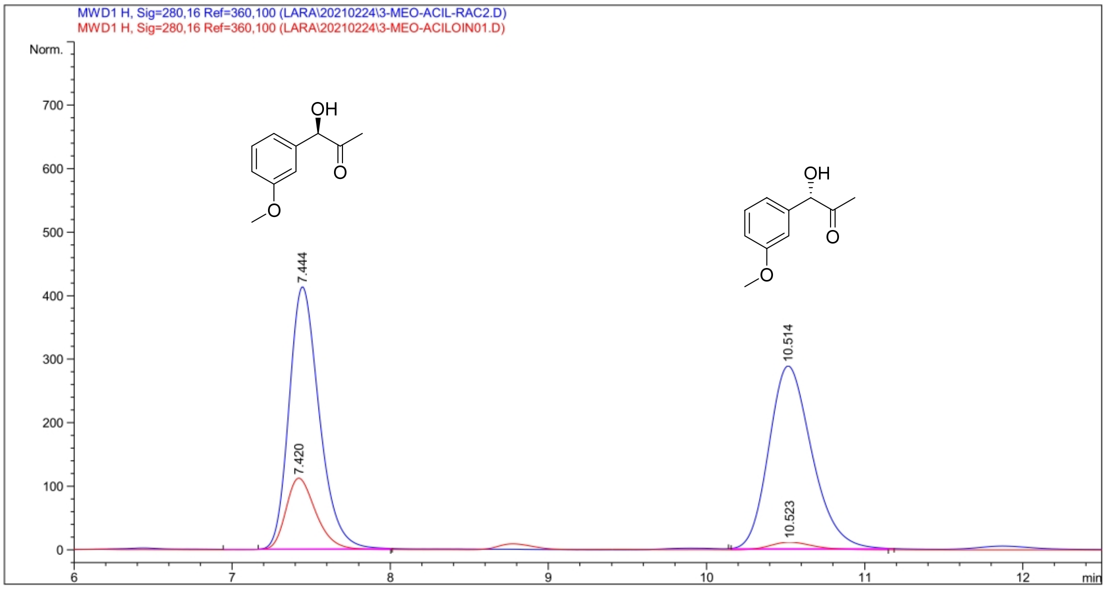

Figure S88. Chiral separation of the enantiomers of *rac*-**3i** and enantiomeric composition of **3i** formed in the *Zm*PDC-catalyzed carboligation reaction of **1i. Blue trace:** chiral separation of *rac*-**3i; red trace:** enantiomeric composition of **3i** obtained from the enzymatic reaction.

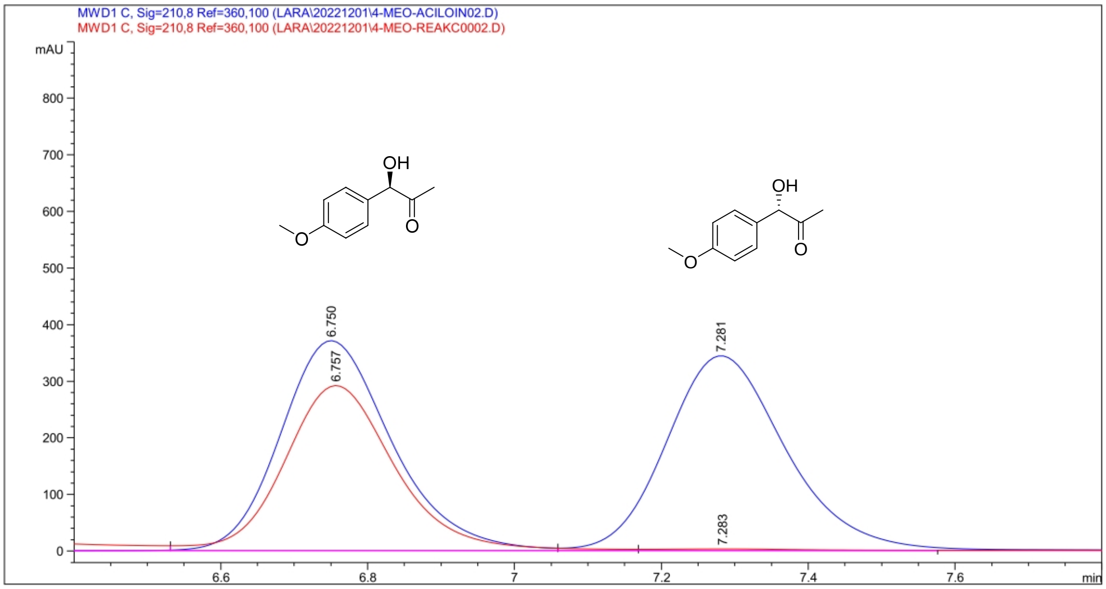

Figure S89. Chiral separation of the enantiomers of *rac*-**3j** and enantiomeric composition of **3j** formed in the *Zm*PDC-catalyzed carboligation reaction of **1j. Blue trace:** chiral separation of *rac*-**3j; red trace:** enantiomeric composition of **3j** obtained from the enzymatic reaction.

14. Supplementary results

14.1. Supporting experimental data and analytical conditions

Table S9. Comparative analysis of benzylamine **(2a)** formation at varying initial substrate concentrations **(1a).**

Substrate concentration (1a) (mM)	Yield of 2a after 2 h (%)	Concentration of formed 2a (mg mL^{-1})
30	34.3	1.1
40	31	1.3
50	29.2	**1.6**
60	20.5	1.3
70	6.7	0.5

Table S10. Effect of substrate **(1a–j)** concentration on conversion and product **(2a–j)** formation using cotransformed *PpS*-TA–*Zm*PDC in the RARE *E. coli* strain.

Substrate	Substrate concentration (mM)	Yield of amine after 3 h (%)	Amine concentration (mg mL^{-1})
**1a**	30	45.6	1.5
	40	42.3	1.8
	50	70.7	**3.8**
	60	33.4	2.1
	70	23.6	1.8
**1b**	10	92.5	1.1
	20	76.9	**1.9**
	30	34.9	1.3
	40	8.8	0.4
**1c**	10	83.3	1.0
	20	66.3	1.6
	30	63.5	**2.3**
	40	14.8	0.7
**1d**	10	62.3	0.8
	20	45.5	1.1
	30	49.6	**1.8**
	40	8.4	0.4
**1e**	10	90.7	1.3
	20	97.1	2.8
	30	78.7	**3.3**
	40	25.5	1.4
**1f**	10	85.6	1.2
	20	76.1	**2.2**
	30	41.6	1.8
	40	15.6	0.9
**1g**	10	77.7	1.1
	20	73.6	**2.1**
	30	37.1	1.6
	40	12.1	0.7
**1h**	10	97.2	1.3
	20	79.5	**2.2**
	30	35.0	1.4
	40	23.5	1.3
**1i**	10	84.9	1.2
	20	74.9	2.1
	30	66.8	**2.7**
	40	17.2	0.9
**1j**	10	43.5	0.6
	20	39.9	1.1
	30	40.0	**1.6**
	40	15.1	0.8

Table S11. MS parameters used for LC–MS/MS analysis.

Compound	MRM transitions used for quantification	Fragmentor (V)	Collision energy (V)
**2a**	108.1 → 91.1	80	15
(*R*)-**3a**	133.0 → 105.0	100	8
IS	310.2 → 237.2	80	15

Table S12. Ion-exchange chromatography gradient program for purification of **2a–k.**

Time (min.)	Bidistilled H_2O (%)	NH_3 solution (2M) (%)
0–30	100	0
30–60	95	5
60–120	90	10
120–150	40	60
150–180	0	100
180–220	100	0

14.2. Quantification of alcohol byproducts

Table S13 summarizes the formation of alcohol byproducts at different cell loadings and reaction times for substrates **1b–j.**

Table S13. Dependence of alcohol byproduct formation on cell loading and reaction time in the biocatalytic amination of 20 mM aldehyde **(1b–j).**

Substrate	Alcohol yield after 2 h (%)			Alcohol yield after 4 h (%)			Alcohol yield after 6 h (%)		
	3 mg mL^{-1}	6 mg mL^{-1}	9 mg mL^{-1}	3 mg mL^{-1}	6 mg mL^{-1}	9 mg mL^{-1}	3 mg mL^{-1}	6 mg mL^{-1}	9 mg mL^{-1}
**1b**	4.5	6.5	8.1	4.6	9.2	12.7	4.8	13.2	19.9
**1c**	3.3	7.5	9.3	4.7	9.8	13.5	5.5	13.5	22.3
**1d**	5.6	10.2	13.7	8.5	14.3	19.5	10.4	19.6	29.4
**1e**	6.4	7.1	8.8	6.4	8.3	8.9	6.4	9.7	14.9
**1f**	0.8	2.8	6	1.3	4.2	9	1.7	6.9	10.7
**1g**	1.8	6.1	11.7	2.2	8.2	16.6	2.5	9.9	23.8
**1h**	0.6	3.1	4.1	1.8	4.5	6.25	2.5	5.8	8.9
**1i**	0.7	3.5	11.5	1.8	5.7	17	3.2	8.05	22.7
**1j**	3.4	8.6	9.2	6.4	14.6	18.2	8.7	21.4	26

3, 6, and 9 mg mL^{-1} refer to the amount of lyophilized cells used in the reactions.

Calibration curves were constructed over the concentration ranges 0.244–29.32 μg mL^{-1} (methylbenzyl alcohols), 0.285–34.22 μg mL^{-1} (chlorobenzyl alcohols), and 0.276–33.16 μg mL^{-1} (methoxybenzyl alcohols). The same HPLC methods described in Table S3 were used in all cases, with the alcohol byproduct eluting before the acyloins.

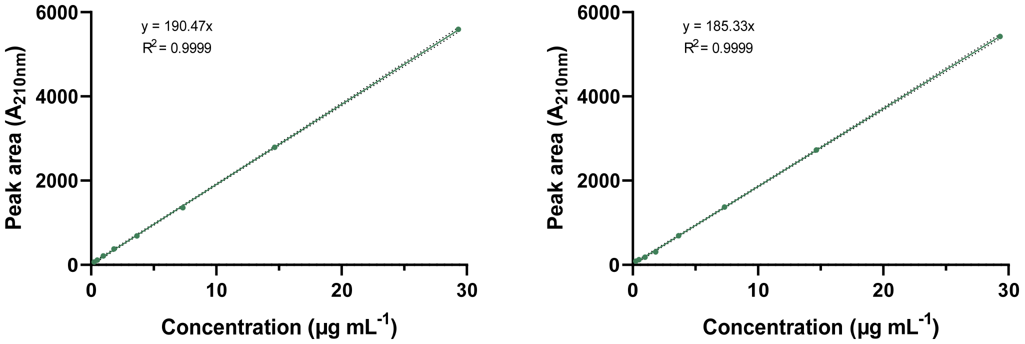

a. **b.**

Figure S90. Calibration curves at 210 nm for **(a)** 2-methylbenzyl alcohol and **(b)** 3-methylbenzyl alcohol.

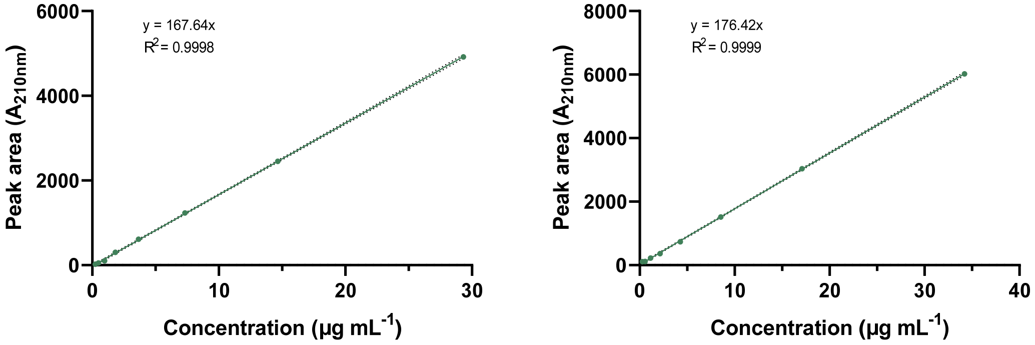

a. **b.**

Figure S91. Calibration curves at 210 nm for **(a)** 4-methylbenzyl alcohol and **(b)** 2-chlorobenzyl alcohol.

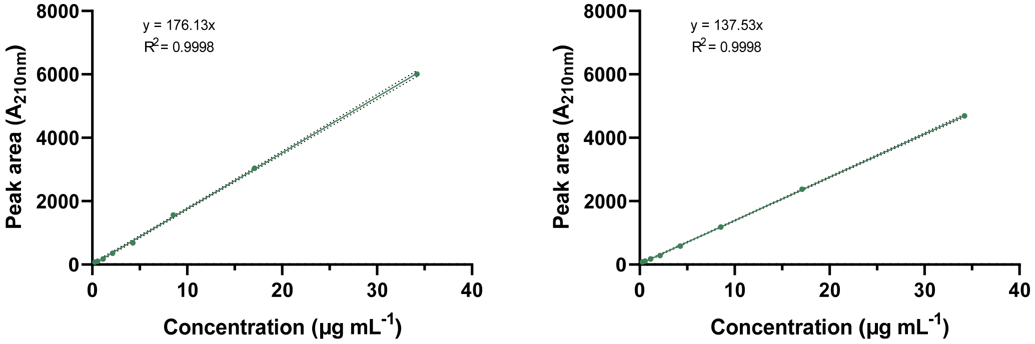

a. **b.**

Figure S92. Calibration curves at 210 nm for **(a)** 3-chlorobenzyl alcohol and **(b)** 4-chlorobenzyl alcohol.

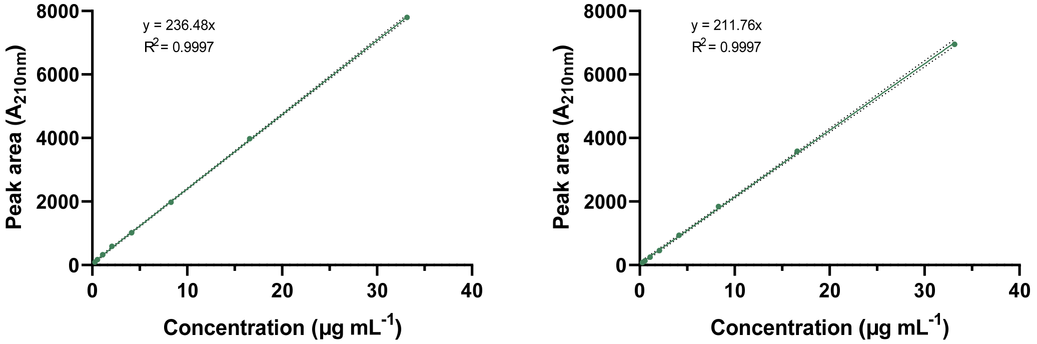

a. **b.**

Figure S93. Calibration curves at 210 nm for **(a)** 2-methoxybenzyl alcohol and **(b)** 3-methoxybenzyl alcohol.

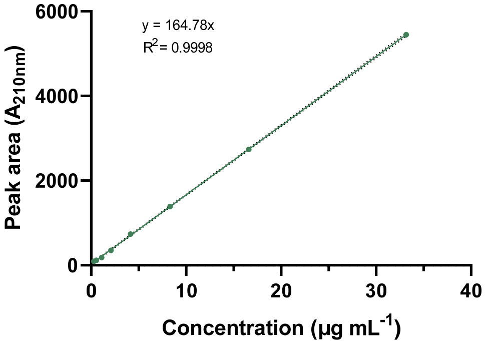

Figure S94. Calibration curve at 210 nm for 4-methoxybenzyl alcohol.

14.3. Process performance indicators for biocatalytic aminations

This section summarizes the key process performance metrics for the biocatalytic aminations studied in this study.

Table S14. Space–time yield (STY) and amine formation in the optimized whole-cell biocatalytic cascade.

Substrate	Substrate concentration (mM)	Yield of amine after 3 h (%)	Isolated yield (%)	STY ($g\ L^{-1}\ h^{-1}$)
**1a**	50	70.7	93	1.17
**1b**	20	76.9	94	0.58
**1c**	30	63.5	92	0.71
**1d**	30	49.6	94	0.57
**1e**	30	78.7	95	1.06
**1f**	20	76.1	93	0.67
**1g**	20	73.6	94	0.65
**1h**	20	79.5	91	0.66
**1i**	30	66.8	90	0.82
**1j**	30	40	92	0.50
**1k**	20	90[a]	95	0.65

[a] – after 4 h of reaction

Table S15. Amine yield and cell-specific productivity as a function of substrate concentration in whole-cell transaminase-catalyzed reactions.

Substrate	Substrate concentration (mM)	Yield of amine after 3 h (%)	Lyophilized $cell_{TA}$ amount (mg)	Productivity per mass of dry cell $weight_{TA}$ (mg g_{DCW}^{-1} h^{-1})
**1a**	10	30.3	5	21.6
**1b**	10	20.6		16.6
**1c**	10	23.6		19.1
**1d**	10	18.2		14.7
**1e**	10	35.0		33.0
**1f**	10	27.0		25.5
**1g**	10	26.4		24.9
**1h**	10	27.0		24.7
**1i**	10	28.6		26.2
**1j**	10	13.0		11.9
**1k**	20	18.0[a]		27.3

[a] – after 4 h of reaction

Table S16. Amine yield and cell-specific productivity as a function of substrate concentration in the optimized whole-cell biocatalytic cascade.

Substrate	Substrate concentration (mM)	Yield of amine after 3 h (%)	Lyophilized $cell_{TA–PDC}$ amount (mg)	Productivity per mass of dry $cell_{TA–PDC}$ weight (mg g_{DCW}^{-1} h^{-1})
**1a**	50	70.7	10	126.3
**1b**	20	76.9		62.1
**1c**	30	63.5		77.0
**1d**	30	49.6		60.1
**1e**	30	78.7		111.4
**1f**	20	76.1		71.8
**1g**	20	73.6		69.5
**1h**	20	79.5		72.7
**1i**	30	66.8		91.6
**1j**	30	40		54.9
**1k**	20	90[a]		68.5

[a] – after 4 h of reaction

Table S17. Catalytic performance (TON) of the purified transaminase.

Substrate	Substrate concentration (mM)	Yield of amine (%)	Purified TA amount (μg mL^{-1})	M_{TA} (g mol^{-1})	TON_{TA} ($\times10^6$)
**1a**	10	63.4	100	52849.96	3.35
**1b**		47.2			2.50
**1c**		34.4			1.82
**1d**		25.5			1.35
**1e**		60.0			3.17
**1f**		43.1			2.28
**1g**		35.9			1.90
**1h**		52.8			2.79
**1i**		38.7			2.05
**1j**		15.5			0.82
**1k**		39.6			2.09

Table S18. Amine yield and catalytic performance (TON) in the *PpS*-TA–*Zm*PDC enzymatic cascade.

**Substrate**	**Substrate concentration (mM)**	**Yield of amine (%)**	**Purified TA amount (μg mL^{-1})**	**M_{TA} (g mol^{-1})**	**$TON_{TA–PDC}$ (×10^6)**
**1a**		83.4			4.41
**1b**		76.2			4.03
**1c**		62.8			3.32
**1d**		49.1			2.60
**1e**		70.8			3.74
**1f**	10	68.5	100	52849.96	3.62
**1g**		55.9			2.96
**1h**		80.9			4.27
**1i**		70.4			3.72
**1j**		33.3			1.76
**1k**		60.8			3.21
